# Managing dry eye disease – a review of selected traditional Chinese medicine and some of their metabolites focusing on molecular mechanisms and signaling pathways

**DOI:** 10.3389/fphar.2026.1693198

**Published:** 2026-03-31

**Authors:** Yixuan Lu, Yuhang Li, Zhen Zhao, Yue Liu, Jiadi Wang, Jing Yao

**Affiliations:** 1 Heilongjiang University of Chinese Medicine, Harbin, China; 2 First Affiliated Hospital of Heilongjiang University of Chinese Medicine, Harbin, China

**Keywords:** dry eye disease, literature review, medical treatment, signaling pathway, traditional Chinese medicine, vicious cycle

## Abstract

Dry eye disease (DED) is a prevalent global health issue that negatively impacts quality of life. It is also associated with the risk of corneal ulceration, scarring, and even blindness in severe cases. The complex, multifactorial pathology of DED, often conceptualized as a vicious cycle involving tear film instability, hyperosmolarity, inflammation, and apoptosis, complicates treatment. Traditional Chinese Medicine (TCM) offers a potential therapeutic approach by targeting multiple components and pathways. This review systematically describes the mechanisms by which TCM-derived metabolites, botanical drug extracts, and TCM formulas are reported to modulate interconnected pathological processes underlying DED to establish a theoretical framework for intervention optimization. A systematic literature search was conducted on 23 April 2025, across seven electronic databases (PubMed, Web of Science, Embase, SinoMed, CNKI, Wanfang, and VIP) using a comprehensive strategy combining MeSH terms and keywords related to DED, TCM, and signaling pathways. DED involves alterations in several important signaling pathways associated with inflammation, oxidative stress, and apoptosis. These include PI3K/AKT, MAPK, NF-κB, NLRP3, Nrf2, SIRT, AMPK, and VEGF. TCM formulas represent a multi-target approach with an ability to simultaneously target single or multiple signaling pathways involved in DED pathology. This is proposed to counteract key interlinked processes of inflammation, apoptosis, and oxidative stress, and promotes tear film stability in cellular and animal models, thereby highlighting its pharmacological potential. The multi-component, multi-target, and multi-pathway approach of TCM may offer a strategic advantage for managing the multifactorial pathology of DED through the potential to simultaneously regulate multiple interactive signaling networks. This review describes the molecular mechanisms underlying these traditional treatment practices and offers a robust foundation for future clinical translation.

## 1 Introduction

Dry eye disease (DED) is a multifactorial, symptomatic disease characterized by a loss of homeostasis of the tear film and/or ocular surface, in which tear film instability and hyperosmolarity, ocular surface inflammation and damage, and neurosensory abnormalities play etiological roles (Wolffsohn et al., 2025). It is a chronic condition that manifests with symptoms like dryness, irritation, burning, and blurred vision, and negatively impacts quality of life (Hakim and Farooq, 2022). DED is a major public health concern, its prevalence based on signs and symptoms ranges from 4.7% to 62.9% (Stapleton et al., 2025). DED is associated with risk factors that can trigger or exacerbate the condition, including age, gender, genetics, hormones, systemic or ocular diseases, medications, ocular surgery, lifestyle, and environmental conditions (Britten-Jones et al., 2024). While the classic vicious-cycle model describes a chronic inflammatory state, clinical presentations are heterogeneous; for instance, transient or seasonal DED may not always exhibit sustained inflammation.

The central pathology of DED is commonly described by a complex vicious cycle of “tear film instability-tear hyperosmolarity-inflammation-apoptosis”, wherein each component can exacerbate the others (Baudouin et al., 2013; Rhee and Mah, 2017). Dysfunction in the lacrimal glands, meibomian glands, cornea, and conjunctiva can lead to abnormal composition or volume of tears, thereby destabilizing the tear film and increasing its osmolarity (Bron et al., 2017). Tear hyperosmolarity imposes stress on the epithelial cells of the ocular surface, which is associated with activation of mitogen-activated protein kinase (MAPK) signaling pathways, leading to elevated production of inflammatory mediators such as cytokines, matrix metalloproteinases (MMPs), and chemokines (Luo et al., 2005). These inflammatory agents promote maturation of antigen-presenting cells (APCs) and subsequent release of high concentrations of cytokines, which contribute to epithelial cell damage by engaging the adaptive immune response and sustaining inflammation (Chu et al., 2024; Rhee and Mah, 2017). Collectively, these processes can promote cellular apoptosis, thereby leading to loss of conjunctival goblet cells, decreasing the mucin output, exacerbating tear film rupture, perpetuating a vicious cycle. Oxidative stress is considered an important contributor to this vicious cycle (Seen and Tong, 2018). Higher levels of reactive oxygen species (ROS) can cause cell death by directly damaging cellular DNA, lipids, and proteins, and activating the MAPK/NF-κB pathway to promote production of downstream cytokines (Bu et al., 2024). The “ROS-inflammation-ROS cross-amplification” effect represents a vicious cycle wherein pro-inflammatory cytokines stimulate the production of ROS, which in-turn further amplifies inflammation, creating a self-perpetuating loop (Han Yu et al., 2023). This cycle involves classical and alternative cell death mechanisms, including apoptosis, ferroptosis, pyroptosis, and autophagy-mediated cell death, which are implicated in the vicious cycle of DED. Acyl-CoA synthetase long-chain family 4 (ACSL4)-mediated lipid peroxidation and depletion of glutathione peroxidase 4 (GPX4) can trigger ferroptosis, thereby contributing to corneal epithelial damage (Zuo et al., 2022). The hyperosmolar tears can induce pyroptosis in the corneal epithelial cells by activating the Nod-like receptor protein 3 (NLRP3) inflammasome pathway, characterized by Caspase-1/11-induced gasdermin D (GSDMD)-mediated cell lysis (Zhang J. et al., 2021). Autophagy is typically regarded as a pro-survival mechanism during oxidative and inflammatory stress and involves degradation of cellular debris and damaged organelles through the lysosome-dependent degradation pathway (Chen H. et al., 2025; Liao et al., 2024). However, under certain environmental conditions, autophagy may also contribute to cell death through selective degradation of proteins or organelles that are critical for cell survival and directly participates in the cell death mechanisms (Huang et al., 2025).

Conventional (hereafter referring to mainstream, evidence-based) therapies such as artificial tears, anti-inflammatory drugs, immunomodulators, and physical therapy can alleviate symptoms of DED. However, these often act by targeting a limited set of mechanisms and can cause recurring problems and potential long-term drawback because of their inability to adequately address the multifactorial pathological processes involved in DED (McCann et al., 2024; Messmer et al., 2023). In contrast, traditional Chinese medicine (TCM) offers a complementary approach with potential advantages for the management of DED. The active metabolites of TCM have been shown to protect conjunctival goblet cells, maintain tear film stability, and promote tear secretion by simultaneously inhibiting inflammation, apoptosis, and ROS generation (Hu et al., 2025). The multi-component nature of TCM prescriptions allows for synergistic multi-target effects that aim to disrupt the vicious cycle of DED and exert overall regulatory influence. However, limitations such as complexity of components, ambiguous mechanisms, and slow onset remain as drawbacks for practical application of TCM. Therefore, this article systematically reviews the research progress on the signaling pathways underlying TCM interventions in DED to clarify the mechanistic relationships between TCM metabolites, targets, and observed pharmacological effects, and explores a novel combined treatment strategy of both TCM and Western medicine to improve management of DED.

It is important to clarify that many of the bioactive metabolites discussed in this review, such as flavonoids, polyphenols, and alkaloids, are widely distributed throughout the plant kingdom and are not exclusive to Traditional Chinese Medicine. They are included here because they represent key constituents of specific TCM botanical drugs and are central to the pharmacological activities reported for the corresponding TCM extracts and formulas. This review therefore focuses on these selected metabolites within the context of their role in TCM-based interventions for DED, while acknowledging their broader presence in nature.

The holistic, multi-target approach is a central tenet not only of TCM but of many global Traditional Medicine (TM) systems, such as traditional Indian (Ayurveda) medicine, and traditional Arabic (Unani) medicine, each with distinct theoretical foundations. This review focuses specifically on TCM to provide a coherent and in-depth analysis of the modern scientific evidence elucidating its mechanisms in DED. This focused scope allows for a systematic exploration of how TCM’s unique paradigm—from its botanical agents to its complex formulas—interacts with the molecular pathophysiology of DED. Such a detailed synthesis within one system is a necessary step for meaningful translational research and offers a template for future studies of other TM systems.

However, it is crucial to note that the current evidence base consists predominantly of preclinical studies. The diversity in experimental models, methodological quality, and the degree of standardization of TCM agents means that while the mechanistic insights are promising, definitive conclusions regarding clinical efficacy require cautious interpretation and further validation. This review synthesizes these mechanistic findings while acknowledging these context-setting limitations, which are discussed in detail in subsequent sections.

## 2 The foundational principles of TCM

TCM is a comprehensive medical system rooted in a distinct theoretical framework that emphasizes balance and interconnection. Its fundamental nature is holistic, viewing the human body as an integrated whole where physiological and pathological states result from the dynamic equilibrium of internal systems and their interaction with the external environment. A core clinical principle is “treatment based on syndrome differentiation”, whereby therapy is tailored not merely to a disease label but to the specific pattern of disharmony (“syndrome”) unique to the individual patient. This leads to a therapeutic paradigm fundamentally different from conventional single-target pharmacology. TCM interventions, typically comprising complex botanical formulas, are designed to act through multiple bioactive components. These components collectively modulate a network of biological targets and pathways, aiming to restore systemic homeostasis. This multi-component, multi-target, and multi-pathway approach aligns with the complex, interconnected pathophysiology of chronic diseases like DED, making TCM a valuable paradigm for exploring integrative treatment strategies. The subsequent sections will examine the scientific evidence for how specific TCM-derived agents interact with the molecular networks underlying DED.

## 3 The vicious cycle of DED: a conceptual framework

This section outlines the disease process using the classic conceptual framework of a “vicious cycle,” in which core pathological events—tear film instability, tear hyperosmolarity, inflammation, and apoptosis—engage in reciprocal interactions that drive disease chronicity (Baudouin et al., 2013). It is important to note that the “inflammation” within this framework specifically refers to sustained or dysregulated inflammatory responses that have escaped normal self-limiting mechanisms. The following sections detail the role of each component within this interconnected model.

### 3.1 Tear film instability

Tear film is a complex, triple-layered liquid structure covering the front surface of the eyeball. The outermost lipid layer is produced by the meibomian glands and acts as a barrier against evaporation while preserving surface tension. The middle aqueous layer is secreted by the lacrimal functional unit (LFU) consisting of the lacrimal glands, cornea, conjunctiva, and meibomian glands. It contains water, electrolytes, lysozyme, and immunoglobulins, and is associated with lubrication, immune defense, and ocular nourishment functions (Stern et al., 2004). The innermost mucin layer contains both secretory mucins (MUC2, MUC5AC, MUC5B, MUC7) and transmembrane mucins (MUC1, MUC4, MUC16), which interact directly with the corneal and conjunctival epithelial cells (Spurr-Michaud et al., 2007). Secretory mucins are mainly secreted by the goblet cells and form a mucinous gel to maintain stability of the tear film by anchoring it to the ocular surface (Hollingsworth and Swanson, 2004). Transmembrane mucins, also known as membrane-associated mucins, are expressed on the surface of corneal and conjunctival epithelial cells. They act by converting the hydrophobic ocular surface into a hydrophilic surface and promote uniform spread of the aqueous layer (Gipson et al., 2016). Their transmembrane structural domain participates in intercellular signal transduction and barrier protection (Ma et al., 2018) and forms an epithelial cell glycocalyx to prevent pathogen adherence (Pflugfelder and Stern, 2020). A few detached membrane-associated mucins form the soluble lacrimal mucus (Ablamowicz and Nichols, 2016).

The contemporary understanding of DED, as per the TFOS DEWS III (2025) report, emphasizes an etiology-driven subclassification to guide targeted therapy, moving beyond the traditional categorization into aqueous-deficient, evaporative, and mixed types (Wolffsohn et al., 2025). This refined framework identifies specific abnormalities in tear film components (e.g., lipid, aqueous, mucin/glycocalyx layers) and ocular surface/functional units. For instance, hyper-evaporative DED is primarily driven by meibomian gland dysfunction (MGD), wherein obstruction or detachment of the meibomian glands impair lipid secretion, thereby destabilizing the tear film and accelerating evaporation (Nelson et al., 2011; Sheppard and Nichols, 2023). Furthermore, LFU dysfunction caused by surgical damage or chronic conditions like diabetes disrupts the ocular surface epithelial barrier, reduces tear secretion or modifies the tear composition, destabilizes the tear film, and compromises ocular surface protection (Pflugfelder and de Paiva, 2017; Pflugfelder and Stern, 2020). Interferon (IFN)-γ reduces conjunctival goblet cell density and expression levels of MUC5AC and MUC2 (Pflugfelder et al., 2015). Loss of conjunctival goblet cells destabilizes the tear film and correlates with inflammation severity and symptom intensity (Liu Y. et al., 2024). Shortened tear film break-up time (TBUT) further exacerbates DED development by exposing the cornea to external stimuli and impairs the ocular surface microenvironment and epithelial barrier.

### 3.2 Tear hyperosmolarity

Tear hyperosmolarity (THO) is a central pathological process in DED and is closely interconnected with other components of the vicious cycle. Critically, THO is not a static state but a dynamic and variable process. This dynamism is best described by the concept of “*osmokinetics*”— which refers to the temporal patterns, oscillations, and rates of change in tear film osmolarity resulting from the interplay of evaporation, secretion, and clearance (van Setten, 2020; Stahl et al., 2012). Tear film instability leads to excessive evaporation, concentrating solutes (e.g., Na^+^, Cl^−^, proteins) and creating localized, transient osmotic stress on the ocular surface epithelium. When compensatory tear secretion or refreshment is insufficient, this stress becomes sustained. Normal tear osmolarity is approximately 302 ± 9.7 mOsm/L, and hyperosmolarity is often defined as exceeding 316 mOsm/L (Tomlinson et al., 2006). Ocular surface epithelial cells exposed to this dynamic osmotic challenge activate rapid, adaptive stress-response signaling, including the MAPK and NF-κB cascades, and modulate osmolarity-related proteins like Na^+^/K^+^-ATPase and aquaporins (AQPs) in an attempt to restore homeostasis.

Prolonged hyperosmolarity inhibits AQP5 and MUC5AC expression in the conjunctiva and reduces anchoring ability of the mucin layer (Bhattacharya et al., 2017). It also downregulates human corneal limbal transmembrane mucin MUC16, thereby exacerbating tear film stratification and corneal dehydration (Panigrahi et al., 2023). Sustained hyperosmolarity can drive the inflammatory cascade in DED and contribute to the disruption of the corneal epithelial barrier, in part through the activation of the MAPK and NF-κB pathways, thereby resulting in the secretion of interleukin (IL)-6, IL-1β, TNF-α, IL-8, and MMP-9 (Luo et al., 2005; Pan et al., 2011). Transient hyperosmolarity causes ocular surface nerve injury and disrupts immune homeostasis by activating the NF-κB signaling pathway, increasing the number and activity of dendritic cells (DCs), and enhancing the generation of CD4^+^ T cells in the ocular lymph nodes; concurrently, it depletes intraepithelial corneal nerves and nerve endings by hypersensitizing the ocular surface to hyperosmolarity stress (Guzmán et al., 2020). Hyperosmotic stress induces GSDMD-dependent pyroptosis in the human corneal epithelium by enhancing GSDMD levels and cleaved Caspase-1 activity, and triggers apoptosis by regulating Bcl-2-associated X protein (Bax) and B-cell lymphoma/leukemia-2 (Bcl-2) expression levels and Caspase-3 activation (Li J. et al., 2022). Hyperosmolarity in the corneal epithelial cells causes ROS accumulation, reduced total antioxidant capacity, and apoptosis by reducing mitochondrial membrane potential and increasing cytoplasmic Ca^2+^ levels (Yang et al., 2016).

### 3.3 Inflammation

Inflammation is recognized as a major contributor to initiating and perpetuating the vicious cycle of chronic DED. It is critical to note that within this pathological context, “inflammation” refers specifically to a sustained, dysregulated state that has escaped normal self-limiting mechanisms, distinct from transient, protective immune responses. This maladaptive process involves significant activation of both innate and adaptive immune arms. Typically, the inflammatory cascade is initiated by the generalized activation of the innate immune system, which is then followed and amplified by a more specific adaptive immune response (Yu et al., 2021).

The innate immune defense on the ocular surface involves the corneal and conjunctival epithelial cells, and antigen-presenting cells (APCs) such as dendritic cells, neutrophils, monocytes, and macrophages (Pflugfelder and de Paiva, 2017). In a healthy state, immature APCs reside in the ocular surface epithelium to continuously sample antigens and maintain immune tolerance. However, in DED, stimuli such as dryness, hyperosmolarity, or ROS activate ocular surface epithelial cells and APCs. The activated APCs release inflammatory signals such as chemokines, cytokines (IFN-α, IL-1, IL-1β, IL-6, and TNF-α), and matrix metalloproteinases (MMPs). These molecules amplify the immune responses, recruit inflammatory cells, promote ROS generation, and activate sensory nerves, resulting in DED-related discomfort. This neuro-immune crosstalk is critical and evolves into a defined process of neurogenic inflammation: activated sensory nerves release neuropeptides (e.g., Substance P, CGRP), which induce vasodilation, plasma extravasation, and further potentiate immune cell activity, thereby establishing a self-sustaining loop that significantly contributes to ocular pain and surface damage (Asiedu, 2022).

In DED, desiccation stress can lead to generation of self-antigens via proteolytic breakdown or disrupted cell differentiation. These self-antigens are phagocytosed by the mature APCs, which then travel to nearby lymph nodes and activate naive T cells, thereby stimulating the adaptive immune response (Niederkorn et al., 2006; Stern and Pflugfelder, 2017). Activated CD4^+^ T cells develop into specialized subsets, including Th1 and Th17 cells. Th1 cells secrete IFN-γ to antagonize IL-13 and promote epithelial apoptosis and abnormal squamous changes. Th17 cells recruit monocytes and neutrophils by releasing IL-17, exacerbate inflammation, and upregulate the expression and secretion of pro-inflammatory signaling molecules and MMPs, thereby worsening epithelial damage (Chen and Dana, 2021). Chronic DED reduces the dryness pressure threshold for DED episodes because of a persistent and established adaptive immune response perpetuating inflammation (De Paiva et al., 2009).

### 3.4 Apoptosis

Ocular surface cell apoptosis in DED is primarily triggered by high osmolarity, inflammation, and oxidative stress. Elevated tear osmolarity can damage corneal and conjunctival epithelial cells by inducing endoplasmic reticulum stress and the subsequent activation of apoptotic pathways (Guindolet et al., 2022). This affects their normal function and overall activity, upregulates pro-apoptotic genes, and triggers inflammation and oxidative stress to induce cellular apoptosis (Li J. et al., 2022; Scarpellini et al., 2023). Apoptosis constitutes the main mechanism of programmed cell death and is required for eliminating redundant or aberrant cells through specific molecular events. DNA fragmentation is the characteristic feature of apoptosis and marks irreversible termination of cell function (Kerr et al., 1972).

Apoptosis involves both extrinsic and intrinsic signaling pathways. The extrinsic apoptotic pathway involves interactions between extracellular ligands (e.g., TNF, FASL, TL1A, and TRAIL) and the corresponding transmembrane death receptors (TNFR, FAS, DR3, and DR4/DR5) (Newton et al., 2024). Extrinsic apoptosis involves assembly of the death-inducing signaling complex (DISC) through FADD recruitment via death domain interactions (Bedoui et al., 2020), activation of the initiator Caspases-8 and -10, and subsequent triggering of the effector Caspase-3/7 cascade (Stennicke et al., 1998). The intrinsic apoptotic pathway is activated by cellular stress signals such as oxidative stress, hyperosmotic stress, and inflammatory stress, which cause mitochondrial membrane depolarization (Shankaranarayanan et al., 2009). Furthermore, upregulated BH3 proteins bind to anti-apoptotic proteins such as Bcl-2 and B-cell lymphoma-extra-large (Bcl-xl), thereby liberating pro-apoptotic proteins like Bax and Bcl-2 antagonist/killer 1 (BAK1); subsequent oligomerization of Bax and BAK1 proteins via BH3 interaction domain death agonist (BID) leads to the formation of the mitochondrial permeability transition pores (MPTPs) and increases the mitochondrial outer membrane permeability (MOMP) (Moldoveanu and Czabotar, 2020). This leads to release of cytochrome C (Cyt C) into the cytoplasm, formation of apoptosome with the apoptotic protease activating factor 1 (APAF-1), activation of initiator Caspase-9, and subsequent execution of apoptosis through activation of the downstream Caspases-3 and -7 (Inoue et al., 2009). Concurrently, endoplasmic reticulum (ER) stress induces protein misfolding and calcium dysregulation activates Caspase-12-mediated apoptosis (Nakagawa et al., 2000).

Uncontrolled or excessive apoptosis worsens DED through compromised corneal epithelial barrier, aggravated inflammation, and sustained cell damage-apoptosis cycle (Li J. et al., 2024). Furthermore, apoptotic cells release damage-associated molecular patterns (DAMPs), including double-stranded DNA, which trigger innate immune response via Toll-like receptor 4 (TLR4) and NLRP3 inflammasome (Wang et al., 2021; Yang et al., 2021) and promote recruitment of neutrophils, macrophages, natural killer cells, and T cells (Nair et al., 2021). Therefore, apoptosis is considered a significant mechanism implicated in DED pathogenesis.

### 3.5 Oxidative stress

Oxidative stress is widely considered an upstream contributor to the vicious cycle in DED. It is caused by an imbalance between antioxidative defense mechanisms and pro-oxidant factors, and is characterized by an overabundance of ROS (Seen and Tong, 2018). Mitochondrial electron transport chain (METC) is the primary source of intracellular ROS, which are released into cytoplasm because of increased mitochondrial membrane permeability (Nolfi-Donegan et al., 2020). Ocular surface protection against ROS involves a combination of non-enzymatic antioxidants such as lactoferrin, S100A protein, nuclear factor erythroid-2-related factor 2 (Nrf2), glutathione (GSH), and vitamin C/E, as well as antioxidant enzymes such as superoxide dismutase (SOD), catalase (CAT), GPX, glutathione S-transferase P1 (GSTP1), and heme oxygenase-1 (HO-1) (Böhm et al., 2023; Perez-Garmendia et al., 2020).

Under normal physiological conditions, ROS homeostasis is maintained through an intricate balance between ROS production and elimination mechanisms. In DED, ROS levels are elevated because of prolonged exposure to atmospheric oxygen and depletion of antioxidant levels due to instability of the tear film (Dogru et al., 2018). Hyperosmotic stimuli and apoptosis significantly increase ROS levels by disrupting mitochondrial membrane integrity, which causes leakage of the electron transport chain. These ROS molecules generate malondialdehyde (MDA) by oxidizing polyunsaturated fatty acids (e.g., linoleic acid) in the meibomian gland lipids, thereby decreasing lipid layer hydrophobicity and accelerating tear evaporation (Choi et al., 2016). ROS upregulates pro-inflammatory genes, including TNF-α, IL-6, and IL-1β, by activating NF-κB and MAPK (e.g., c-Jun N-terminal kinase (JNK)/p38) pathways (Liu J. et al., 2023). This directly stimulates activation of NLRP3 inflammasome-mediated Caspase-1 and subsequent release of IL-1β/IL-18, thereby increasing inflammation. IL-1β promotes ROS generation by significantly affecting endogenous antioxidant enzymes such as SOD and CAT, thereby contributing to a vicious cycle of ROS and inflammation (Dominic et al., 2022). Furthermore, elevated levels of ROS cause cellular death by oxidizing biological macromolecules such as DNA, proteins, and lipids (Dogru et al., 2018). They also influence T-cell receptor signaling pathways, driving the polarization of naive T cells towards the pro-inflammatory Th17 lineage, thereby reinforcing inflammation through the combined action of IL-17A and IL-1β (Zheng Y. et al., 2024). Therefore, elimination of excessive ROS is an important therapeutic strategy for managing DED.

## 4 Methods

### 4.1 Literature search strategy

A systematic literature search was conducted across seven electronic databases on 23 April 2025, with no start date restriction. The databases included three international sources (PubMed, Web of Science, Embase) and four Chinese sources (SinoMed, CNKI, Wanfang, and VIP).

The search strategy was designed to encompass three core concepts: DED, TCM, and signaling pathways. Customized Medical Subject Headings (MeSH) terms and free-text keywords were applied to each database. Key search terms included: “dry eye disease,” “signaling pathway,” “traditional Chinese medicine,” “herbal monomers,” “herbal extracts,” and “herbal formulas”.

After the initial search and screening, to ensure the comprehensiveness of the review, we used unstructured supplementary search as an auxiliary means, including manual review of relevant field journals and references of the review. All studies discovered in this way and ultimately meeting the inclusion criteria have been included in the final literature pool. Supplementary Material 1 outlines the exhaustive and explicit search methodologies for all databases.

### 4.2 Study selection and eligibility criteria

**FIGURE 1:**
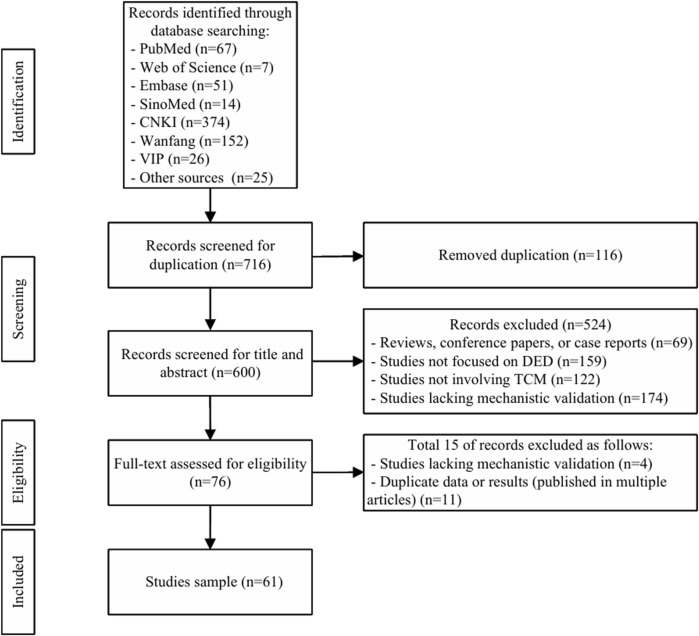
PRISMA flow diagram of study selection.

The process of selecting studies adhered to the Preferred Reporting Items for Systematic Reviews and Meta-Analyses (PRISMA) guidelines, as depicted in Figure 1. Both YL (Yixuan Lu) and YL (Yuhang Li) meticulously carried out the literature screening, study selection, and data extraction, ensuring their work was entirely independent. Should any discrepancies arise, they would hashed them out or seek the assistance of a third reviewer, ZZ, to reach a consensus. The records from “Other sources (n = 25)” in the flowchart were identified through manual searches. The synthesis of evidence into summary tables (Tables 1–3) and Supplementary Tables S1–S4 was performed through a multi-author, manual curation process to ensure accuracy and critical appraisal.

**TABLE 1:** Critical evidence synthesis of TCM interventions targeting key signaling pathways in dry eye disease.

Pathway	Exemplary TCM interventions (Type)^a^	Evidence summary	Evidence strength (*in vivo*/*in* *vitro*)^b^	Representative studies^d^	Critical notes
NF-κB Pathway Inhibition	Berberine (M), KIOM-2015E (E), Qingxuan Runmu Decoction (F)	Consensus: Extensive direct evidence across models	●●●/●●●	*In vivo*: (Li, 2020) *In vitro*: (Han et al., 2023a)	Well-validated; chemical standardization needed
NLRP3 Inflammasome Inhibition	Oridonin (M), Polydatin (M), Runmu Ling Granules (F)	Consensus: Direct inhibition of inflammasome assembly	●●●/●●●	*In vivo*: (Li X. et al., 2024) *In vitro*: (Li X. et al., 2024)	Strong preclinical target; clinical translation studies needed
MAPK Pathway Modulation	Kaempferol (M), Paeoniflorin (M), Qishen Prescription (F) [Inhibition]; Polydatin (M) [Activation]	Note: Mostly inhibition; one activation study	●●○/●●●	Activation *In vivo*: (Cao et al., 2024) Inhibition *In vivo*: (Zhao et al., 2019) Inhibition *in vitro*: (Li D., 2021)	Direction of modulation requires clarification
SIRT Activation	Puerarin (M), Oroxylin A (M), Salidroside (M)	Consensus: Direct activation supports stress resistance and anti-inflammation	●●○/●●●	*In vivo*: (Liang et al., 2023) *In vitro*: (Dong et al., 2024)	Further *in vivo* mechanistic confirmation in ocular tissues required
Nrf2 Antioxidant Pathway Modulation	Esculetin (M), Astaxanthin (M), Gallic Acid (M) [Activation]; Acteoside (M) [Inhibition]	Note: Contradictory *in vitro*; limited *in vivo*	●●○/●●○	Activation *In vivo*: (Liu Z. et al., 2025) Activation *in vitro*: (Zhang et al., 2021a) Inhibition *in vitro*: (Huang and Peng, 2024)	Role of Nrf2 inhibition unclear; consistent *in vivo* validation needed
PI3K/AKT Pathway Modulation	Quercetin (M) [Inhibition], Mimeng Hua Eye Drop (E) [Activation]	Note: Bidirectional modulation reported	●●○/●●○	Activation *In vivo*: (Qin et al., 2019) Inhibition *In vivo*: (Han et al., 2023a) Inhibition *in vitro*: (Huang et al., 2024)	Determinants of modulation direction undefined
AMPK Pathway Modulation	Salidroside (M) [Activation], Chlorogenic Acid (M) [Inhibition]	Note: Bidirectional modulation reported	●●○/●●○	Activation *In vivo*: (Liang et al., 2023) Inhibition *In vivo*: (Chen H. et al., 2025) Activation *in vitro*: (Liang et al., 2023)	Inconsistent findings on activation/inhibition; pathophysiological context requires elucidation
MUC1/EGFR Activation	Astragaloside IV (M)	Consensus: Direct barrier enhancement; single agent	●●○/●●○	*In vivo*: (Chu et al., 2021) *In vitro*: (Chu et al., 2021)	Target clinically relevant; more agents needed
p53/mTOR Modulation	Evodiamine (M)	Consensus: Direct autophagy regulation via p53 activation and mTOR inhibition	●●○/●●○	*In vivo*: (Li et al., 2025) *In vitro*: (Li et al., 2025)	Role in DED needs definition
HMOX1/HIF-1 Inhibition	Qingxuan Runmu Decoction (F)	Consensus: Single formula links to oxidative stress and ferroptosis	●●○/●●○	*In vivo*: (Wang J. et al., 2024) *In vitro*: (Wang J. et al., 2024)	Mechanistic role and generalizability require validation
VEGF Inhibition	EGCG (M), Qinpi Eye Drop (E)	Consensus: Direct *in vivo* VEGF isoform inhibition	●●●/○○○	*In vivo*: (Lee et al., 2011; Hu, 2017)	Limited to two agents; VEGF isoform roles need clarification
PERK-eIF2α-ATF4-CHOP Pathway Inhibition	Xiaosheng Granules (F)	Note: Direct *in vitro*; *in vivo* only phenotypic	●○○/●●○	*In vitro*: (Du, 2018)	Promising; *in vivo* pathway validation needed
Bax/Caspase-9/Caspase-3 Inhibition	Modified Siwei Dafa Powder (F)	Consensus: Direct *in vivo* intrinsic apoptosis inhibition	●●○/○○○	*In vivo*: (Chen L. et al., 2023)	Limited to one formula; *in vitro* corroboration needed
PKA/CREB Activation	Polydatin (M)	Consensus: Single *in vivo* study links activation to clinical signs	●●○/○○○	*In vivo*: (Zhao and Wei, 2024)	Independent validation needed
TRAF6/TAK1 Inhibition	Qingxuan Runmu Decoction (F)	Consensus: Direct *in vivo* upstream inflammatory inhibition	●●○/○○○	*In vivo*: (Zhao et al., 2024a)	Limited to one formula; broader validation needed
TLR4/MyD88 Inhibition	Qingxuan Runmu Decoction (F)	Consensus: Direct *in vivo* innate immune pathway inhibition	●●○/○○○	*In vivo*: (Zhao et al., 2024b)	Validation with other agents required
IRAK1/TRAF6 Inhibition	Runmu Xiaoyao Powder (F)	Consensus: Single *in vivo* study suggests neuroimmune role	●●○/○○○	*In vivo*: (Liu T. et al., 2024)	Novel axis; replication needed
Caspase-8/Caspase-3 Inhibition	Zhenzhu Mingmu Eye Drops (F)	Consensus: Direct *in vitro* extrinsic apoptosis inhibition	○○○/●●○	*In vitro*: (Wu et al., 2022)	*In vivo* validation lacking
JNK1/AQP5 Pathway Inhibition	Qishen Prescription (F)	Consensus: Direct *in vitro* JNK1-AQP5 link	○○○/●●○	*In vitro*: (Zhao et al., 2022a)	*In vivo* confirmation needed
STAT1 Activation	Buddleia Flavonoids (E)	Consensus: Direct *in vitro* activation as a protective role in stressed LG cells	○○○/●●○	*In vitro*: (Wang et al., 2010)	Limited to one cell type/stressor; *in vivo* significance unconfirmed
Notch Activation	Resveratrol (M)	Consensus: Single *in vitro* activation in stressed corneal epithelial cells	○○○/●●○	*In vitro*: (Shetty et al., 2020)	*In vivo* confirmation needed
FcγR-mediated phagocytosis Inhibition	Modified Danzhi Xiaoyao Powder (F)	Note: Functional *in vivo* evidence of reduced phagocytosis	●○○/○○○	*In vivo*: (Liu et al., 2024b)	Functional evidence; direct proof needed

^a^ Intervention type: (M) Pure metabolite; (E) Standardized botanical drug extract/formulation; (F) Multi-herb TCM, formula.

^b^ Evidence strength: ●●● Strong (≥2 direct, consistent studies); ●●○ Moderate (single direct study, contradictory direct studies, or ≥2 indirect/functional/phenotypic studies); ●○○ Preliminary (single indirect/functional/phenotypic study); ○○○ No available evidence. *In vivo* and *In vitro* ratings synthesized separately from evidence in Supplementary Table S1.

^c^ Evidence Quality classification used in Tables 2, 3, and Supplementary Table S1: Direct = direct measurement of pathway activity (e.g., Western blot, immunofluorescence for phospho-proteins, nuclear translocation); Indirect = measurement of downstream effectors (e.g., cytokines, oxidative stress markers); Functional assay = measurement of downstream cellular/immune function (e.g., phagocytosis assay); Phenotypic Only = only clinical/therapeutic outcomes measured.

^e^ Column functions for Table 1: “Evidence Summary” uses “Consensus:” for well-established findings and “Note:” for pathways requiring special consideration; “Critical Notes” provides specific limitations or research gaps.

**TABLE 2:** *In vivo* pharmacological effects and mechanistic support for TCM interventions in DED animal models.

TCM intervention (Type)^a^	Animal model (Induction)	Dose (Route)^b^	Primary outcomes^c^	Key pathway modulation	*In* *vivo* validation (Evidence quality)^d^	Ref.
Qinpi eye drop (E)	Rabbit (autoimmune DED)	Eye drops (topical)	• ↑ SIT, TBUT • ↑ LG weight, conjunctival goblet cell density (GCD) • ↓ Corneal FL scores	PI3K/AKT/mTOR↓	Direct evidence PI3K/AKT pathway • ↓ p-AKT (WB on LG) Immune regulation • ↓ ROR-γt, ↑ Foxp3 (WB on LG)	Jiang et al. (2023)
Mimeng Hua eye drop (E)	Rabbit (castration)	1.0, 1.5, 3.0 mg/mL (topical)	• ↑ SIT • Improved LG histology (HE)	PI3K/AKT ↑	Direct evidence PI3K/AKT pathway • ↑ PI3K, AKT (IHC on LG) Apoptosis • ↓ Caspase-9 (IHC on LG)	Qin et al. (2019)
Polydatin (M)	Rat (SCOP)	0.05%, 0.5% (topical)	• ↑ Tear secretion (phenol red thread) • ↑ TBUT • ↑ GCD [Periodic Acid-Schiff (PAS)] • ↓ Corneal FL scores	MEK/ERK↑	Direct evidence MAPK pathway • ↑ p-MEK1/2/MEK1/2, p-ERK1/2/ERK1/2 (WB on cornea/conjunctiva) Inflammatory markers • ↓ TNF-α, IFN-γ, IL-1β (qRT-PCR on cornea/conjunctiva)	Cao et al. (2024)
Berberine (M)	Mouse (SCOP + DS)	2 mg/mL (topical)	• ↑ Tear secretion (phenol red thread • ↑ GCD (PAS) • ↓ Corneal barrier dysfunction [Oregon Green Dextran (OGD)] • ↓ Corneal epithelial apoptosis (TUNEL)	NF-κB↓	Direct evidence NF-κB pathway • ↓ p-IKK, p-NF-κB, p-IκBα (WB on conjunctiva) Inflammatory markers • ↓ IL-1α, IL-1β, IL-6, IL-17A, IFN-γ, TNF-α (qRT-PCR on conjunctiva) MMPs • ↓ MMP-3, MMP-9 (WB/IF on cornea/conjunctiva) Apoptosis • ↓ Caspase-8 (WB/IF on cornea/conjunctiva) Immune cells • ↓ CD4^+^ T cells (WB/IF on cornea/conjunctiva)	Li (2020)
Paeonol (M)	Mouse (SCOP + DS)	1%, 5%, 10% (topical)	• ↑ Tear secretion (phenol red thread) • ↑ GCD (PAS) • ↓ Corneal barrier dysfunction (OGD) • ↓ Corneal/conjunctival epithelial apoptosis (TUNEL)	NF-κB↓	Direct evidence NF-κB pathway • ↓ p-NF-κB (WB on conjunctiva) Inflammatory markers • ↓ IFN-γ, TNF-α, IL-17A, ↑ IL-13 (qRT-PCR on conjunctiva) MMPs • ↓ MMP-3, MMP-9 (IF on cornea/conjunctiva) Apoptosis • ↓ C-Caspase-3, C-Caspase-8 (IF on cornea/conjunctiva) Immune cells • ↓ CD4^+^ T cells (IHC on conjunctiva)	Wu (2019)
PCE Aqueous Extract (E)	Rat (LG excision)	10, 100, 250 mg/kg (oral)	• ↑ Tear secretion (phenol red thread) • ↓ Corneal irregularity score (reflected white ring analysis)	NF-κB↓	Indirect evidence Barrier function • ↑ MUC4 (IHC/qPCR on cornea) Inflammatory markers • ↓ TNF-α, IL-6, MMP9 (qPCR on cornea)	Park et al. (2018)
KIOM-2015E (E)	Mouse (BAC)	0.5, 1 mg/mL (topical); 100 mg/kg (oral)	• ↑ Tear secretion (phenol red thread) • ↓ Lissamine Green B staining score • ↑ Corneal epithelial thickness (histology)	NF-κB↓	Direct evidence NF-κB pathway • ↓ p-p65 (IHC on cornea) Inflammatory markers • ↓ TNF-α, IL-1β, IL-6 (IF/qPCR on cornea)	Yim et al. (2022)
Oridonin (M)	Mouse (BAC)	0.01%, 0.1%, 1% (topical)	• ↑ Tear secretion (phenol red thread) • ↑ TBUT • ↓ FL scores • ↑ Corneal epithelial thickness (HE)	NLRP3/Caspase-1/GSDMD ↓	Direct evidence NLRP3 inflammasome • ↓ NLRP3, Caspase-1, N-GSDMD (WB on cornea) • ↓ NLRP3, Caspase-1 (IHC on ocular surface)	Li X. et al. (2024)
Huji Sheng eye drop (E)	Mouse (BAC)	0.005% (topical)	• ↑ Tear secretion (phenol red thread) • ↑ GCD (HE) • ↓ pathological injury in conjunctival epithelium and LG (HE)	NLRP3/Caspase-1↓	Direct evidence NLRP3 inflammasome • ↓ NLRP3, Caspase-1 (qPCR/WB on conjunctiva/LG) Inflammatory markers • ↓ IL-18, IL-1β (ELISA on serum)	Wu et al. (2024)
PS-CG nanocomposite (M)	Mouse (BAC)	4 µg/mL (topical)	• ↑ Tear secretion • ↓ Corneal FL scores • ↑ Tear fern crystallization • ↑ Corneal epithelial thickness (HE) • ↑ GCD (PAS)	Not assessed	Phenotypic only • Clinical and histological outcomes measured • No *in vivo* pathway marker validation on ocular tissues	Lin et al. (2022)
Esculetin (M)	Mouse (SCOP + DS)	0.002% (topical)	• ↓ Corneal epithelial damage (OGD) • ↓ Corneal/conjunctival epithelial apoptosis (TUNEL) • ↑ GCD (PAS)	Not assessed	Phenotypic only • Clinical and histological outcomes measured • No *in vivo* pathway marker validation on ocular tissues	Zhang et al. (2021a)
Astaxanthin (M)	Mouse (BAC)	100 mg/kg (oral)	• ↑ GCD (PAS) • ↓ Corneal FL scores • ↓ Corneal inflammatory cell infiltration (HE)	Keap1-Nrf2/HO-1↑	Direct evidence Nrf2 pathway • ↓ Keap1, ↑ Nrf2, HO-1 (IF/qPCR on cornea) Inflammatory markers • ↓ MMP-9, IL-1β (IF/qPCR on cornea) • ↓ IL-6, TNF-α (qPCR on cornea) Antioxidant enzymes • ↑ SOD1, SOD2, CAT (qPCR on cornea)	Liu Z. et al. (2025)
Chlorogenic Acid (M)	Mouse (SOD1^−/−^)	50 mg/kg (i.m.)	• ↑ SIT • ↓ Corneal FL scores • ↓ meibomian gland (MG) inflammatory cell infiltration (HE) • ↓ MG epithelial apoptosis (TUNEL)	AMPK/ULK1↓	Direct evidence AMPK pathway • ↓ p-AMPK/AMPK, p-ULK1/ULK1, Beclin1, LC3II/LC3I, ↑ p62 (WB on MG) • ↓ LC3B (IF on MG) Oxidative stress • ↓ ROS (Flow cytometry on MG cells) • ↓ MDA (Assay kit for MG homogenate)	Chen H. et al. (2025)
Salidroside (M)	Mouse (BAC)	0.5, 2 mM (topical)	• ↑ Tear secretion (phenol red thread) • ↓ Corneal FL scores • ↓ Corneal inflammatory cell infiltration (HE) • ↑ Corneal epithelial thickness (HE) • ↓ Corneal/conjunctival epithelial apoptosis (TUNEL)	AMPK/SIRT1↑	Direct evidence AMPK/SIRT1 pathway • ↑ p-AMPK, SIRT1 (WB/IHC on cornea) Autophagy • ↑ LC3II, ↓ SQSTM1 (WB on cornea) • ↑ Autophagosome, autolysosome (TEM on corneal epithelium) Nrf2 pathway • ↑ nuclear Nrf2, HO-1, NQO1 (WB on cornea) Inflammatory markers • ↓ TNF-α, IL-1β, IL-6 (ELISA on cornea/conjunctiva) • ↓ Th17 (Flow cytometry of draining lymph node cells) Oxidative stress • ↑ SOD, CAT, ↓ MDA (Enzyme assay on cornea)	Liang et al. (2023)
EGCG (M)	Mouse (controlled environment chamber)	0.01%, 0.1% (topical)	• ↓ Corneal FL scores • ↓ Corneal epithelial apoptosis (TUNEL)	VEGF↓	Direct evidence VEGF pathway • ↓ VEGF-A, VEGF-D (qPCR on cornea) Inflammatory markers • ↓ CCL2, IL-1β (qPCR on cornea) Immune cells • ↓ CD11b+ cells (IF/cell counting on cornea)	Lee et al. (2011)
Qinpi eye drop (E)	Mouse (controlled environment chamber)	Eye drops (topical)	• ↑ Tear secretion (phenol red thread) • ↑ Tear fern crystallization • Improved corneal epithelial structure (HE) • ↑ GCD (PAS) • ↓ Corneal lymphangiogenesis (LYVE-1+ area, IF/IHC)	VEGF-C/VEGFR-3↓	Direct evidence VEGF pathway • ↓ VEGF-C, VEGFR-3 (WB/qPCR on cornea) Immune cells • ↓ CD4^+^ T cells (IF/IHC on cornea) Inflammatory markers • ↓ IL-1β, IL-6, ↑ TGF-β1, IL-10 (ELISA on serum)	Hu (2017)
Polydatin (M)	Rat (SCOP)	0.05%, 0.5% (topical)	• ↑ Tear secretion (phenol red thread) • ↓ Corneal FL scores • ↑ GCD (PAS) • ↓ Corneal pathological injury	PKA/CREB↑	Direct evidence PKA/CREB pathway • ↑ p-PKA/PKA, p-CREB/CREB (WB on cornea) Inflammatory markers • ↓ IL-1β, TNF-α, IL-6 (ELISA on cornea)	Zhao and Wei (2024)
Evodiamine (M)	Mouse (BAC/atropine)	500 μM, 1 mM (topical)	• ↑ Tear secretion (phenol red thread) • ↓ Corneal FL scores • ↑ GCD (PAS) • ↓ Corneal pathological injury	p53/mTOR↑	Direct evidence p53/mTOR pathway • ↑ nuclear p-p53, ↓ cytoplasmic p-p53, p-mTOR (WB on cornea) Autophagy • ↑ LC3II, ↓ p62 (WB on cornea) Inflammatory markers • ↓ TNF-α, IL-6, IL-1β (WB/qPCR on cornea)	Li et al. (2025)
Astragaloside IV (M)	Rabbit (BAC)	5, 10 µM (topical)	• ↑ SIT, TBUT • ↑ GCD (HE) • Improved corneal/conjunctival histopathology (HE)	MUC1/EGFR↑	Direct evidence MUC1/EGFR pathway • ↑ MUC1 (IHC/IF/ELISA on cornea/conjunctiva) • ↑MUC1, EGFR (WB/qPCR on cornea)	Chu et al. (2021)
Dendrobium water extracts (E)	Rat (SCOP)	200 mg/kg (oral)	• ↑ SIT • ↑ GCD (PAS) • ↓ Corneal FL scores • ↓ Corneal opacity scores	NF-κB↓, MAPKs↓	Direct evidence MAPK/NF-κB pathway • ↓ p-ERK, p-p38, p-p65 (WB on eyeball/LG) Barrier function • ↑ AQP1, AQP5 (WB on LG) MMPs • ↓ MMP-9, MMP-2 (WB on LG)	Ling et al. (2022)
Linarine (M)	Mouse (BAC + chronic pain/tail clamping)	12.5, 25, 50 mg/kg (oral)	• ↑ SIT, TBUT • ↓ Corneal FL scores • ↑ Cornea/LG histology (HE) • ↓ LG cell apoptosis (TUNEL)	MAPK↓, NF-κB ↓	Direct evidence MAPK/NF-κB pathway • ↓ p38, JNK, p65 (IF on cornea) Inflammatory markers • ↓ IL-1β, IL-18 (IF on cornea)	Liu et al. (2024a)
Paeoniflorin (M)	Mouse (hyperosmotic saline)	0.01%, 0.1%, 1% (topical)	• ↑ Tear secretion (phenol red thread) • ↓ Corneal FL scores • ↓ Detachment of corneal epithelial cells (HE)	MAPK↓, NF-κB↓	Direct evidence (MAPK) MAPK pathway • ↓ p-JNK, p-p38 (IHC on ocular surface/cornea) Indirect evidence (NF-κB) Inflammatory markers • ↓ IL-1 (IHC on ocular surface/cornea)	Zhao et al. (2019)
Aurantio-obtusin (M)	Rat (BAC)	0.5% (topical)	• ↑ Tear secretion (phenol red thread) • ↓ Conjunctival irritation score (edema, hyperemia, secretion) • ↑ GCD (PAS) • ↓ Corneal FL scores	NF-κB↓, NLRP3↓	Direct evidence NF-κB pathway • ↓ p-IKKβ, p-IκBα, p-p65 (WB on conjunctiva/cornea) NLRP3 inflammasome • ↓ NLRP3 (WB/IHC on conjunctiva/cornea) • ↓ ASC, cleaved Caspase-1, IL-1β (WB on conjunctiva/cornea) Inflammatory markers • ↓ TNF-α, IL-6, MCP-1, ↑ IL-10 (ELISA on conjunctiva/cornea)	Zhu et al. (2024)
Polydatin (M)	Rat (LG excision)	0.05%, 0.5% (topical)	• ↑ Tear secretion (phenol red thread) • ↑ TBUT • ↓ Corneal irregularity score (white ring reflection) • ↓ Lissamine Green staining scores • ↑ GCD (PAS)	NF-κB ↓, NLRP3↓	Direct evidence (NLRP3) NLRP3 inflammasome • ↓ NLRP3 (IHC/qPCR on conjunctiva) Indirect evidence (NF-κB) Inflammatory markers • ↓ TNF-α, IL-6, IL-1β, IFN-γ (qPCR on conjunctiva)	Park et al. (2019)
SM934 (M)	Rat/Mouse (SCOP/BAC)	0.1%, 0.5% (topical)	• ↑ Tear secretion (phenol red thread) • ↑ GCD (PAS) • ↓ Corneal FL scores • ↓ Corneal opacity scores • ↓ Corneal neovascularization scores • ↓ Conjunctival irritation scores (hyperemia/edema/secretion) • ↓ Rose bengal staining scores	TLR4/NF-κB↓, NLRP3↓	Direct evidence NLRP3 inflammasome • ↓ NLRP3 (WB/IHC/qPCR on conjunctiva) • ↓ ASC, cleaved Caspase-1 (WB on conjunctiva) • ↓ Caspase 1, IL 1β (qPCR on conjunctiva) TLR4/NF-κB pathway ↓TLR4, MyD88 (WB/IHC on conjunctiva) Immune cells • ↓ CD68+TLR4+ macrophages (IF on conjunctiva) • ↓ MPO + cells (IHC on conjunctiva) Inflammatory markers • ↓ TNF-α, IL-6, IL-10, MCP-1 (ELISA on conjunctiva)	Yang et al. (2021)
PS-GA-RGD nano (M)	Mouse (SCOP + dry environment)	5 mg/mL (topical)	• ↓ Corneal FL scores • ↓ Corneal epithelial apoptosis (TUNEL) • ↑ GCD (PAS)	NF-κB↓	Indirect evidence Inflammatory cytokines • ↓ IL-6, IL-1β (ELISA on cornea/conjunctiva)	Li (2023)
Gallic Acid (M)	Mouse (SCOP + dry environment)	5 mg/mL (topical)	• ↓ Corneal FL scores • ↓ Corneal epithelial cell apoptosis (TUNEL) • ↑ GCD (PAS)	NF-κB↓	Indirect evidence Inflammatory cytokines • ↓ IL-6, IL-1β (ELISA on cornea/conjunctiva)	Li et al. (2023)
Berberine (M)	Mouse (SCOP + DS)	0.5, 2 mg/mL (topical)	• ↑ Tear secretion (phenol red thread) • ↓ Corneal barrier dysfunction (OGD) • ↑ GCD (PAS) • ↓ Corneal/conjunctival epithelial apoptosis (TUNEL) • Improved corneal/conjunctival histology (HE)	PI3K/AKT/NF-κB ↓, MAPK↓	Direct evidence PI3K/AKT/NF-κB pathway • ↓ p-AKT, p-IKKα/β, p-NF-κB (WB on conjunctiva/cornea) MAPK pathway • ↓ p-p38, p-ERK1/2 (WB on conjunctiva/cornea) Apoptosis • ↓ cleaved Caspase-3, cleaved Caspase-8 (WB/IF on conjunctiva/cornea) MMPs • ↓ MMP-3, MMP-9 (WB/IF on conjunctiva/cornea) Immune cells • ↓ CD4^+^ T cells (IF on conjunctiva/cornea) Inflammatory markers • ↓ IL-1α, IL-1β, IL-6, TNF-α, IL-17, IFN-γ (qRT-PCR on conjunctiva)	Han et al. (2023a)
Yangyin Runmu Pills (F)	SD rat (bilateral orchiectomy)	9 g/100 mL (gavage)	• ↑ SIT, TBUT • ↑ GCD (HE) • Improved corneal/conjunctival histopathology (HE)	p38 MAPK↓	Direct evidence MAPK pathway • ↓ p38 (IHC on conjunctiva) • ↓ ICAM-1, p-p38 (WB on conjunctiva)	Li D. et al. (2015)
Yangyin Runmu Pills (F)	New Zealand rabbit (SCOP)	0.782, 0.869, 1.738 g/kg (gavage)	• ↑ Tear secretion (phenol red thread) • Improved LG histology (HE)	p38 MAPK↓	Direct evidence MAPK pathway • ↓ p38 (WB on LG) Apoptosis • ↓ Bax, ↑ Bcl-2 (WB/qPCR on LG)	Li et al. (2019)
Qingxuan Runmu Decoction (F)	SD rat (BAC)	0.6 g/mL (gavage)	• ↑ SIT • ↓ Corneal FL scores • Improved corneal morphology (HE)	TRAF6/TAK1↓	Direct evidence TRAF6/TAK1 pathway • ↓ TRAF6 (IHC/WB on cornea) • ↓ p-TAK1/TAK1 (WB on cornea) MMPs • ↓ MMP9 (ELISA on cornea) Inflammatory markers • ↓ IL-1β, TNF-α (ELISA on cornea)	Zhao et al. (2024a)
Qingxuan Runmu Decoction (F)	SD rat (BAC)	0.6 g/mL (gavage)	• ↑ SIT • ↓ Corneal FL scores • Improved corneal ultrastructure (TEM) • Improved conjunctival histology (HE) • ↑ GCD (HE)	TLR4/MyD88/NF-κB↓	Direct evidence TLR4/MyD88/NF-κB pathway • ↓ TLR4, MyD88 (RT-qPCR on cornea/conjunctiva/LG) • ↓ p-p65 (ELISA on cornea/conjunctiva/LG)	Zhao et al. (2024b)
Qingxuan Runmu Decoction (F)	Wistar rat (LG excision)	equivalent dose (gavage)	• ↑ Tear secretion (phenol red thread) • ↓ Corneal FL scores • ↑ GCD (PAS)	HMOX1/HIF-1↓	Direct evidence HMOX1/HIF-1 pathway • ↓ HMOX1, HIF-1α (WB on cornea) Ferroptosis • ↑ GPX4, ↓ TFRC, ACSL4 (qPCR on cornea) Oxidative stress • ↑ GSH, ↓ MDA, ROS, Fe2+ (Assay kits on cornea)	Wang J. et al. (2024)
Runmu Ling granules (F)	SD rat (SCOP)	0.75, 1.5 g/kg (gavage)	• ↑ Tear secretion (phenol red thread) • ↓ Corneal FL scores • ↓ Corneal epithelial cell damage (TUNEL) • Improved corneal histopathology (HE) • ↓ Corneal inflammatory cell infiltration (HE)	NLRP3/GSDMD↓	Direct evidence NLRP3 inflammasome • ↓ NLRP3, GSDMD ((IF/WB on cornea) • ↓ ASC, pro-Caspase-1, cleaved Caspase-1 (WB on cornea) Inflammatory markers • ↓ IL-1β, IL-18, TNF-α (WB on cornea)	Luo et al. (2024)
Runmu Ling Eye Drops (F)	New Zealand rabbit (BAC)	0.51 g/mL (topical)	• ↑ SIT, TBUT • ↓ Corneal FL scores • ↑ GCD (HE) • Improved corneal/conjunctival histopathology (HE) • ↓ Corneal/conjunctival inflammatory cell infiltration (HE)	JNK/P38↓	Direct evidence MAPK/NF-κB pathways • ↓ p-JNK/JNK, p-p38/p38, p-p65/p65 (WB on cornea) MMPs • ↓ MMP-9 (WB on cornea) Inflammatory markers • ↓ TNF-α (WB on cornea) • ↓TNF-α, IL-6 (ELISA on LG/aqueous humor) Barrier function • ↑ MUC5AC (WB on cornea, IF on conjunctiva)	Song et al. (2023)
Runmu Xiaoyao Powder (F)	C57BL/6J mouse (BAC + pain)	7.25, 14.5, 29 g/kg (gavage)	• ↑ Tear secretion (phenol red thread) • ↓ Corneal FL scores • Improved corneal/LG histology (HE) • ↓ LG cell apoptosis (TUNEL)	IRAK1/TRAF6/NF-κB↓	Direct evidence IRAK1/TRAF6/NF-κB pathway • ↓ IRAK1, TRAF6, p65 (WB/qPCR on cornea/LG) • ↓ p-p65 (WB on cornea/LG) MMPs • ↓ MMP-3, MMP-9 (WB on cornea) Inflammatory markers • ↓ IL-1β, TNF-α (IHC/IF on cornea/LG) miRNA regulation • ↑ miR-146a-5p (qPCR on cornea/LG)	Liu T. et al. (2024)
Modified Danzhi Xiaoyao Powder (F)	C57 mouse (BAC + pain)	6.24, 12.48, 24.96 g/kg (gavage)	• ↑ Tear secretion (phenol red thread) • ↑ TBUT • ↓ Corneal FL scores • Improved corneal/LG/liver histology (HE) • ↓ LG cell apoptosis (TUNEL) • ↓ Anxiety-like behavior (OFT, EPM)	fcγR-mediated phagocytosis↓	Functional evidence Phagocytosis-related proteins • ↓ CDC42, ARPC2, ACTR3 (IHC/qPCR on cornea, IHC on LG, ELISA on serum) Inflammatory markers • ↓ TNF-α, IL-1β (IHC/qPCR on cornea, IHC on LG, ELISA on serum) Immune function • ↓ Phagocytic function of macrophages (Flow cytometry)	Liu et al. (2024b)
Modified Siwei Dafa Powder (F)	SD rat (low-T and low-H chamber)	5.89 g/kg (gavage)	• ↑ SIT, TBUT • ↓ Corneal FL scores • Improved tongue coating (from dry to moist)	Bax/Caspase-9/Caspase-3↓	Direct evidence Apoptosis pathway • ↓ Bax, Caspase-9, ↑ Bcl-2 (WB on LG) • Caspase-3 trended downward (not significant) Inflammatory markers • ↓ IL-6 (ELISA on LG)	Chen L. et al. (2023)
Huashi Runjing Decoction (F)	SD rat (bilateral orchiectomy)	0.93 g/mL (gavage)	• ↑ SIT, TBUT • ↓ Corneal FL scores • Improved LG histology (HE)	NF-κB↓	Direct evidence NF-κB pathway • ↓ IKKα, IKKβ, IκB, p65 (IHC/WB on LG) Inflammatory markers • ↓ TNF-α, IL-1α, IL-6 (ELISA on serum) Hormone levels • ↑ Testosterone (Radioimmunoassay on serum)	Yang J. et al. (2023)
Xiaosheng Granules (F)	C57BL/6 mouse (SCOP)	3.6 mg/g (gavage)	• ↑ Tear secretion (phenol red thread) • ↑ TBUT • ↓ Corneal FL scores • Improved corneal and LG morphology (HE) • ↓ Corneal epithelial cell apoptosis (TUNEL)	PERK-elF2α-ATF4-Chop/Caspase-12, MAPKs/NF-kB↓	Indirect evidence Inflammatory markers • ↓ IL-1β, IL-6, TNF-α (IHC on cornea)	Du (2018)
Erzhi Pills (F)	C57BL/6 mouse (SCOP)	12 g/kg (gavage)	• ↑ Tear secretion (phenol red thread) • ↓ Corneal FL scores	NF-κB↓	Direct evidence NF-κB pathway • ↓ p-p65 (WB on LG) Inflammatory markers • ↓ TNF-α, IL-1β (qPCR/CBA on cornea/conjunctiva) • ↓ IL-4 (qPCR on cornea/conjunctiva)	Shi X. et al. (2023)
Zhenshi Guben Liquid (F)	BALB/c mouse (SCOP + dry environment)	25.96, 51.92, 103.84 g/kg (gavage)	• ↑ Tear secretion (phenol red thread) • ↑ TBUT • ↓ Corneal FL scores • Improved corneal and LG histology (HE) • ↓ Alleviated corneal and LG ultrastructural damage (TEM)	IKK/IkB/NF-kB↓	Direct evidence NF-κB pathway • ↓ p-IKK, p-IκBα, p-p65 (WB on cornea/LG) • ↓ IKK, p65, ↑ IκBα (qPCR on cornea/LG) Inflammatory markers • ↓ IL-1β, IL-6, MMP-9, Caspase-3 (ELISA on cornea/LG) • ↓ TNF-α (WB/qPCR/ELISA on cornea/LG)	Dong (2024)

^a^ Detailed botanical/chemical information for (M) and (E) interventions is provided in Supplementary Table S2; for (F) formulas in Supplementary Table S3.

^b^ Dose (Route): Key effective dose(s) and route shown. Full regimens in Supplementary Table S4.

^c^ Primary Outcomes: ↑ indicates improvement/increase; ↓ indicates reduction/decrease.

^d^ *In vivo* Validation (Evidence Quality): Categories defined in Table 1 footnote c.

**TABLE 3:** *In vitro* mechanistic evidence for TCM interventions in DED cell models.

TCM intervention (Type)^a^	Cell models/Stressor	Dose^b^	Key pathway modulation	Evidence quality^c^	Ref.
Quercetin (M)	HCECs/Hyperosmolarity	100 µM	PTEN↑, PI3K/AKT↓	Direct evidence PI3K/AKT pathway • ↑ PTEN, ↓ p-PI3dK, p-AKT (WB/IF)sup Inflammatory markers • ↓ IL-6, TNF-α (PCR/ELISA)	Huang et al. (2024)
Kaempferol (M)	HCECs/Hyperosmolarity	80 µM	p38 MAPK↓	Direct evidence MAPK pathway • ↓ p38 (WB/qPCR) Inflammatory markers • ↓ TNF-α, IL-6 (WB/qPCR) Apoptosis • ↓ Apoptosis (TUNEL) Proliferation • ↑ Ki67 (IHC)	Li D. (2021)
Curcumin (M)	HCECs/Hyperosmolarity	5 µM	MAPKs↓, NF-κB↓	Direct evidence MAPK pathway • ↓ p-p38, p-JNK (ELISA) NF-κB pathway • ↓ nuclear p65 (ELISA) Inflammatory markers • ↓ IL-1β, IL-6, TNF-α (ELISA) • ↓ IL-1β (RT-PCR)	Chen et al. (2010)
PCE Aqueous Extract (E)	HCECs/Hyperosmolarity	1, 10, 100 µg/mL	NF-κB↓	Direct evidence NF-κB pathway • ↓ p-p65, COX-2 (WB) Inflammatory markers • ↓ TNF-α, IL-6 (qPCR) Barrier function • ↑ MUC4 (qPCR)	Park et al. (2018)
Oridonin (M)	HCE-T cells/Hyperosmolarity	2, 4 µM	NLRP3/Caspase-1/GSDMD↓	Direct evidence NLRP3 inflammasome • ↓ NLRP3, Caspase-1, N-GSDMD (WB) • ↓ NLRP3, Caspase-1 (qPCR) • ↓ IL-1β (qPCR/ELISA)	Li X. et al. (2024)
Pterostilbene nanocomposite (PS-CG) (M)	HCECs/Hyperosmolarity	2.5, 5, 10, 20 µg/mL	Keap1/Nrf2/ARE↑	Direct evidence Nrf2 pathway • ↑ Nrf2 (WB/qPCR) • ↑ Keap1 (WB) Antioxidant enzymes • ↑ SOD1, CAT (WB/qPCR) • ↑ GPX (qPCR) Oxidative stress • ↓ ROS (Flow cytometry) Cytotoxicity • ↓ LDH (Biochemical assay) Apoptosis • ↓ Apoptosis (Flow cytometry)	Lin et al. (2022)
Esculetin (M)	HCECs/H_2_O_2_	20, 40, 80 µM	Nrf2↑	Direct evidence Nrf2 pathway • ↑ nuclear Nrf2 (WB) • ↑ Nrf2 (qPCR) Apoptosis • ↓ Bax, cleaved Caspase-3, ↑ Bcl-2 (WB) • ↓ Apoptosis (Flow cytometry) Antioxidant enzymes • ↑ HO-1, NQO1, GCLM, SOD1, SOD2 (qPCR) Oxidative stress • ↑ SOD activity (Enzyme assay) • ↓ ROS (Flow cytometry)	Zhang et al. (2021a)
Acteoside (M)	HCECs/H_2_O_2_	160 µM	Nrf2↓	Direct evidence Nrf2 pathway • ↓ Nrf2 (WB/qPCR) Antioxidant enzymes • ↓ HO-1, NQO1, COX-2 (WB/qPCR)	Huang and Peng (2024)
Astaxanthin (M)	HCECs/Hyperosmolarity	5, 10 µM	Keap1-Nrf2/HO-1↑	Direct evidence Nrf2 pathway • ↓ Keap1 (WB/qPCR) • ↑ Nrf2 (IF/qPCR) Antioxidant enzymes • ↑ HO-1 (WB/IF/qPCR) • ↑ SOD1, SOD2, CAT (qPCR) Oxidative stress • ↑ SOD, CAT activity (Enzyme assay) • ↓ ROS (ROS assay)	Liu Z. et al. (2025)
Puerarin (M)	HCE-2 cells/Hyperosmolarity	10, 30, 50 µM	SIRT1↑, NLRP3↓	Direct evidence SIRT1/NLRP3 pathway • ↑ SIRT1, ↓ NLRP3 (WB) Apoptosis • ↑ Bcl-2, ↓ Bax, cleaved Caspase-3 (WB) • ↓ Apoptosis (Flow cytometry) Barrier function • ↑ ZO-1, occludin (IF/WB) Inflammatory markers • ↓ TNF-α, IL-1β, IL-6 (ELISA) Oxidative stress • ↑ CAT, SOD, GSH/GSSG ratio (Enzyme assay) • ↓ ROS (ROS assay)	Dong et al. (2024)
Oroxylin A (M)	HCECs/Hyperosmolarity	8, 10 µg/mL	SIRT3-SOD2/HIF-1α↑	Direct evidence SIRT3 pathway • ↑ SIRT3 (WB/qPCR) NLRP3 inflammasome • ↓ NLRP3, Caspase-1 (WB/qPCR) • ↓ N-GSDMD, cleaved Caspase-1 (WB) • ↓ GSDMD (qPCR) Antioxidant enzymes • ↑ SOD2, ↓ HIF-1α (WB) Apoptosis • ↑ Bcl-2, ↓ Bax, Caspase-3, cleaved Caspase-3 (WB) Inflammatory markers • ↓ IL-6, IL-1β, TNF-α (qPCR) Oxidative stress • ↓ ROS (ROS assay) • ↓ NO (NO assay) Cytotoxicity • ↓ LDH (Enzyme assay)	Liu X. et al. (2025)
Salidroside (M)	HCECs/Hyperosmolarity	25, 50, 100 µM	AMPK/SIRT1↑	Direct evidence AMPK/SIRT1 pathway • ↑ p-AMPK, SIRT1 (WB) Autophagy • ↑LC3II, ↓ SQSTM1 (WB) • ↑ Nrf2 nuclear translocation (IF) Nrf2 pathway • ↑ nuclear Nrf2 (WB) Antioxidant enzymes • ↑ HO-1, NQO1 (WB) Inflammatory markers • ↓ TNF-α, IL-1β, IL-6 (ELISA) Apoptosis • ↓ Apoptosis (Flow cytometry) Oxidative stress • ↓ ROS (ROS assay) • ↑ SOD, CAT activity, ↓ MDA (Enzyme assay)	Liang et al. (2023)
Buddleia flavonoids (E)	LGECs/H_2_O_2_	8.95 × 10^−2^ mol/L medicated plasma	STAT1↑	Direct evidence STAT1 pathway • ↑ p-STAT1 (WB)	Wang et al. (2010)
Resveratrol (M)	HCE-T cells/Hyperosmolarity	25 µM	Notch↑	Direct evidence Notch pathway • ↑ Notch2, Notch4, Dll3, Jagged1, Hes1 (WB/qPCR) • ↑ Hes5, Hey1 (qPCR) Apoptosis • ↓ Bax (WB/qPCR/IF) • ↑ Bcl-2 (qPCR/IF) Vitamin D pathway • ↑ VDR (WB/qPCR/IF) • ↑ 25-hydroxyvitamin D (ELISA) Antioxidant enzymes • ↓ HO-1, ↑ SOD2 (qPCR) Oxidative stress • ↓ ROS (ROS assay)	Shetty et al. (2020)
Evodiamine (M)	HCECs/Hyperosmolarity	0.1 µM	p53/mTOR↑	Direct evidence p53/mTOR pathway • ↑ nuclear p-p53, ↓ cytoplasmic p-p53, ↓ p-mTOR (WB) • ↑ p53 nuclear translocation (IF) Autophagy • ↑ LC3II, ↓ p62 (WB) • ↑ Autophagic flux (Fluorescence Microscopy) Inflammatory markers • ↓TNF-α, IL-6, IL-1β (WB/qPCR) Apoptosis • ↓ Apoptosis (Flow cytometry) Oxidative stress • ↓ ROS (Flow cytometry) • ↑ SOD, CAT activity, ↓ MDA (Enzyme assay)	Li et al. (2025)
Astragaloside IV (M)	HCECs/BAC	5, 10 µM	MUC1/EGFR↑	Direct evidence MUC1/EGFR pathway • ↑ MUC1 (WB/qPCR/ELISA) • ↑ EGFR (WB/qPCR)	Chu et al. (2021)
Dendrobium water extracts (E)	HKs/Hyperosmolarity	250, 500 µg/mL	ERK↓	Direct evidence MAPK pathway • ↓ p-ERK (WB) MMPs • ↓ MMP-9 (WB) Barrier function • ↓ AQP1, AQP4, AQP5 (qRT-PCR)	Ling et al. (2022)
Linarine (M)	HCECs/Hyperosmolarity	15 µM	MAPK↓, NF-κB↓	Indirect evidence Inflammatory markers • ↓TNF-α, IL-1β (ELISA)	Liu et al. (2024a)
Paeoniflorin (M)	HCECs/Hyperosmolarity	0.01%, 0.1%, 1%	MAPKs↓, NF-κB↓	Direct evidence MAPK pathway • ↓ p-p38, p-JNK, p-ERK (WB) NF-κB pathway • ↓ NF-κB (WB/RT-PCR) Inflammatory markers • ↓ TNF-α (WB/RT-PCR) • ↓ IL-1, IL-6 (WB/RT-PCR/IF)	Zhao et al. (2019)
Lutein (M)	HCECs/Hyperosmolarity	1, 3, 10 µM	p38 MAPK↓, JNK1/2↓, NF-κB↓	Direct evidence MAPK pathway • ↓ p-p38, p-JNK1/2 (ELISA) NF-κB pathway • ↓ NF-κB (ELISA) Inflammatory markers • ↓ IL-6 (ELISA)	Chao et al. (2016)
KIOM-2015EW (E)	HCECs/Hyperosmolarity	0.05, 0.1, 0.2 mg/mL	MAPKs↓, NF-κB↓	Direct evidence NF-κB pathway • ↓ p65 nuclear translocation, p-IκB-α (IF/WB) MAPK pathway • ↓ p-p38, p-ERK, p-JNK (WB) Apoptosis • ↓ cleaved Caspase-3, cleaved PARP, ↑ Bcl-2 (WB) Inflammatory markers • ↓ TNF-α, IL-1β, IL-6 (ELISA/IF/RT-PCR)	Kim et al. (2018)
Polydatin (M)	Human conjunctival cells/Hyperosmolarity	0.1, 1, 10 µM	NF-κB↓, NLRP3↓	Direct evidence NF-κB pathway • ↓ p-p65, nuclear p65, COX-2 (WB) NLRP3 inflammasome • ↓ NLRP3, cleaved Caspase-1 (WB) Antioxidant enzymes • ↑ HO-1, GPX, SOD1 (WB) Inflammatory markers • ↓ TNF-α, IL-6, IL-1β, MMP9 (qPCR) Oxidative stress • ↓ ROS (ROS assay)	Park et al. (2019)
SM934 (M)	RAW 264.7/LPS	10 µM	TLR4/NF-κB↓, NLRP3↓	Direct evidence TLR4/NF-κB pathway • ↓ TLR4, MyD88, p-NF-κB (WB) NLRP3 inflammasome • ↓ NLRP3, ASC, cleaved caspase-1 (WB) Inflammatory markers • ↓ TNF-α, IL-6, IL-1β (ELISA)	Yang et al. (2021)
PS-GA-RGD nano (M)	RAW 264.7/LPS	10 µM	NF-κB↓, Nrf2↑	Direct evidence NF-κB pathway • ↓ p-p65, p-IκBα (WB) Nrf2 pathway • ↑ p-Nrf2, HO-1 (WB) Inflammatory markers • ↓ TNF-α, IL-6 (ELISA/qRT-PCR) • ↓ NO (ELISA) Oxidative stress • ↓ ROS (Flow cytometry/IF) • ↓ 8-OHdG (IF)	Li (2023)
Gallic Acid (M)	HCECs/Hyperosmolarity; RAW 264.7/LPS	100 µM	NF-κB↓, Nrf2↑	Direct evidence NF-κB pathway • ↓ p-p65, p-IκBα (WB) Nrf2 pathway • ↑ p-Nrf2, HO-1, NQO1 (WB) • ↑ Nrf2 nuclear translocation (IF) Inflammatory markers • ↓ TNF-α, IL-6, NO (ELISA) Oxidative stress • ↓ ROS (Flow cytometry/IF)	Li et al. (2023)
Berberine (M)	HCECs/Hyperosmolarity	2.5, 5 µM	NF-κB↓	Direct evidence NF-κB pathway • ↓ NF-κB nuclear translocation (IF) Cell viability • ↑ Cell viability (Cell Viability Assay)	Han et al. (2023a)
Genistein (M)	iHCECs, priHCECs/Hyperosmolarity	50 µmol/L	ROS/NLRP3/IL-1β↓, Nrf2↑	Direct evidence NLRP3 inflammasome • ↓ NLRP3, Caspase-1 (RT-PCR) Nrf2 pathway • ↑ nuclear Nrf2 (WB) Inflammatory markers • ↓ IL-1β (ELISA/RT-PCR) • ↓ IL-18 (RT-PCR) Oxidative stress • ↓ ROS, 8-OHdG (IF/Fluorescence Assay) • ↑ SOD, Catalase, GR activity (Enzyme Activity Assay) Cell viability • ↑ Cell viability (Cell Viability Assay)	Zhou et al. (2020)
Yangyin Runmu Pills (F)	Primary rabbit LG cells/TNF-α	Medicated serum	TNF-α/p38 MAPK/p53↓	Direct evidence MAPK pathway • ↓ p38 (WB) Inflammatory markers • ↓ TNF-α (WB) • ↓ IL-1β, IL-6 (ELISA) Apoptosis • ↓ p53 (WB)	Li D. et al. (2022)
Qingxuan Runmu Decoction (QXRMY) (F)	HCE-2 cells/Hyperosmolarity	2.5%, 5%, 10% medicated serum	HMOX1/HIF-1↓	Direct evidence HMOX1/HIF-1 pathway • ↓ HMOX1, HIF-1α (WB) Ferroptosis • ↑ GPX4, ↓ TFRC, ACSL4 (qPCR) • ↓ Fe2+ (Assay kits) Apoptosis • ↓ Apoptosis (Flow cytometry) Oxidative stress • ↓ MDA, ROS, ↑ GSH (Assay kits)	Wang J. et al. (2024)
Qishen prescription (F)	HCECs/Hyperosmolarity	15% medicated serum	MAPKs↓	Direct evidence MAPK pathway • ↓ p-ERK1, p-p38 (WB) Inflammatory markers • ↓ IL-1β, IL-8 (ELISA) Apoptosis • ↓ Caspase-3 (ICC) Cell viability • ↑ Cell viability (CCK-8)	Zhao et al. (2022b)
Qishen prescription (F)	HCECs/Hyperosmolarity	15% medicated serum	JNK1/AQP5↓	Direct evidence JNK1/AQP5 pathway • ↓ p-JNK1 (WB) • ↓ AQP5 (ICC/WB) Inflammatory markers • ↓ TNF-α, IL-6 (ELISA) Apoptosis • ↓ Caspase-1 (ICC) Cell viability • ↑ Cell viability (CCK-8)	Zhao et al. (2022a)
Runmu Ling granules (F)	HCECs/BAC	2.5%, 5%, 10% medicated serum	NLRP3/GSDMD↓	Direct evidence NLRP3 inflammasome • ↓ NLRP3, GSDMD, ASC, pro-Caspase-1, cleaved Caspase-1 (WB) Inflammatory markers • ↓ IL-1β, IL-18, TNF-α (WB)	Luo et al. (2024)
Modified Danzhi Xiaoyao Powder (F)	HCECs/Hyperosmolarity; RAW 264.7, THP-1/LPS	N/S	Not assessed	Indirect evidence Inflammatory markers • ↓ TNF-α, IL-1β (ELISA)	Liu et al. (2024b)
Sihuang Qingling Liquid (F)	RAW 264.7/LPS	10, 20 µg/mL	MAPKs↓, NF-κB↓	Direct evidence MAPK pathway • ↓ p-p38, p-JNK, p-ERK (WB) NF-κB pathway • ↓ p-IκBα, p-p65 (WB) • ↓ NF-κB nuclear translocation (IF) Inflammatory markers • ↓ TNF-α, IL-6 (ELISA) Oxidative stress • ↓ ROS (Flow cytometry/LSCM)	Chen et al. (2024)
Zhenzhu Mingmu Eye Drops (F)	HCECs/TNF-α+IFN-γ	50×, 100×, 1,000× dilution	Caspase-8/Caspase-3↓	Direct evidence Apoptosis pathway • ↓ cleaved Caspase-8, cleaved Caspase-3, cleaved PARP (WB) • ↑ Bcl-2, ↓ Bax (WB) • ↓ Apoptosis, G2 phase arrest (Flow cytometry) MAPK pathway • ↓ p-ERK1/2 (WB) Cell viability • ↑ Cell viability (MTT)	Wu et al. (2022)
Xiaosheng Granules (F)	HCEC, RAW 264.7/Hyperosmolarity or Cytokines (TNF-α+IFN-γ)	100, 400 µg/mL	PERK-eIF2α-ATF4-CHOP/Caspase-12↓, MAPK↓, NF-κB↓	Direct evidence (HCECs) ER stress pathway • ↓ p-PERK, p-eIF2α, ATF4, CHOP, cleaved Caspase-12 (WB) • ↓ Ca2+ (Flow cytometry) Apoptosis • ↓ cleaved Caspase-9, cleaved Caspase-3, ↑ Bcl-2, ↓ Bax (WB) • ↓ Apoptosis, mitochondrial membrane potential (Flow cytometry) Cytotoxicity • ↓ LDH (LDH assay) Oxidative stress • ↓ ROS (Flow cytometry) Cell viability • ↑ Cell viability (MTT) Direct evidence (RAW264.7) MAPK/NF-κB pathway • ↓ p-JNK, p-ERK, p-IKKβ, p-p65, ↑ IκBα (WB) Inflammatory markers • ↓ TNF-α, IL-6, IL-1β (ELISA/qPCR) Oxidative stress • ↓ ROS (Flow cytometry) ER stress • ↓ Ca2+ (Flow cytometry)	Du (2018)

^a^ Detailed botanical/chemical information for (M) and (E) interventions in Supplementary Table S2; for (F) formulas in Supplementary Table S3.

^b^ Dose: Key effective concentration(s) shown. Full concentration ranges in Supplementary Table S4.

^c^ Evidence quality: Categories defined in Table 1 footnote c.

#### 4.2.1 Inclusion criteria

- Study Design: *In vitro* or *in vivo* experimental studies.
- Population/Subject: Models of DED.
- Intervention: Treatment with TCM metabolites, extracts, or formulas.
- Outcomes: Investigation of the mechanisms involving specific signaling pathways.

#### 4.2.2 Exclusion criteria

- Publication Type: Reviews, conference papers, or case reports.
- Disease Focus: Studies not primarily focused on DED (e.g., those exclusively on Sjögren’s syndrome).
- Intervention: Interventions not involving TCM or its derivatives.
- Mechanistic Focus: Purely computational studies without experimental validation of specific pathways.
- Accessibility: Articles for which the full text could not be retrieved.
- Data Uniqueness: Duplicate publications or studies with overlapping datasets.

## 5 Plant-derived metabolites and botanical drug extracts in TCM and related signaling pathways

### 5.1 Classical signaling pathways

**FIGURE 2:**
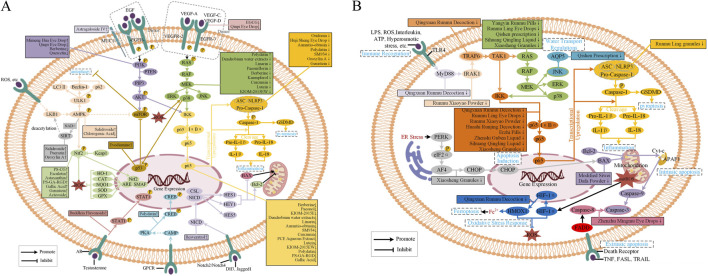
Signaling pathways modulated by TCM in DED. **(A)** Pathways regulated by pure metabolites and standardized botanical drug extracts/formulations. **(B)** Pathways regulated by multi-herb TCM formula.

The onset and progression of DED is associated with a pathological cascade of ocular surface signaling pathways. Abnormal activation or inhibition of key signaling pathways establishes a vicious feedback loop characterized by “tear film instability-tear hyperosmolarity-inflammation-apoptosis,” thereby exacerbating the disease process. Consequently, targeting key elements of these signaling pathways has emerged as a strategy of significant interest for DED management. This article comprehensively describes the mechanisms by which TCM modulate this pathological cycle by regulating several important signaling targets and offers a solid theoretical foundation for the utility of natural medicines in DED therapy. The signaling pathways modulated by pure metabolites and standardized botanical drug extracts/formulations are summarized in Figure 2A, which integrates the key pathway components and crosstalk supported by direct experimental evidence detailed in the following tables. Experimental findings are presented in a structured format. Table 1 provides a synthesized overview of the evidence strength for each signaling pathway. Detailed study data are separated into Table 2 (*in vivo* animal model studies) and Table 3 (*in vitro* cellular model studies). The pathway-specific experimental results aggregated to generate Table 1 are detailed in Supplementary Table S1. Botanical and chemical definitions for each intervention are provided in Supplementary Table S2, and a full registry of experimental doses and parameters is available in Supplementary Table S4.

#### 5.1.1 PI3K/AKT signaling pathway

Phosphatidylinositol 3-kinase (PI3K)/protein kinase B (PKB/AKT) pathway is involved in modulating key cellular activities such as growth, metabolism, apoptosis, and inflammatory modulation. Its core cascade involves PI3K/AKT/mechanistic target of rapamycin (mTOR), which is inhibited by phosphatase and tensin homolog (PTEN) (Wang J. et al., 2022). When extracellular membrane ligands (e.g., interleukins, IFNs, tumor necrosis factors, chemokines, growth factors) bind to cell membrane receptors (e.g., receptor tyrosine kinase (RTK), G protein-coupled receptor (GPCR)), PI3K catalyzes the conversion of PIP2 to PIP3, thus triggering activation of phosphoinositide-dependent protein kinase 1 (PDK1) and phosphorylation of AKT. Phosphorylated AKT regulates cell survival, inflammatory response, and immune homeostasis through downstream molecules such as mTOR, NF-κB, forkhead box proteins (Fox) O1, and Caspase-9 (Wang M. et al., 2022). In DED, excessive accumulation of ROS induced by hyperosmolarity and other extreme conditions enhance keratocyte apoptosis and ocular inflammation by suppressing PI3K/AKT signaling activation. Moderate activation of PI3K/AKT suppresses apoptosis and oxidative stress in the corneal epithelial cells, but over-activation exacerbates inflammation via NF-κB (Chen K. et al., 2022). Therefore precise regulation of this pathway is essential.

Quercetin, a flavonoid isolated from various plants, fruits, and vegetables, including the TCM *Apocynum venetum* L. [Apocynaceae; apocyni veneti folium] (dogbane leaf), exhibits anti-inflammatory, antioxidant, and immunoregulatory activities (Di Petrillo et al., 2022). Experimental investigations show that quercetin mitigates LFU inflammation, alleviates corneal injury in DED mice, and increases tear secretion and goblet cell density (Oh et al., 2015). In human corneal epithelial cells (HCECs), 100 μM quercetin attenuates hyperosmolarity-induced upregulation of IL-6 and TNF-α by modulating the PTEN/PI3K/AKT pathway (decreasing p-PI3K and p-AKT while elevating PTEN); the effects of quercetin were comparable to that of the PI3K inhibitor LY294002 (Huang et al., 2024).

*Fraxinus chinensis subsp. rhynchophylla* (Hance) A.E.Murray [Oleaceae; fraxini cortex] (Chinese ash) is associated with pharmacological activities such as dispelling wind, improving vision, clearing heat, and resolving toxins, and contains various active metabolites s such as coumarins, flavonoids, and triterpenoids; coumarins exhibit anti-inflammatory and anti-oxidant properties (Zheng Z. et al., 2024). PI3K/AKT pathway upregulates retinoid-related orphan receptor γt (RORγt), a pivotal regulator of Th17 differentiation, and suppresses Foxp3, a critical regulator of Treg differentiation, by activating mTORC1 (Wang J. et al., 2022). Qinpi eye drops improve lacrimal gland atrophy and lymphocyte infiltration and regulate Th17/Treg balance in the autoimmune DED model rabbits by decreasing Akt and ROR-γt and increasing Foxp3 levels (Jiang et al., 2023).

*Buddleja officinalis* Maxim. [Scrophulariaceae; buddlejae flos] (pale butterflybush flower) is rich in flavonoids, phenylethanols, terpenoids, alkaloids, and volatile oils, and exhibits antioxidative, immunomodulatory, and neuroprotective properties (Chen X. et al., 2022). In desiccated DED model rabbits, Mimeng Hua eye drops (1.0, 1.5, and 3.0 mg/mL) repaired lacrimal gland structure, upregulated PI3K and Akt expression, and inhibited Caspase-9 activation. This suggested that PI3K/AKT mediated anti-apoptotic effects. Optimal therapeutic outcomes were observed at 3.0 mg/mL (Qin et al., 2019). Subsequent research should also delineate the individual contributions of specific metabolites within this complex extract.

#### 5.1.2 MAPK signaling pathway

MAPK cascade is a serine-threonine kinase system mediating extracellular-to-nuclear signal transmission (Cui L. et al., 2024). It regulates cell proliferation, differentiation, growth, apoptosis, inflammatory responses, and innate immunity (Kim and Choi, 2015). MAPK pathway encompasses four principal branches: extracellular signal-regulated kinases (ERK1/2), JNK, p38, and ERK5. When extracellular signals trigger membrane receptors, MAPK cascade primarily transmits signals via sequential phosphorylation by three kinases: MAPK kinase kinase (MAPKKK), MAPK kinase (MAPKK), and MAPK (Park and Baek, 2022). ERK1/2 activation is primarily driven by growth factors; ERK5 is induced by mitogens and oxidative stress; JNK and p38 respond to inflammatory cytokines and cellular stressors such as hyperosmolarity and oxidative damage (Kim and Choi, 2015). In DED, activation of the p38 MAPK pathway is understood to be a key driver of ocular surface damage. It not only promotes inflammatory responses and compromises barrier integrity but also directly contributes to limbal stem cell dysfunction and abnormal corneal epithelial differentiation, which are critical mechanisms underlying severe disease progression (Lin et al., 2023). Furthermore, ERK pathway stimulation enhances corneal epithelial cell proliferation, migration, and differentiation, thereby facilitating corneal repair and preserving corneal barrier function under physiological conditions. However, these processes may lead to destruction of the corneal epithelial barrier under certain pathological conditions (Sanie-Jahromi et al., 2025). Therefore, metabolites of TCM targeting the MAPK pathway show immense therapeutic potential.

Kaempferol, a flavonol present in the *B. officinalis* Maxim. [Scrophulariaceae; buddlejae flos] (pale butterflybush flower) (Chen et al., 2017), exhibits significant anti-inflammatory activity (Devi et al., 2015). At 80 μM concentration, kaempferol effectively reverses hyperosmolarity-induced HCEC apoptosis, decreases TNF-α and IL-6 expression, and inhibits p38 activation. This suggests that kaempferol maintains corneal epithelial integrity by blocking the p38 MAPK pathway (Li D., 2021).

Polydatin, a single-crystal metabolite derived from *Reynoutria japonica* Houtt. [Polygonaceae; polygoni cuspidati rhizoma et radix] (giant knotweed rhizome), possesses anti-inflammatory, antioxidant, and anti-apoptotic properties (Sajad et al., 2021). Polydatin attenuates corneal and conjunctival damage in the lacrimal gland models by reducing oxidative stress and inflammation (Park et al., 2019). Treatment of scopolamine (SCOP)-induced DED rats with polydatin eye drops (0.05% and 0.5%) significantly improves tear production and tear film stability and promote healing of corneal injury by activating the MEK/ERK pathway (upregulating MEK1/2 and ERK1/2 phosphorylation) and suppressing IL-1β, TNF-α, and IFN-γ expression. Conversely, MEK inhibitor U0126 reverses this effect (Cao et al., 2024).

Curcumin is obtained from the rhizome of the TCM *Curcuma longa* L. [Zingiberaceae; curcumae longae rhizoma] (turmeric) and exhibits anti-inflammatory properties (Kathryn et al., 2017; Raghavendhar R and Devanand L, 2019). Experimental data showed that 5 μM curcumin inhibits hyperosmolarity-induced p38 phosphorylation and p65 activation, as well as JNK phosphorylation, and the p38 inhibitor SB203580 could block hyperosmolar IL-1β generation, indicating that its anti-inflammatory action is contingent upon the p38 pathway (Chen et al., 2010).

#### 5.1.3 NF-κB signaling pathway

NF-κB signaling pathway plays a critical role in inflammation, apoptosis, and multiple autoimmune diseases (Barnabei et al., 2021). NF-κB pathway involves key subunits—including NF-κB1 (p105/p50), NF-κB2 (p100/p52), p65 (RELA), RELB, and c-REL. In unstimulated cells, NF-κB remains inactive within the cytoplasm in a complex with the inhibitor of κB (IκB) protein. NF-κB signaling is triggered via two distinct mechanisms—the canonical and alternative pathways. The canonical pathway responds to inflammatory cytokines, oxidative stress, and other stimuli, and involves the IKK complex (comprising IKKα, IKKβ, and NEMO). This cascade leads to the phosphorylation and subsequent degradation of IκB, thereby releasing p50, p65, and c-REL subunits, which then migrate into the nucleus and modulate the expression of specific genes (Guo et al., 2024). The alternative pathway is initiated by NIK kinase, which activates IKKα to process p100 into the p52 subunit, which then forms a heterodimer with RelB (RelB/p52) and translocates to the nucleus (Sun, 2017). NF-κB exhibits a bidirectional regulatory effect on apoptosis (Choi et al., 2019). It induces anti-apoptotic genes (e.g., Bcl-2, c-FLIP) to inhibit activation of Caspase-8 (Gao et al., 2023; Hayden and Ghosh, 2014) or exerts anti-apoptotic effects by inhibiting JNK signal transduction (Nakano, 2004); on the other hand, NF-κB also promotes apoptosis by upregulating death receptor CD95 (Fas) and pro-apoptotic gene Bax (Brint et al., 2013). During inflammation, cytokines such as TNF-α and IL-6 are triggered by NF-κB activation and can amplify their own synthesis, thereby fueling a self-perpetuating cycle of chronic inflammation (Taniguchi and Karin, 2018). Furthermore, NF-κB disrupts the corneal epithelial barrier and induces neovascularization through upregulation of MMPs (Pradhan et al., 2015; Zhang Y. et al., 2022). These observations highlight the important role of NF-κB in DED. Therefore, NF-κB is a promising focus for therapeutic intervention.

Berberine, an alkaloid from *Coptis chinensis* Franch. [Ranunculaceae; coptidis rhizoma] (Chinese goldthread), demonstrates potent anti-inflammatory properties (Och et al., 2020). In a mouse model of DED triggered by 0.2% SCOP injections and exposure to a dry environment, application of berberine eye drops (0.5, 1, and 2 mg/mL) leads to dose-dependent suppression of NF-κB pathway protein expression (NF-κB, IKK, and IκB). The most potent effect was observed with 2 mg/mL berberine. Furthermore, berberine also significantly reduced inflammatory cytokines (IL-1, IL-6, IL-17A, TNF-α, and IFN-γ) and CD4^+^ T cell infiltration. Moreover, it downregulated MMP-3/MMP-9 and Caspase-8, reduced corneal and conjunctival apoptosis, increased tear secretion, and protected the corneal epithelial barrier (Li, 2020).

Paeonol, the primary bioactive metabolite found in the TCM *Paeonia × suffruticosa* Andrews [Paeoniaceae; Moutan cortex] (tree peony bark), is a small phenolic molecule with anti-inflammatory, anti-apoptotic, antioxidant, and immunomodulatory properties (Wang et al., 2023; Zhang et al., 2019). Topical application of 5% paeonol eye drops inhibited NF-κB phosphorylation, downregulated MMP-3/MMP-9 and Caspase-8/Caspase-3 activities, suppressed apoptosis and CD4^+^ T cell infiltration, modulated Th1/Th2 balance, decreased IFN-γ and IL-17A levels and increased IL-13 levels. This enhanced tear production and corneal injury, reduced conjunctival goblet cell loss, and ameliorated DED ocular surface damage (Wu, 2019).

*Reynoutria japonica* Houtt. [Polygonaceae; polygoni cuspidati rhizoma et radix] (giant knotweed rhizome) and its extracts demonstrate anti-inflammatory and antioxidant effects (Ke et al., 2023). Po stoperative oral administration of its aqueous extract (10, 100, and 250 mg/kg) to rats with lacrimal gland excision-induced DED improved tear secretion, alleviated corneal irregularities, significantly decreased IL-6 and TNF-α levels, upregulated MUC4 levels, and inhibited MMP9 in a dose-dependent manner. In the *in vitro* experiments, PCE aqueous extract (1, 10, and 100 μg/mL) significantly restored MUC4 expression in hyperosmotic stress-induced HCECs, blocked p65 nuclear translocation and downregulated the activities of BAX, Caspase-3, and poly (ADP-ribose) polymerase (PARP), while enhancing Bcl-2 and antioxidant gene (HO-1/SOD1/GPX) expression levels in a concentration-dependent manner. This suggested that the PCE aqueous extract inhibits NF-κB signaling pathway, intrinsic apoptosis, and oxidative stress (Park et al., 2018).

KIOM-2015E, a hot water extract (KIOM-2015EW) or 25% ethanol extract (KIOM-2015EE) from the *Acer palmatum* Thunb. [Sapindaceae; Not recorded in pharmacopeia] (Japanese maple) leaves, possesses anti-inflammatory and antioxidant bioactivities (Bi et al., 2016). In benzalkonium chloride (BAC)-induced DED mice, treatment groups received 0.2% BAC, PBS solvent, topical KIOM-2015EW (1 mg/mL), topical KIOM-2015EE (0.5 or 1 mg/mL), oral KIOM-2015EE (100 mg/kg), or positive controls (CsA/FML eye drops). KIOM-2015E suppressed p65 nuclear translocation and reduced TNF-α, IL-1β, and IL-6 concentrations, markedly enhanced tear secretion, and attenuated corneal damage and apoptosis. Topical application produced greater beneficial effects than oral delivery, and KIOM-2015EE was more potent than KIOM-2015EW (Yim et al., 2022).

#### 5.1.4 NLRP3 signaling pathway

NLRP3 signaling pathway critically modulates inflammatory responses in innate immunity, featuring three key elements: NLRP3 receptor, apoptosis-associated speck-like protein (ASC), and pro-Caspase-1 (Fu and Wu, 2023). NLRP3 pathway activation occurs through a “dual-signaling” mode: the TLR/NF-κB pathway is part of the initiating signal and enhances the synthesis of NLRP3, pro-IL-1β, and pro-IL-18; subsequent activation triggers such as ATP and ROS lead to assembly of NLRP3 into oligomers; the oligomerized NLRP3 recruits and activates Caspase-1 via ASC, catalyzes IL-1β and IL-18 maturation, and simultaneously cleaves GSDMD to trigger pyroptosis (Shi S. et al., 2023; Zhang Q. et al., 2022; Zhang Yun et al., 2021). In DED, tear film instability causes ocular surface hyperosmolarity and oxidative stress, persistently activating the NLRP3 inflammasome and promoting substantial release of IL-1β and IL-18. This triggers inflammatory infiltration of the corneal epithelium, goblet cell apoptosis, and nerve fiber damage. Furthermore, pyroptosis-induced death of lacrimal gland epithelial cells further reduces tear secretion and generates a vicious cycle of “inflammation-tissue damage-tear film deterioration” (Dian et al., 2023).

Oridonin, a bioactive terpenoid sourced from the TCM *Isodon rubescens* (Hemsl.) H. Hara [Lamiaceae; rabdosiae rubescentis herba] (blushred rabdosia leaf), exhibits anti-inflammatory and anti-cancer properties (Li X. et al., 2021). In the *in vitro* experiments, oridonin (2 μM, 4 μM) enhanced migration and recovery of hyperosmotic-stressed (500 mOsm) human corneal epithelial cell-transformed (HCE-T) cells in a dose-dependent manner. In the *in vivo* experiments, topical application of 0.01% oridonin eye drops decreased corneal fluorescein staining scores and increased tear break-up time (BUT), tear volume, and corneal epithelial thickness in the BAC-induced DED mice. Both models confirmed that oridonin reduced NLRP3, Caspase-1, IL-1β, and N-GSDMD expression levels (Li X. et al., 2024). The observed reduction in beneficial effects at higher concentrations (0.1%, 1%) suggests potential cytotoxicity or off-target effects, warranting further investigation.

*Viscum coloratum* (Kom.) Nakai [Santalaceae; visci herba] (coloured mistletoe herb), a member of the mulberry parasitic family, possesses antibacterial and anti-inflammatory properties (Jiang et al., 2017). In the *in vivo* experiments, DED mouse models induced by BAC were treated with 0.005% Huji Sheng eye drops and the NLRP3 pathway inhibitor VX-765, respectively. Both treatments downregulated the expression levels of NLRP3 and Caspase-1 in the conjunctival epithelium and lacrimal gland tissues of mice, reduced serum levels of IL-18 and IL-1β, repaired injured lacrimal gland and conjunctiva, and increased tear secretion. Furthermore, the combined treatment demonstrated greater pharmacological effects (Wu et al., 2024).

#### 5.1.5 Nrf2 signaling pathway

The Nrf2 signaling pathway constitutes a major intracellular anti-oxidant regulatory system and plays a pivotal role in various physiological and pathological processes. Normally, Nrf2 remains at minimal levels in the cytoplasm and is regulated by the negative regulatory factor kelch-like ECH-associated protein 1 (Keap1) which promotes its ubiquitination and degradation through the cullin-3 (Cul3)- RING box protein-1 (Rbx1) complex (Tu et al., 2019). During oxidative stress, critical cysteine sites on Keap1 are oxidized. This results in the destabilization of Keap1-Cul3 ubiquitination machinery and the dose-dependent dissociation of Nrf2 from the Nrf2-Keap1 complex. The released Nrf2 translocates into the nucleus, where it combines with small musculoaponeurotic fibrosarcoma Maf (sMaf) proteins to generate transcriptionally competent Nrf2-sMaf heterocomplexes. These heterodimers induce target gene transcription by recognizing specific antioxidant response elements (AREs) in the target gene promoters (Liu et al., 2022). Target genes include antioxidant enzymes like HO-1, SOD, and GPX, which, when activated, provide cellular antioxidant defense against DED by scavenging free radicals and mitigating ocular surface oxidative damage (Magesh et al., 2012).

Pterostilbene (PS), a single chemical metabolite isolated from the traditional medicinal plant *Pterocarpus santalinus* L. f. [Fabaceae; Pterocarpi Lignum] (red sandalwood), is a resveratrol derivative with significant anti-inflammatory and antioxidant properties (Liu P. et al., 2023). *In vitro* and *in vivo* studies used hyperosmolarity and BAC to generate DED models. Carboxyl-chitosan modified graphene (CG), characterized by hydrophilicity and ocular biocompatibility, was employed as a nanocarrier to improve the hydrophobicity and poor ocular surface retention of PS, forming a novel antioxidant nanocomposite, PS-CG. *In vitro* studies demonstrated that treatment of hyperosmotic-stressed human corneal epithelial cells (HCECs) with PS-CG downregulated reactive oxygen species (ROS) and lactate dehydrogenase (LDH) release, and reduced apoptosis. Mechanistically, PS-CG activated the Keap1-Nrf2-ARE signaling pathway, promoting Nrf2 nuclear translocation and upregulating downstream antioxidant enzymes (e.g., CAT, SOD1). This effect was dependent on Nrf2, as co-treatment with the Nrf2 inhibitor brusatol abolished the protective effect, leading to a resurgence in ROS and decreased antioxidant enzyme expression. Compared to PS alone, PS-CG exhibited superior cellular permeability and a sustained release profile over 30 h. *In vivo* studies using a dry eye mouse model showed that topical application of PS-CG alleviated corneal epithelial damage, increased conjunctival goblet cell density, and promoted tear secretion. These results indicate its pharmacological effects in ameliorating DED symptoms (Lin et al., 2022).

Esculetin (6,7-dihydroxycoumarin), the primary bioactive metabolite found in *F. chinensis subsp. rhynchophylla* (Hance) A.E.Murray [Oleaceae; fraxini cortex] (Chinese ash), shows significant anti-inflammatory and antioxidant properties (Liang et al., 2017). Administration of 20, 40, and 80 μM esculetin promoted Nrf2 nuclear translocation in the H_2_O_2_-mediated DED cellular models in a dose-dependent manner. This treatment upregulated Nrf2, HO-1, NAD(P)H: quinone oxidoreductase 1 (NQO1), SOD1, and SOD2, suppressed the upregulation of BAX and cleaved-Caspase-3, and increased Bcl-2 levels. Moreover, 0.002% esculetin alleviated corneal epithelial damage and restored conjunctival goblet cells in the murine DED model induced by SCOP and desiccating stress (Zhang Yingjun et al., 2021).

Acteoside, the main active metabolite in TCMs such as *Cistanche deserticola* Ma [Orobanchaceae; cistanches herba] (desertliving cistanche) (Shen et al., 2017) and *Rehmannia glutinosa* Libosch. (Di Huang) (Jia et al., 2023), shows antioxidant, anti-inflammatory, neuroprotective, and immunomodulatory properties (Xiao et al., 2022). In the H_2_O_2_-induced DED model HCECs, acteoside pretreatment downregulated Nrf2, HO-1, NQO1, and cyclooxygenase-2 (COX-2) expression levels (Huang and Peng, 2024).

Astaxanthin (AST), a lutein carotenoid primarily derived from *Haematococcus pluvialis* Flot. [Haematococcaceae; Haematococci pulvialis pulvis] (haematococcus pluvialis), possesses antioxidant, anti-inflammatory, anti-apoptotic, and neuroprotective properties (Kim et al., 2022; Si and Zhu, 2022). Network pharmacology was applied to predict the mechanisms of TCM-derived metabolite s including AST in DED. In the study, integrated bioinformatics analyses identified key targets and pathways, such as the Keap1-Nrf2/HO-1 axis, through which AST may alleviate oxidative stress and inflammation. These predictions were experimentally validated in both *in vivo* and *in vitro* models: oral 100 mg/kg AST ameliorated BAC-induced corneal and conjunctival injury in the DED model mice, inhibited MMP9 and IL-1β expression, downregulated Keap1, and upregulated Nrf2 and HO-1 expression levels. Furthermore, 5 μM AST treatment significantly reduced Keap1 expression and ROS levels in the hyperosmotic stress-induced HCECs, elevated HO-1, SOD, and CAT activities, and suppressed the expression levels of IL-6, IL-1β, TNF-α, and MMP-9 (Liu Z. et al., 2025).

#### 5.1.6 SIRT signaling pathway

Sirtuins (SIRT1-SIRT7) are nicotinamide adenine dinucleotide (NAD+)-dependent type III deacetylases, which deacetylate both histone and non-histone targets and regulate energy homeostasis, oxidative stress, inflammation, pyroptosis, and apoptosis (Ning et al., 2024; Singh and Ubaid, 2020). The SIRT pathway plays an important role in cellular antioxidant/redox signaling: SIRT1, SIRT3, and SIRT5 shield cells against ROS; SIRT2, SIRT6, and SIRT7 regulate oxidative stress pathways; and SIRT4 promotes ROS generation and also exert antioxidant effects (Singh et al., 2018).

Puerarin, isolated from *Pueraria montana* var. *thomsonii* (Benth.) M.R.Almeida [Fabaceae; puerariae thomsonii radix] (thomson kudzuvine root), is an isoflavone metabolite with antioxidant, anti-inflammatory, and neuroprotective properties (Zhou et al., 2014). Laboratory studies confirmed that puerarin (10 μM, 30 μM, and 50 μM) inhibited oxidative stress, inflammatory responses, apoptosis, and damage to corneal epithelial barrier in the human carboxylesterase-2 (HCE-2) cells subjected to hyperosmotic conditions in a dose-dependent manner. This protective mechanism was regulated by the SIRT1/NLRP3 pathway—specifically by enhancing SIRT1 activity while suppressing NLRP3 expression. Furthermore, puerarin treatment resulted in reduced BAX/Bcl-2 ratio and cleaved-Caspase-3 levels, diminished secretion of pro-inflammatory cytokines (TNF-α, IL-6, IL-1β) and reactive oxygen species, elevated activity of antioxidant enzymes (SOD, CAT), increased tight junction protein expression (zonula occludens-1 (ZO-1), occludin), and improved GSH/GSSG ratio. Furthermore, SIRT1-targeted siRNA attenuated the protective effects of puerarin (Dong et al., 2024).

Oroxylin A (OA) is derived from *Scutellaria baicalensis* Georgi [Lamiaceae; scutellariae radix] (baical skullcap root) and exhibits antioxidant, anti-inflammatory, cytoprotective, and neuroprotective effects (Sajeev et al., 2022; Wang P.-X. et al., 2024). *In vitro* experiments with hyperosmotic-induced DED cell model showed that 10 μg/mL OA enhanced the activity of SIRT3 and SOD2, while decreasing the synthesis of hypoxia-inducible factor-1α (HIF-1α), ROS, and NO. It also lowered the concentrations of NLRP3, Caspase-1, cleaved-Caspase-1, N-GSDMD, Caspase-3, and BAX/Bcl-2 ratio. These protective effects were reversed by the SIRT3 inhibitor, thereby suggesting that OA attenuates pyroptosis and apoptosis by activating SIRT3-SOD2/HIF-1α pathway (Liu X. et al., 2025).

#### 5.1.7 AMPK signaling pathway

Adenosine monophosphate-activated protein kinase (AMPK) serves as a key regulator of cellular energy balance and metabolic homeostasis, and responding to energy stress (e.g., hypoglycemia, hypoxia, oxidative stress) by sensing fluctuations in the AMP(ADP)/ATP ratio. Liver kinase B1 (LKB1) activates AMPK by phosphorylating the α-subunit of AMPK specifically at the threonine 172 (Thr172) residue (Elijah and Reuben, 2021). AMPK signaling pathway regulates metabolism, autophagy, antioxidant pathway, and anti-inflammatory effects, and is closely related with the pathological response to DED (Cantó et al., 2009; Herzig and Shaw, 2018).

Chlorogenic acid (CGA), a polyphenolic active metabolite in *Xanthium strumarium* L. [Asteraceae; xanthii fructus] (siberian cocklebur fruit), exhibits potent antioxidant and anti-inflammatory properties (Vyacheslav et al., 2021). During autophagy, LC3-I converts to LC3-II; and LC3-II/LC3-I ratio is positively correlated with autophagic activity (Lr et al., 2020). Conversely, autophagic substrate p62 is degraded in the lysosomes, its expression shows negative correlation with autophagy (Trond et al., 2017). *In vivo* experiments using the SOD1^−/−^ mice as the oxidative stress-induced DED mice demonstrated that intramuscular administration of 50 mg/kg CGA solution inhibited AMPK and UNC-51-like kinases 1 (ULK1) phosphorylation, downregulated autophagy core protein Beclin1 and LC3-II/LC3-I ratio, upregulated p62, lowered MDA and ROS levels, decreased Bax and Caspase-3 levels, elevated Bcl-2 levels, increased tear secretion, and reduced corneal epithelial damage. These results indicate that CGA alleviates oxidative damage, apoptosis, and autophagy by inhibiting AMPK/ULK1 (Chen H. et al., 2025). The correlation between the use of intramuscular injection as a administration route for DED and clinical practice should be further considered.

Salidroside, a key bioactive metabolite in *Rhodiola rosea* L. [Crassulaceae; rhodiolae crenulatae radix et rhizoma] (arctic root), exhibits anti-inflammatory, antioxidant, and immune-regulating properties (Magani et al., 2020; Tao et al., 2019). SIRT1 attenuates inflammation by suppressing the NF-κB and AP-1 pathways and decreasing ROS by modulating mitochondrial function. It shows positive feedback regulation with AMPK, wherein AMPK upregulates NAD^+^ levels to activate SIRT1, whereas SIRT1 enhances AMPK activity by deacetylating LKB1, an upstream kinase of AMPK (Wang L. et al., 2018). *In vitro* and *in vivo* experiments using 100 μM salidroside and 0.5 mM and 2 mM salidroside eye drops in the DED cellular and mouse models, respectively, showed that salidroside activated AMPK/SIRT1 pathway, upregulated LC3-II levels, induced Nrf2 translocation into the nucleus, and enhanced the synthesis of its downstream proteins, HO-1 and NQO1. The treatment also significantly decreased MDA levels, increased the activities of SOD and CAT enzymes, and reduced the levels of TNF-α, IL-1β, and IL-6. Furthermore, autophagy inhibitor chloroquine (CQ) and AMPK inhibitor compound C (dorsomorphin) reversed these effects, thereby confirming the mechanism of action (Liang et al., 2023).

#### 5.1.8 VEGF signaling pathway

The vascular endothelial growth factor (VEGF) family comprises VEGF-A, VEGF-C, VEGF-D, and other members, which regulate angiogenesis, lymphangiogenesis, and inflammation by binding to the vascular endothelial growth factor receptor (VEGFR). Binding of VEGF-A to VEGFR-2 activates the PI3K-AKT, ERK, and endothelial nitric oxide synthase (eNOS) pathways, thereby promoting endothelial cell proliferation, migration, and vascular permeability; VEGF-A acts as a core regulator of physiological angiogenesis (e.g., embryonic development) and pathological neovascularization (Apte et al., 2019; Feliers et al., 2005). VEGF-C/D promotes differentiation, proliferation, and lymphangiogenesis of lymphatic endothelial cells and participates in tissue fluid drainage and immunomodulation by binding to VEGFR-3 and activating the ERK and PI3K/AKT pathways (Melincovici et al., 2018). VEGF can engage in positive feedback with inflammatory factors (e.g., IL-6, IL-1β, TNF), and enhances vascular leakage and immune cell infiltration in the inflammatory microenvironment (Tai et al., 2021). In DED, desiccation stress induces corneal lymphangiogenesis in the limbal arch of the cornea through aberrant activation of the VEGF pathway, thereby inducing immune cells to migrate to the ocular surface through the nascent lymphatic vessels, disrupting ocular surface homeostasis, and releasing pro-inflammatory cytokine IL-17. This further promotes VEGF/VEGFR interaction and facilitates corneal lymphangiogenesis (Patnam et al., 2023).

Epigallocatechin gallate (EGCG), the major polyphenol in *Camellia sinensis* (L.) Kuntze [Theaceae; Theae Folium] (green tea), exhibits antioxidative, anti-angiogenesis, and anti-inflammatory properties (Luo et al., 2019). An animal experiment used 0.1% and 0.01% EGCG solutions for topical treatment of a DED mouse model induced by subcutaneous injection of SCOP combined with controlled environmental conditions. The results showed that 0.1% EGCG significantly reduced corneal fluorescence staining and apoptotic epithelial cells in the DED model mice. The expression levels of IL-1β, C-C motif ligand 2 (CCL2), and inflammatory cell marker CD11b^+^ in the cornea were significantly downregulated, while the levels of VEGF-A and VEGF-D were significantly reduced. These findings demonstrate that EGCG alleviates corneal epithelial injury, inflammatory response, and apoptosis in DED mice by inhibiting the VEGF signaling pathway (Lee et al., 2011).

*Fraxinus chinensis subsp. rhynchophylla* (Hance) A.E.Murray [Oleaceae; fraxini cortex] (Chinese ash) contains various active metabolites, such as coumarins, which exhibit anti-inflammatory and antioxidant properties (Zheng Z. et al., 2024). Treatment of DED mice induced by environmental factors with Qin Pi eye drops significantly downregulated VEGF-C/VEGFR-3 expression, reduced corneal neoplastic lymphatic vessels and CD4^+^ T cell infiltration, suppressed IL-1β/IL-6 and enhanced TGF-β1/IL-10 expression levels, and restored ocular surface homeostasis (Hu, 2017).

#### 5.1.9 Other signaling pathways

The signal transducer and activator of transcription (STAT) 1 signaling cascade represents a critical arm of the Janus kinase (JAK)-STAT family. The core components of this cascade include cell surface receptors (e.g., IFN receptors IFNAR/IFNGR), protein tyrosine kinases (JAK1/JAK2), and the transcription factor STAT1 (Stark and Darnell, 2012). JAK is activated when IFNs (e.g., IFN-α/IFN-γ) or certain interleukins (e.g., IL-6) bind to their receptors. JAK phosphorylates the tyrosine residues in the intracellular segments of the receptor and provides SH2 structural domain binding sites for STAT1. Subsequently, STAT1 is phosphorylated by JAK at the Tyr701 and Ser727 sites, and forms either a homodimer or a heterotrimeric complex (ISGF3) with STAT2/IRF9. This complex translocates into the nucleus and binds to γ-activating sequences (GAS) or interferon-stimulated response elements (ISRE) to initiate transcription of interferon-stimulated genes (ISGs) and interferon regulatory factors (IRFs) (Au-Yeung et al., 2013; Barrat et al., 2019; Peng et al., 2023). STAT1 pathway regulates antigen presentation, autoimmunity, and inflammation (Barrat et al., 2019)). In the pathogenesis of DED, hyperosmolar environments or oxidative stress stimulates increased expression of IFN-γ in the corneal epithelial cells and activation of the STAT1 pathway. This promotes pyroptosis of corneal epithelial cells, release of inflammatory mediators (e.g., IL-1β, IL-18) and chemotactic signals (e.g., C-X-C motif ligand 10 (CXCL10)), and disruption of ocular surface microenvironment homeostasis (Yang X. et al., 2023). Treatment of H_2_O_2_-induced lacrimal gland epithelial cells with 8.95 × 10^−2^ mol/L Buddleia flavonoid-containing plasma (Buddleia flavonoid extract from *B. officinalis* Maxim. [Scrophulariaceae; buddlejae flos] (pale butterflybush flower) upregulated the phosphorylation of STAT1, activated the STAT1 signaling pathway, simulated the androgen effect by binding to androgen receptors, and inhibited oxidative stress-induced apoptosis (Wang et al., 2010).

Notch signaling pathway regulates cellular fate, differentiation, proliferation, and apoptosis. Binding of Delta-like ligands ((DLL1/3/4) and jagged ligands (Jagged1/2) on neighboring cells to Notch receptors (Notch1-Notch4) activates Notch target genes transcription (Hairy/Enhancer of Split (HES), Hairy/Enhancer of Split related to YRPW motif (HEY)) via Notch intracellular domain (NICD)/CBF-1/suppressor of hairless/Lag1 (CSL)-dependent transcription. Furthermore, it regulates downstream factors via CSL-independent cellular responses, including Notch transmembrane domain (NTMD)-dependent activation of Ras-associated protein Rac1, NICD-dependent stimulation of NF-κB, and NICD-dependent suppression of serine protein kinase ataxia-telangiectasia mutated (ATM) (Katoh and Katoh, 2020; Takebe et al., 2014). Resveratrol (RES), derived from *R. japonica* Houtt. [Polygonaceae; polygoni cuspidati rhizoma et radix] (giant knotweed rhizome), exhibits anti-inflammatory and antioxidant properties (Fan et al., 2021). In the *in vitro* experiments, 25 mM RES downregulates the Bax/Bcl-2 ratio and ROS levels while upregulating the levels of SOD2, HO-1, vitamin D receptor (VDR), 25-hydroxyvitamin D, Notch2, Notch4, Dll3, Jagged1, and Hes1 in HCECs under hypertonic conditions. These findings confirm that RES alleviates apoptosis and oxidative stress by activating the Notch pathway (Shetty et al., 2020).

Activation of the protein kinase A (PKA)/cyclic adenosine monophosphate (cAMP)-response element binding protein (CREB) pathway depends on cAMP-PKA-mediated phosphorylation of CREB at the Ser-133 site (Li H. et al., 2022; Liu et al., 2017; Rashid and Kim, 2016). PKA/CREB signaling pathway regulates tear production and ocular surface inflammation (Cammalleri et al., 2021; Ru et al., 2015). Polydatin (PD), a bioactive metabolite derived from *R. japonica* Houtt. [Polygonaceae; polygoni cuspidati rhizoma et radix](giant knotweed rhizome), exhibits anti-inflammatory and antioxidant properties (Karami et al., 2022). Topical eye drops containing 0.05% PD and 0.5% PD significantly elevated corneal p-PKA and p-CREB levels, decreased IL-1β, TNF-α, and IL-6 expression levels, increased tear secretion and goblet cell density, and alleviated corneal damage in the DED model rats. These findings suggest that PD improves tear secretion and reduces inflammation by activating the PKA/CREB pathway (Zhao and Wei, 2024).

Both p53 and mTOR belong to the autophagy-related gene (ATG) protein family and regulate autophagy through distinct mechanisms (Chen J. et al., 2025; Khalil et al., 2023). P53 promotes autophagy either by directly triggering autophagy regulators or through interactions with them (Klionsky et al., 2021; Levine and Kroemer, 2019), whereas mTOR is a critical player in autophagy initiation and development (Chen J. et al., 2025). P53 enhances autophagy through mTOR regulation and activation of autophagy-related genes such as sestrin-2 and damage-regulated autophagy modulator (DRAM-1) (Feng et al., 2005). In DED, oxidative stress causes ocular surface cell damage, but this is mitigated by autophagy through elimination of damaged organelles and aggregated proteins (Zhang et al., 2025). Evodiamine (EVO), the main active metabolite of *Tetradium ruticarpum* (A.Juss.) T.G.Hartley [Rutaceae; euodiae fructus] (medicinal evodia fruit), shows anti-inflammatory, pro-apoptotic, and autophagy-promoting bioactivities (Chen S. et al., 2025; Lin et al., 2024). In the *in vivo* and *in vitro* studies, treatment of DED mice models with 500 μM and 1 mM EVO and HCEC DED model cells with 0.1 μM EVO demonstrated that EVO increased nuclear p-p53 and LC3-II levels, reduced cytoplasmic p-p53, p-mTOR, and p62 levels, downregulated the levels of inflammatory cytokines TNF-α, IL-6, and IL-1β, decreased the levels of ROS and MDA, and enhanced the activity of antioxidant enzymes SOD and CAT. These results indicate that EVO enhances autophagy and alleviates inflammatory responses and oxidative stress via the p53/mTOR pathway (Li et al., 2025).

MUC1, a type I transmembrane protein, regulates cell growth, proliferation, and apoptosis, while epidermal growth factor receptor 1 (EGFR/ErbB1/HER1) regulates cell proliferation, survival, and differentiation (Cui H. et al., 2024; Zhang et al., 2023). The cytoplasmic tail (CT) of MUC1 interacts with EGFR through mediation by the sarcoma kinase (Src kinase) and forms a positive feedback loop. MUC1-CT binds to the EGFR kinase domain via a tyrosine residue (Y46). After EGFR activation, it phosphorylates the Y60 site of MUC1-CT, which inhibits ubiquitinated degradation of EGFR and enhances its autophosphorylation at Y1068 and Y1173. This synergistically activates downstream pathways including NF-κB, MAPK, and PI3K/AKT (Xu et al., 2024; Zhang et al., 2023). MUC1 serves as a major mucin component in the tear film, and its deficiency may lead to ocular surface barrier breakdown and DED development (Ablamowicz and Nichols, 2016). Treatment of DED rabbit models induced by BAC with 5 μM and 10 μM Astragalus IV, extracted from *Astragalus mongholicus* Bunge [Fabaceae; Astragali radix] (milkvetch root), significantly increased tear production and conjunctival goblet cell count, and upregulated MUC1 and EGFR expression levels. These findings suggest that Astragalus IV ameliorates ocular surface damage through the MUC1/EGFR pathway (Chu et al., 2021).

Transforming Growth Factor-beta (TGF-β)/Smad signaling pathway is a central pathway mediating fibrosis, tissue repair, and immune regulation across various organs (Verrecchia and Mauviel, 2002). In the context of DED, chronic inflammation is associated with dysregulation of this pathway at the ocular surface. Specifically, conjunctival epithelium from DED patients exhibits upregulation of TGF-β2 and TGF-β3 mRNA (Benito et al., 2013), indicating its involvement in disease pathology. While it is essential for normal wound healing and maintaining immune tolerance, its persistent activation is implicated in pathological processes relevant to severe DED, such as corneal fibrosis (scarring) and conjunctival squamous metaplasia (Guo et al., 2018). Although none of the TCM studies reviewed herein explicitly identified the TGF-β/Smad pathway as a primary mechanistic target in DED, several formulas and metabolites, such as ganoderma lucidum polysaccharide (Chen C. et al., 2023) and ferulic acid (Mu et al., 2018), have been reported to modulate this pathway in other disease models related to fibrosis and immunoregulation. Its role in DED and potential modulation by TCM merits exploratory study.

### 5.2 Multiple signaling pathways

The pathogenesis of DED involves a network of interconnected signaling pathways rather than isolated cascades. As illustrated throughout Section 4.1, these pathways do not function in isolation but engage in extensive crosstalk, forming a complex regulatory network that perpetuates the vicious cycle of DED. For instance, the PI3K/AKT pathway intersects with NF-κB and mTOR signaling to modulate both survival and inflammatory responses. The MAPK and NF-κB pathways often act synergistically to amplify pro-inflammatory gene expression. Meanwhile, the Nrf2 antioxidant pathway antagonizes NF-κB-driven inflammation, and SIRT1 can suppress NLRP3 inflammasome activation via deacetylation. This intricate interplay underscores the necessity of multi-target interventions. The following subsections highlight prominent examples of such pathway crosstalk, particularly focusing on NF-κB-related networks, to exemplify how TCM achieves holistic regulation by simultaneously modulating multiple nodes within this pathological web.

#### 5.2.1 NF-κB-related signaling pathways

The NF-κB pathway is a major contributor to the pathological mechanism of DED due to its pro-inflammatory properties and bidirectional regulation of apoptosis. Its activation is driven by TLR4 and PI3K/AKT signaling, and the inflammatory cascade is further amplified synergistically with MAPK signaling. Meanwhile, Nrf2 antagonizes this process via the antioxidant pathway. Hyperosmotic stress and ROS accumulation in DED disrupt the homeostasis of these pathways and generate a vicious cycle of “inflammation-oxidative stress-apoptosis”. Therefore, combining anti-inflammatory, antioxidant, and anti-apoptotic strategies targeting the intersection of multiple pathways (e.g., NF-κB and ROS) may enable precise intervention in DED.

##### 5.2.1.1 NF-κB and MAPK signaling pathways

Hypertonic environment and ROS concurrently activate NF-κB and MAPK pathways. These pathways exhibit significant crosstalk: NF-κB and AP-1 (a downstream effector of MAPK) can act synergistically to enhance the transcription of common target genes, including MMP-9, inducible nitric oxide synthase (iNOS), TNF-α, monocyte chemoattractant protein-1 (MCP-1), and IL-6 (Atsaves et al., 2019; Wang T. et al., 2018). ERK1/2 and p38 MAPK enhance p65 transcriptional activity via mitogen- and stress-activated protein kinase 1 (MSK1) phosphorylation (Galán-Ganga et al., 2019). MAPK-activated ROS mediates IκB degradation and amplifies NF-κB activity, whereas inflammatory factors induced by NF-κB (e.g., tumor necrosis factor-α (TNF-α)) feedback into the MAPK pathway and form a continuous inflammatory positive feedback loop. These synergistic effects lead to goblet cell apoptosis, abnormal tear film lipids, and neurogenic inflammation. This exacerbates ocular surface damage and reduces tear secretion. Therefore, targeting both pathways simultaneously may be an effective DED treatment strategy.

*In vitro* and *in vivo* models confirmed that 250 μg/mL *Dendrobium officinale* Kimura and Migo [Orchidaceae; dendrobii officinalis caulis] (noble dendrobium) water extract (DOW) and 500 μg/mL *Dendrobium loddigesii* Rolfe [Orchidaceae; dendrobii caulis] (loddiges’ dendrobium) water extract (DLW) enhanced tear production, protected conjunctival goblet cells, minimized ocular surface damage, reduced the release of MMPs, upregulated lacrimal gland AQP5 and MUC5AC levels, decreased p-ERK, p-p38, and p-NF-κB expression levels, and inhibited the activation of the corresponding NF-κB and MAPK signaling pathways (Ling et al., 2022).

Linarine, a naturally occurring bioactive metabolite, is prevalent in several TCMs such as *B. officinalis* Maxim. [Scrophulariaceae; buddlejae flos] (pale butterflybush flower), *Chrysanthemum × morifolium* (Ramat.) Hemsl. [Asteraceae; Chrysanthemi flos] (chrysanthemum), *Mentha canadensis* L. [Lamiaceae; menthae haplocalycis herba] (peppermint), and exhibits anti-inflammatory, antioxidant, and anti-apoptotic properties (Mottaghipisheh et al., 2021). In HCECs under hyperosmolar stress, 15 µM linarine effectively decreased TNF-α and IL-1β levels. In a mouse model of DED, oral linarine (12.5, 25, 50 mg/kg/day) enhanced tear production and stabilized the tear film while promoting healing in damaged corneal and lacrimal gland tissues. Mechanistically, it suppressed corneal expression of p38, JNK, p65, IL-1β, and IL-18, indicating significant anti-inflammatory effects (Liu et al., 2024a).

Paeoniflorin is the primary bioactive metabolite extracted from *Paeonia lactiflora* Pall. [Paeoniaceae; Paeoniae radix alba] (white peony root) and possesses anti-inflammatory and immunomodulatory properties (Zhang and Wei, 2020). A study using 0.01%, 0.1%, and 1.0% paeoniflorin in hyperosmolarity (450 mOsM)-induced HCECs and experimental dry eye (EDE) mouse models demonstrated that paeoniflorin significantly ameliorated tear secretion, corneal epithelial detachment, and ocular surface inflammation. Furthermore, paeoniflorin downregulated IL-1, IL-6, TNF-α, NF-κB, p-p38 MAPK, and p-JNK levels in the HCECs in a dose-dependent manner (Zhao et al., 2019).

Lutein, a natural active substance in dark green leafy vegetables, can be extracted from the TCM *Calendula officinalis* L. [Asteraceae; calendulae flos] (calendula) (Kim et al., 2024). It exhibits antioxidant and free radical-scavenging properties, and is highly expressed in the retina macula absorbing high-energy blue light (Li et al., 2020). It also has anti-inflammatory properties and inhibits the synthesis of COX-2, iNOS, and NF-κB (Kijlstra et al., 2012). In the *in vitro* experiments, treatment of hyperosmolarity-induced HCECs with 1, 3, and 10 μM lutein inhibited IL-6 secretion in a dose-dependent manner. Among these, 10 μM lutein significantly downregulated NF-κB, p-p38 MAPK, and p-JNK1/2 levels. Furthermore, cells pretreated with p38 inhibitor (SP 203580) or JNK inhibitor (SP 600125) before exposure to the hypertonic medium significantly decreased IL-6. This demonstrated that lutein inhibits hyperosmolarity-induced IL-6 secretion in the HCECs by inhibiting the p38, JNK, and NF-κB pathways (Chao et al., 2016).

KIOM-2015EW is a hot-water extract derived from *A. palmatum* Thunb. [Sapindaceae; Not recorded in pharmacopeia] (Japanese maple). In the *in vitro* experiments, KIOM-2015EW (0.05, 0.1, and 0.2 mg/mL) downregulated TNF-α, IL-1β, and IL-6 levels in the hyperosmotic HCECs in a dose-dependent manner. It also inhibited Caspase-3 and PARP cleavage, increased Bcl-2 levels, decreased p-p38, p-ERK, p-JNK, p-IκB, levels, and suppressed nuclear translocation of NF-κB. Combined treatment with p38 inhibitor (SB203580), ERK inhibitor (PD98059), and JNK inhibitor (SP600125) effectively blocked HOS-induced cytotoxicity and apoptosis (Kim et al., 2018). Therefore, KIOM-2015EW significantly attenuates HOS-induced inflammatory responses and apoptosis by suppressing MAPK and NF-κB signaling pathway.

##### 5.2.1.2 NF-κB and NLRP3 signaling pathways

NF-κB and NLRP3 pathways are functionally complementary. NF-κB is activated in response to primary signals (LPS and hyperosmotic stress) and upregulates pro-inflammatory genes, including NLRP3, pro-IL-1β, and IL-18, thereby providing a molecular basis for subsequent assembly of the inflammasome, which executes the inflammatory effect; the second signal (e.g., ROS, ATP, or hyperosmotic environment) triggers NLRP3 oligomerization through assembly of ASC and Caspase-1 into the inflammasome, which then catalyzes IL-1β/IL-18 maturation and triggers pyroptosis, thereby amplifying the NF-κB-mediated inflammatory responses (Bauernfeind et al., 2009; Kelley et al., 2019). In DED, both these pathways coordinately drive ocular surface inflammation and tear film destruction.

Aurantio-obtusin (AO), an active anthraquinone from *Senna tora* (L.) Roxb. [Fabaceae; cassiae semen] (cassia seed), exhibits anti-inflammatory properties (Kim et al., 2015; Kwon et al., 2018). In BAC-induced DED rats, topical application of a 0.5% AO solution enhanced tear secretion, attenuated ocular surface destruction, preserved goblet cell numbers, and significantly decreased TNF-α, IL-6, and MCP-1 levels in both conjunctival and corneal tissues. Furthermore, AO treatment increased the levels of anti-inflammatory cytokine IL-10 levels, downregulated the expression levels of key components of the NLRP3 inflammasome pathway, including NLRP3, ASC, cleaved-Caspase-1, and IL-1β, and significantly inhibited the phosphorylation of p65, IKKβ, and IκBα (Zhu et al., 2024).

Polydatin, the bioactive metabolite found in *R. japonica* Houtt. [Polygonaceae; polygoni cuspidati rhizoma et radix] (giant knotweed rhizome), exerts therapeutic effects in DED. In lacrimal gland-resected DED rats, 0.5% polydatin significantly ameliorated pathological changes, including tear reduction, corneal damage, and tear film rupture, and downregulated IL-1β, IFN-γ, TNF-α, and IL-6 by inhibiting the NLRP3 inflammasome. In the *in vitro* experiments with hyperosmosis-induced cells, treatment with 10 μM polydatin reduced oxidative stress and inflammation by blocking the NLRP3 inflammasome and NF-κB signaling pathways, thereby lowering the levels of ROS, TNF-α, IL-6, IL-1β, MMP9, and COX-2 (Park et al., 2019).

β-Aminoarteether maleate (SM934), a water-soluble artemisinin derivative from *Artemisia annua L.* [Asteraceae; artemisiae annuae herba] (sweet wormwood herb), shows anti-inflammatory, immunomodulatory, and cytoprotective properties (Li T.et al., 2015; Yan et al., 2018). In the SCOP-induced rodent models and BAC-induced rat models of DED, topical application of 0.1% and 0.5% SM934 effectively improved tear production, tear film stability, and corneal damage by decreasing MMP-9, TNF-α, IL-6, IL-1β, and MCP-1 levels, suppressing the accumulation of TLR4 macrophages in the conjunctiva, and inhibiting the expression levels of myeloid differentiation primary response protein 88 (MyD88), NLRP3, ASC, and cleaved-Caspase-1. Furthermore, pretreatment of LPS-treated RAW 264.7 cells with 10 μM SM934 significantly reduced the expression levels of TLR4, p-NFκB, NLRP3, ASC, and cleaved-Caspase-1 (Yang et al., 2021).

##### 5.2.1.3 Other NF-κB-related multiple signaling pathways

Pterostilbene is a natural metabolite with antioxidant and anti-inflammatory properties, but its low bioavailability has limited its therapeutic applications. To overcome these limitations, a nanomodicine approach was used by dissolving the Pterostilbene-glutaricanhydride-arginine-glycine-aspartic acid (PS-GA-RGD) monomer in phosphate-buffered saline (PBS). *In vitro*, 10 μM PS-GA-RGD suppressed inflammation and oxidative stress in macrophages by inhibiting NF-κB pathway and activating Nrf2 pathway. *In vivo*, topical application of PS-GA-RGD (5 mg/mL) in a murine DED model significantly reduced corneal epithelial apoptosis and downregulated IL-6, IL-1β in both corneal and conjunctival tissues, consistent with its anti-inflammatory mechanism identified *in vitro* (Li, 2023).

Gallic acid (GA) (3, 4, 5-trihydroxybenzoic acid) is abundant in *Phyllanthus emblica* L. [Phyllanthaceae; phyllanthi fructus] (emblic leafflower fruit) and exhibits significant anti-inflammatory and antioxidant properties (Bai et al., 2021). Treatment of hyperosmotic stress-induced HCECs and LPS-induced RAW264.7 cells with 100 μM GA significantly reduced ROS, IL-6, TNF-α, NO, p-IκB and p-p65 levels, and upregulated p-Nrf2, HO-1, and NQO1 expression levels. In the *in vivo* experiments with DED model mice, administration of 5 mg/mL GA eye drops significantly alleviated corneal injury and apoptosis, maintained the number of conjunctival goblet cells, and downregulated IL-6 and IL-1β levels (Li et al., 2023). This suggested that GA exerts potent anti-inflammatory and antioxidant properties by inhibiting the NF-κB signaling pathway and activating the Nrf2 pathway.

In the *in vivo* DED model mice, topical administration of 0.5 or 2 mg/mL berberine (BBR) from *C. chinensis* Franch. [Ranunculaceae; coptidis rhizoma](Chinese goldthread) promoted tear secretion, restored the numbers of conjunctival goblet cells, and inhibited the activation of PI3K/AKT/NFκB and MAPK signaling pathways. Furthermore, BBR suppressed the expression levels of IL-1α, IL-1β, IL-6, IL-17, TNF-α, and IFN-γ, downregulated the levels of MMP-3 and MMP-9 on the ocular surface, and decreased the levels of CD4^+^ T cells, cleaved Caspase-3, and cleaved Caspase-8. Complementary *in vitro* studies demonstrated that BBR (2.5, 5 µM) directly protects human corneal epithelial cells from hyperosmotic stress-induced damage by enhancing cell viability and, mechanistically, by inhibiting the nuclear translocation of NF-κB p65 (Han Yi et al., 2023).

#### 5.2.2 Other multiple signaling pathways

Hyperosmotic stress-induced activation of the ROS-NLRP3-IL-1β signaling axis is an important initiator of epithelial inflammation in DED (Dai et al., 2019). Nrf2 is a critical endogenous antioxidant defense regulator. During oxidative stress, Nrf2 undergoes nuclear translocation, binds to AREs, and initiates transcription of antioxidant enzyme genes, thereby exerting protective effects against oxidative damage (Li J. et al., 2021). Laboratory experiments with immortalized human corneal epithelial cells (iHCECs) subjected to hyperosmotic stress showed that treatment with 50 μM genistein, isolated from *Spatholobus suberectus* Dunn [Fabaceae; spatholobi caulis] (suberect spatholobus stem), significantly inhibited NLRP3 and Caspase-1 activation, and significantly reduced the secretion of IL-1β and IL-18, as well as ROS levels. This reduced the number of 8-hydroxy-2′-deoxyguanosine (8-OHdG)-positive cells. Genistein suppressed ROS-NLRP3-IL-1β pathway and alleviated inflammation-associated oxidative damage by activating the Nrf2 pathway and enhancing SOD, CAT, and glutathione reductase (GR) activity (Zhou et al., 2020).

## 6 TCM formulas and signaling pathways

Beyond the specific active metabolites found in TCM, TCM formulations offer advantages in clinical disease management through multi-component synergy, multi-target engagement, and multi-pathway regulation. The signaling pathways engaged by Multi-herb TCM formulas are depicted in Figure 2B, illustrating the multi target regulation of interconnected pathways based on the evidence summarized in the tables. Data for TCM formulas are integrated into Table 2 (*in vivo*) and Table 3 (*in vitro*). Their specific multi-component compositions and standardization status are detailed separately in Supplementary Table S3. Corresponding experimental parameters are included in Supplementary Table S4.

### 6.1 Yangyin Runmu Pills

Yangyin Runmu Pills are an in-hospital preparation developed by the First Affiliated Hospital of Hunan University of Chinese Medicine with primary functions to nourish the liver and kidneys according to TCM theory. In addition to the three main studies detailed below, extensive research on this formula has validated its multi-faceted mechanisms, including the suppression of key inflammatory mediators (e.g., TNF-α and NF-κB) (Li et al., 2016), inhibition of apoptosis (e.g., via Bax/Bcl-2) (Li Y., 2021), and attenuation of pyroptosis (e.g., via NLRP3/Caspase-1) (Li, 2022).

Surgical castration was used to develop a DED rat model. Subsequently, after a week of model establishment, a group of rats were administered Yangyin Runmu Pills mixed with normal saline at a concentration of 9 g/100 mL via oral gavage for 3 months. Compared with the DED model group, the Yangyin Runmu Pills group rats showed significantly higher Schirmer’s test (SIT) values and tear film stability. Moreover, the experimental group showed significant downregulation of intercellular adhesion molecule-1 (ICAM-1), p38MAPK, and p-p38MAPK in the rat conjunctival epithelial cells. Therefore, Yangyin Runmu Pills mitigates DED by blocking inflammatory responses through the p38MAPK signaling pathway (Li D. et al., 2015).

Another study investigated the effects of Yangyin Runmu Pills (0.782, 0.869, and 1.738 g/kg/d) on the SCOP-induced DED rabbit models induced by SCOP. The results demonstrated that Yangyin Runmu Pills increased p38MAPK levels in the lacrimal gland tissue of DED model rabbits in a dose-dependent manner and restored the Bax/Bcl-2 ratio. This reduced lacrimal gland cell apoptosis, restored lacrimal gland cell metabolic function, and increased tear secretion (Li et al., 2019).

Another *in vitro* study established a DED cell model by inducing injury in lacrimal gland cells with TNF-α. To prepare drug-containing serum, rabbits were orally administered Yangyin Runmu Pills at 6.952 g/kg/d. The TNF-α-induced cells were then treated with this serum for 24 h. Compared with the model group, the treatment group showed a significantly higher cell survival rate and significantly lower IL-1β and IL-6 levels in the cell supernatant, and reduced expression levels of TNF-α, p38MAPK, and p53. These findings suggest that Yangyin Runmu Pills reduce the secretion of inflammatory factors and enhance cell survival by inhibiting TNF-α/p38MAPK/p53 signaling pathway (Li D. et al., 2022).

In conclusion, effective treatment of DED by Yangyin Runmu Pills involves alleviation of ocular surface dryness by attenuating inflammatory responses and reducing lacrimal gland cell apoptosis via TNF-α/p38MAPK/p53 signaling pathways.

### 6.2 Qingxuan Runmu Decoction

Qingxuan Runmu Decoction (QXRMY) is derived from Humor-Increasing Decoction, as described in the “Systematic Differentiation of Warm Diseases” (Zhao et al., 2024a). Administration of 0.6 g/mL QXRMY to BAC-induced DED rats for 2 weeks significantly improved SIT scores, reduced fluorescein staining (FL) scores, ameliorated corneal morphology, and significantly decreased TNF receptor associated factor 6 (TRAF6), IL-1β, TNF-α, and MMP9 levels and the phospho-Transforming growth factor-β-activated kinase 1 (p-TAK1)/TAK1 ratio compared to the model group. This indicates that QXRMY alleviates corneal damage, inhibits inflammation, and mitigates DED symptoms by inhibiting the TRAF6/TAK1 pathway (Zhao et al., 2024a).

In another *in vivo* study, treatment of BAC-induced DED rats with 9 g/kg/d QXRMY via gavage for 14 days significantly increased SIT scores, decreased FL scores, improved corneal and conjunctival structure, restored conjunctival epithelial morphology, increased the number of conjunctival goblet cells, and decreased TLR4, MyD88, and p-p65 expression levels in the cornea, conjunctiva, and lacrimal glands compared to the model group. This suggests that QXRMY mitigates ocular surface injury and alleviates DED symptoms by inhibiting the TLR4/MyD88/NF-κB pathway (Zhao et al., 2024b).

Another study integrated network pharmacology with experimental validation to predict and verify the mechanism of QXRMY. Through this approach, heme oxygenase-1 (HMOX1) was identified as a key target, leading to the hypothesis that QXRMY alleviates DED via the HIF-1α/HMOX1 pathway. This hypothesis was subsequently validated using a hyperosmotic DED model in HCE-2 cells and lacrimalectomy-induced DED rats. The experimental results demonstrated that QXRMY enhances tear secretion, alleviates corneal injury, and stabilizes the tear film by suppressing ferroptosis through inhibiting the HIF-1α/HMOX1 pathway. This was evidenced by a significant reduction in oxidative stress and levels of ferroptosis promoters (TFRC and ACSL4), alongside an upregulation of the ferroptosis inhibitor GPX4 (Wang J. et al., 2024). The findings from the experimental validation were consistent with the initial network pharmacology predictions.

In summary, QXRMY suppresses inflammation by inhibiting the TRAF6/TAK1 signaling pathway, prevents ferroptosis by suppressing the HMOX1/HIF-1α pathway, and alleviates DED symptoms and repairs ocular surface damage by inhibiting the TLR4/MyD88/NF-κB signaling pathway.

### 6.3 Qishen prescription

Qishen prescription consists of two classical TCM formulas, Qiju Dihuang Pills and Shengmai Decoction, supplemented with *Tetrapanax papyrifer* (Hook.) K. Koch [Araliaceae; tetrapanacis medulla] (ricepaperplant pith), *Polygonatum sibiricum* Redouté [Asparagaceae; Polygonati rhizome] (solomonseal rhizome), and *P. montana* var. *thomsonii* (Benth.) M.R.Almeida [Fabaceae; puerariae thomsonii radix] (thomson kudzuvine root). An *in vitro* experiment showed that the Qishen prescription significantly downregulated IL-1β and IL-8 by inhibiting HS-induced MAPK pathway phosphorylation (specifically p38MAPK, and ERK1), and reducing the expression levels of Caspase-3 protein (Zhao et al., 2022b).

Another *in vitro* study confirmed that treatment of hyperosmolarity-induced HCECs with the Qishen prescription inhibits the JNK1/AQP5 pathway and reduces the expression levels of TNF-α, IL-6, and Caspase-1, thereby suppressing inflammation and apoptosis (Zhao et al., 2022a).

Therefore, *in vitro* studies show that the Qishen prescription inhibits DED-associated inflammation and apoptosis by suppressing the MAPK and JNK1/AQP5 pathways.

### 6.4 Runmu Ling granules

Runmu Ling granules (RMLG) are formulated from *Bidens pilosa* L. [Asteraceae; Bidentis herba] (spanish needles), *Lycium barbarum* L. [Solanaceae; lycii fructus] (barbary wolfberry fruit), and *Chrysanthemum × morifolium* (Ramat.) Hemsl. [Asteraceae; Chrysanthemi flos] (chrysanthemum) and are primarily used to nourish yin and clearing heat. RMLG significantly alleviated corneal injury and enhanced tear film stability/secretion in the SCOP-induced DED model rats. Treatment of BAC-induced HCECs with RMLG significantly improved cell viability. RMLG suppressed the expression levels of NLRP3, GSDMD, ASC, pro-Caspase-1, and cleaved-Caspase-1, and attenuated downstream IL-1β, IL-18, and TNF-α-related inflammation. These results indicate that pyroptosis is a critical pathogenic mechanisms in DED and is regulated by the NLRP3/GSDMD pathway. RMLG alleviates inflammation in DED by targeting pyroptosis and the NLRP3/GSDMD pathway (Luo et al., 2024).

In the BAC-induced DED model rabbits, nebulization of Runmu Ling Eye Drops (derived from RMLG) at 0.51 g/mL increased tear production, improved tear film stability, upregulated MUC5AC expression in the cornea and conjunctiva, reduced TNF-α and IL-6 levels in the lacrimal glands and aqueous humor, and decreased the levels of p-JNK/JNK, p-p38/p38, p-p65/p65, and MMP-9 in the cornea. This indicates that the Runmu Ling formula exerts anti-inflammatory and immunomodulatory effects by inhibiting the JNK/p38 pathway (Song et al., 2023). Ultrasonic nebulization for ocular delivery inherently lacks precise control over the critical parameters of dose and reproducibility due to unpredictable ocular deposition and variable animal response.

### 6.5 Modified formula of Danzhi Xiaoyao Powder

Danzhi Xiaoyao Powder is a classical prescription widely used in clinical practice for treating ophthalmic diseases associated with liver meridian heat stagnation syndrome (Ding et al., 2025).

Runmu Xiaoyao Powder is modified from Danzhi Xiaoyao Powder by excluding *M. canadensis* L. [Lamiaceae; menthae haplocalycis herba] (peppermint) and including *L. barbarum* L. [Solanaceae; lycii fructus] (barbary wolfberry fruit), *B. officinalis* Maxim. [Scrophulariaceae; buddlejae flos] (pale butterflybush flower), and *Chrysanthemum × morifolium* (Ramat.) Hemsl. [Asteraceae; Chrysanthemi flos] (chrysanthemum). DED model mice exhibiting liver meridian heat stagnation syndrome were established using BAC eye drops and chronic pain stimulation. Then, they were administered 7.25, 14.5, and 29 g/kg Runmu Xiaoyao Powder. The results showed that Runmu Xiaoyao Powder suppressed IRAK1/TRAF6/NF-κB signaling pathway by upregulating of miR-146a-5p in the mouse corneal and lacrimal gland tissues. The optimal effect was obtained with 29 g/kg Runmu Xiaoyao Powder, which effectively decreased the expression levels of MMPs, IL-1β, and TNF-α, reduced ocular surface tissue damage, enhanced tear secretion, and improved tear film stability (Liu T. et al., 2024).

Liu et al. developed the modified Danzhi Xiaoyao Powder (MDXP) through modification of the original formula of Danzhi Xiaoyao Powder by excluding *M. canadensis* L. [Lamiaceae; menthae haplocalycis herba] (peppermint) and *Zingiber officinale* Roscoe [Zingiberaceae; zingiberis rhizoma recens] (ginger), and adding *B. officinalis* Maxim. [Scrophulariaceae; buddlejae flos] (pale butterflybush flower) and *Chrysanthemum × morifolium* (Ramat.) Hemsl. [Asteraceae; Chrysanthemi flos] (chrysanthemum). *In vivo*, MDXP inhibited fcγR-mediated phagocytosis by downregulating CDC42, ARPC2, and ACTR3 levels, reduced inflammation by suppressing TNF-α and IL-1β levels, suppressed apoptosis, repaired corneal injury, enhanced tear secretion and tear film stability, and alleviated tension and anxiety. *In vitro*, MDXP also decreased TNF-α and IL-1β levels in HCECs under hyperosmotic conditions, as well as in LPS-stimulated RAW264.7 and THP-1 cells (Liu et al., 2024b).

### 6.6 Sihuang Qingling Liquid

Sihuang Qingling Liquid (SHQLY) is formulated with *P. sibiricum* Redouté [Asparagaceae; Polygonati rhizome] (solomonseal rhizome), *S. baicalensis* Georgi [Lamiaceae; scutellariae radix] (baical skullcap root), *C. chinensis* Franch. [Ranunculaceae; coptidis rhizoma] (Chinese goldthread), *Chrysanthemum × morifolium* (Ramat.) Hemsl. [Asteraceae; Chrysanthemi flos] (chrysanthemum), and *M. canadensis* L. [Lamiaceae; menthae haplocalycis herba] (peppermint). SHQLY is used in TCM for scattering wind and draining heat, improving vision and drying dampness, and nourishing yin and invigorating blood. Treatment of LPS-induced RAW264.7 cells with 10 or 20 μg/mL SHQLY demonstrated reduced secretion of IL-6 and TNF-α, decreased ROS as well as p-ERK, p-p38, p-JNK, p-IκBα, and p-p65 levels, and suppression of p65 nuclear translocation in a dose-dependent manner. This suggested that SHQLY alleviates DED-related inflammatory responses and oxidative damage by inhibiting the MAPK/NF-κB pathway (Chen et al., 2024).

### 6.7 Modified Siwei Dafa Powder

Siwei Dafa Powder is an ancient TCM ophthalmic prescription from “The Great Book of Ophthalmology.” This formula contains numerous acrid, dissipated, warm, and hot medicines and is primarily used for treating eye diseases caused by the stagnation of wind-cold-dampness (Chen and Chen, 2004). Modified Siwei Dafa Powder was developed by adding four additional metabolites—*Z. officinale* Roscoe [Zingiberaceae; zingiberis rhizoma recens] (ginger), Scrophularia ningpoensis Hemsl. [Scrophulariaceae; scrophulariae radix] (figwort root), *Rehmannia glutinosa* (Gaertn.) Libosch. ex DC. [Orobanchaceae; Rehmanniae radix] (rehmannia root), and *Ophiopogon japonicus* (Thunb.) Ker Gawl. [Asparagaceae; ophiopogonis radix] (dwarf lilyturf tuber)—to the original formulation. A rat model of DED with cold-dry syndrome was established using a low-temperature and low-humidity small animal adversity chamber in combination with desiccants. Treatment with Modified Siwei Dafa Powder (5.89 g/kg/d, oral) for 7 days improved clinical signs (tear secretion, BUT, corneal repair) and modulated apoptosis in lacrimal gland tissue by significantly downregulating Bax and Caspase-9 (with Caspase-3 showing a decreasing trend), upregulating Bcl-2, and reducing IL-6 (Chen L. et al., 2023).

### 6.8 Huashi Runjing Decoction

Clinical studies have demonstrated that the Huashi Runjing Decoction possesses multiple therapeutic properties, including dampness removal, liver nourishment, yin boosting, collaterals unblocking, ocular moisturization, and vision improvement. In the *in vivo* studies with castrated DED rats, Huashi Runjing Decoction exhibited androgen-like effects and suppressed the NF-κB signaling pathway by downregulating IKKα, IKKβ, IκB, and p65, and reducing the expression levels of TNF-α, IL-1α, and IL-6, thereby alleviating corneal and lacrimal gland damage, prolonging BUT, and enhancing tear secretion (Yang J. et al., 2023).

### 6.9 Zhenzhu Mingmu Eye Drops

The primary metabolites of Zhenzhu Mingmu Eye Drops (ZMED) are *Pinctada martensii* (Dunker) [Pteriidae; Margarita] (pearl) extract and *Camphora officinarum* Boerh. ex Fabr. [Lauraceae; borneolum] (natural borneol). The *P. martensii* (Dunker) [Pteriidae; Margarita] (pearl) extract is abundant in amino acids and exhibits anti-inflammatory and anti-apoptotic properties. In the *in vitro* experiments, a DED injury model was established by incubating HCECs with a 150 ng/mL solution of TNF-α and IFN-γ in a 1:1 ratio. Then, the treatment groups were incubated with different dilutions of ZMED (1,000x, 100x, and 50x). The survival rate of HCECs was significantly enhanced by 50X ZMED. Furthermore, ZMED dilutions below 100x reduced HCEC apoptosis in a concentration-dependent manner and mitigated G2 phase arrest by downregulating cleaved-Caspase-8, cleaved-Caspase-3, and PARP, upregulating Bcl-2 and Bax, and enhancing ERK1/2 phosphorylation (Wu et al., 2022). Notably, the inclusion of *C. officinarum* Boerh. ex Fabr. [Lauraceae; borneolum] (natural borneol), a known irritant, necessitates a thorough safety evaluation to determine a safe concentration that balances efficacy with ocular tolerance, ensuring it does not cause significant stinging, corneal damage, or inflammation upon short or long-term use.

### 6.10 Xiaosheng Granules

Xiaosheng Granules are formulated by integrating two classical TCM prescriptions, Xiaoyao Powder and Shengmai Powder, which possess therapeutic effects for soothing the liver and nourishing yin. In SCOP-induced DED mouse model, Xiaosheng Granules increased tear secretion, prolonged tear film BUT, improved corneal and lacrimal gland morphology, and reduced corneal epithelial apoptosis and expression of IL-1β, IL-6, TNF-α. Further *in vitro* mechanistic studies demonstrated that Xiaosheng Granules protected corneal epithelial cells by inhibiting PERK-eIF2α-ATF4-CHOP/Caspase-12 ER stress pathway and mitochondrial apoptotic pathway, and exerted anti-inflammatory effects in macrophages by suppressing MAPK/NF-κB signaling pathway (Du, 2018).

### 6.11 Erzhi Pills

Erzhi Pills consist of *Ligustrum lucidum* W.T.Aiton [Oleaceae; ligustri lucidi fructus] (glossy privet fruit) and *Eclipta prostrata* (L.) L. [Asteraceae; ecliptae herba] (yerbadetajo herb), and demonstrate yin nourishment and kidney tonification. A mouse model of DED was generated via subcutaneous injections of SCOP and administered oral doses of Erzhi Pills (3, 6, and 12 g/kg). After 14 days of treatment, significant improvements were observed in tear production and corneal fluorescein staining in the mice receiving 12 g/kg of Erzhi Pills. The treatment significantly reduced the expression levels of TNF-α, IL-1β, and IL-4, and inhibited the phosphorylation and nuclear translocation of p65. Therefore, Erzhi Pills alleviate DED ocular surface inflammation by suppressing NF-κB signaling (Shi X. et al., 2023).

### 6.12 Zhenshi Guben Liquid

Zhenshi Guben Liquid is a TCM formulation that demonstrates multiple therapeutic functions, including yin and liver nourishment, and promotes body fluid production and kidney functions. A network pharmacology-based analysis predicted that Zhenshi Guben Liquid acts on DED through multiple active metabolites (e.g., paeoniflorin, aloe-emodin, quercetin) and targets (including IL-6, CASP3, IL-1β, and MMP-9), with significant enrichment in the TNF signaling pathway. These predictions were experimentally validated in a DED mouse model induced by SCOP and dry environment exposure. Administration of Zhenshi Guben Liquid at 103.84 g/kg/d significantly increased tear secretion, prolonged tear film BUT, and decreased corneal/lacrimal gland damage. It also downregulated expression levels of IL-1β, IL-6, MMP-9, Caspase-3, and TNF-α. Furthermore, both 51.92 and 103.84 g/kg/d doses of Zhenshi Guben Liquid reduced the expression of TNF-α, IKK, and p65, and inhibited the phosphorylation of IKK, IκBα, and p65. These findings confirm the network pharmacology predictions and suggest that Zhenshi Guben Liquid alleviates ocular surface inflammation by suppressing the TNF-α-mediated IKK/IκB/NF-κB signaling pathway (Dong, 2024).

## 7 Discussion and conclusion

The prevalence of DED has risen globally. In China, the prevalence of DED ranges from 21.0% to 50.1% (Ophthalmology Branch of Shanghai Association of Chinese Medicine, 2025). Prolonged use of electronic devices, which reduces blinking frequency and accelerates tear evaporation, has significantly increased the incidence of DED among young individuals, especially in students and office workers. The pathological mechanisms underlying DED are complex and multi-faceted, including tear film instability, inflammation, oxidative stress, and nerve injury. Therefore, abrogating the vicious cycle of “tear film instability-tear hyperosmolarity-inflammation-apoptosis” is challenging. Current mainstream pharmacological management for DED are based on existing pathological mechanisms and have several limitations. The clinically used artificial tears must be carefully selected according to their components. High-concentration surfactants required for dissolving oily agents may exhibit ophthalmic toxicity and preservatives in some formulations may exacerbate ocular surface injury (Labetoulle et al., 2022). Immunomodulators such as cyclosporine A have a slow onset of action (requiring several weeks to months) and may cause ocular surface irritation (McCann et al., 2024). Physical therapies such as LipiFlow, iLux, and TearCare are time-consuming and are associated with uncertain treatment efficacy, poor patient compliance, and high costs (Beining et al., 2022). However, the management of multifactorial DED can be challenging. The TCM paradigm, employing complex botanical formulas, represents a distinct therapeutic strategy aimed at simultaneously modulate multiple nodes within the disease network. This multi-target approach could offer a complementary strategy to conventional management, which may involve a combination of therapies to address different aspects of the disease.

In contrast, the holistic and multi-target nature of TCM offers a unique advantage. In TCM, DED is categorized under “white xerotic syndrome” and “divine water about to dry up,” with a long history of treatment guided by the core principle of “treatment based on syndrome differentiation”. This principle aligns with the modern, etiology-driven management approach for DED, as reflected in the TFOS DEWS III (2025) subclassification. For instance, DED primarily driven by aqueous/tear deficiency often corresponds to TCM patterns of Yin deficiency, where therapies aimed at nourishing Yin and promoting fluid secretion—such as Qiju Dihuang Pills, Erzhi Pills, and metabolites like puerarin—may be particularly beneficial. Conversely, DED linked to meibomian gland dysfunction and evaporative stress frequently corresponds to patterns of internal heat or damp-heat, potentially responding better to TCM interventions with heat-clearing and detoxifying properties, such as formulas containing *S. baicalensis* Georgi [Lamiaceae; scutellariae radix] (baical skullcap root) or metabolites like berberine. The multi-component, multi-target approach of TCM holds potential for managing DED with multiple coexisting etiologies, as it can simultaneously address combined pathological drivers like inflammation, oxidative stress, and tissue deficiency through synergistic actions. Modern research has begun to elucidate how plant-derived metabolites, botanical drug extracts, and TCM formulas synergistically intervene through multiple signaling pathways (e.g., PI3K/AKT, MAPK, NF-κB, NLRP3, Nrf2), forming a coordinated network effect that can disrupt the vicious cycle. To transparently present this multi-level preclinical evidence, the experimental findings summarized in this review are systematically categorized into *in vitro* mechanistic studies and *in vivo* pharmacological studies across dedicated tables. The separation of evidence into Table 2 (*in vivo*) and Table 3 (*in vitro*) provides a clear framework for assessing the translational landscape. This structure helps identify gaps, such as pharmacological outcomes observed *in vivo* that lack mechanistic explanation *in vitro*, and pathway modulations identified *in vitro* that lack corresponding *in vivo* validation. Table 1 synthesizes these observations, highlighting pathways with more convergent evidence.

However, the clinical application of this customized approach is currently limited by a lack of high-quality evidence. While classic prescriptions have shown beneficial effects in preclinical models, their specific mechanisms and pharmacological effects relative to specific DED subtypes remain inadequately characterized. Moreover, as detailed in the following section, several important limitations in the existing evidence must be acknowledged and addressed.

This article reviews the present knowledge regarding TCM interventions investigated in preclinical DED models and their underlying signaling pathways. Plant-derived metabolites, botanical drug extracts, and TCM formulas for DED have significant advantages and are novel and promising avenues for the future management of DED because of their multi-component, multi-target, and multi-pathway approach. To translate this potential into therapeutic reality, a concerted effort to address existing research gaps is imperative, as outlined below.

## 8 Limitations and future perspectives

### 8.1 The challenge of Pan-Assay interference compounds (PAINS) in interpreting preclinical data

A critical methodological consideration for this review is the presence of Pan-Assay Interference Compounds (PAINS) among the metabolites discussed. PAINS are chemical compounds that frequently produce false-positive results in high-throughput screening assays by non-specifically interacting with multiple biological targets rather than through selective, mechanism-based binding (Bolz et al., 2021). Computational approaches have been developed to identify such compounds based on their structural properties (Magalhães et al., 2021). Common structural classes of PAINS include flavonoids, polyphenols, quinones, and certain alkaloids. Many of the active metabolites covered in this review, including quercetin, curcumin, resveratrol, epigallocatechin gallate (EGCG), and berberine, have been discussed in the literature as possessing structural features associated with PAINS activity. This characteristic necessitates cautious interpretation of the *in vitro* data summarized herein. For these compounds, some observed effects on inflammatory cytokines, oxidative stress markers, and signaling pathways may partially reflect non-specific interactions rather than genuine target engagement. Consequently, the pharmacological relevance of findings derived solely from *in vitro* assays cannot be assumed without additional validation.

However, several factors support the continued scientific value of the evidence reviewed. First, the evidence base for many interventions is multi-layered, extending beyond *in vitro* assays to include *in vivo* animal models of DED. While *in vivo* effects may still involve complex mechanisms, the observation that these compounds consistently ameliorate disease-relevant endpoints–such as increased tear secretion, reduced corneal damage, and preserved goblet cell density–provides functional validation of their biological activity at the whole-organism level. The protective effects demonstrated in these models, while requiring confirmation in humans, represent genuine biological activity rather than purely artifactual interference. Second, the multi-component, multi-target paradigm of TCM may inherently differ from the single-target specificity sought in conventional drug discovery. Within a complex TCM formula, multiple constituents–some of which may be PAINS–can act synergistically to modulate interconnected pathological networks. The binding characteristics of such compounds, when contextualized within a polypharmacological framework, may contribute to the holistic regulatory effects observed with TCM formulas. This does not diminish the need for rigorous validation, but it suggests that dismissing these compounds entirely would overlook their potential role within complex traditional interventions. Third, an increasing number of studies are employing mechanistic validation strategies that strengthen causal inferences. These include the use of specific pharmacological inhibitors (e.g., SB203580 for p38 MAPK, LY294002 for PI3K), genetic approaches (e.g., siRNA knockdown of proposed targets such as SIRT1 or Nrf2), and structural analogs with modified activity. Such approaches help distinguish genuine pathway modulation from non-specific interference.

This issue has been insufficiently addressed in the existing literature. Future studies investigating TCM-derived metabolites in DED should incorporate rigorous counter-screens to exclude PAINS-related artifacts, structure-activity relationship (SAR) studies using non-PAINS analogs where possible, target engagement validation through biophysical methods in addition to functional readouts, and an emphasis on *in vivo* pharmacokinetic-pharmacodynamic (PK-PD) relationships to establish that observed effects occur at physiologically achievable concentrations. By integrating these approaches, the field can move toward a more robust understanding of how TCM-derived agents exert their biological effects, distinguishing genuine pharmacological mechanisms from assay interference. The present review synthesizes current evidence with this critical perspective in mind.

### 8.2 Other limitations and future directions

This comprehensive review synthesizes substantial evidence supporting multi-target effects of TCM in DED; however, the findings must be interpreted within the context of several overarching limitations inherent in the current literature. A key interpretative challenge, evident from the data separation in Tables 2, 3, is the frequent disconnect between study types. For many interventions, evidence is predominantly derived from either *in vivo* or *in vitro* models, but not both, complicating the establishment of definitive mechanistic links to pharmacological effects. For many interventions, the proposed regulation of a specific signaling pathway is primarily established in *in vitro* models, while the accompanying *in vivo* studies may only assess downstream phenotypic outcomes without direct validation of the pathway itself. This gap complicates the definitive attribution of observed beneficial effects to particular molecular mechanisms. The most significant constraint is the predominance of preclinical evidence, with conclusions primarily based on *in vitro* and animal models that may not fully replicate the complexity and heterogeneity of human DED. Furthermore, a critical analysis of the included studies reveals that methodological weaknesses were noted in some investigations, and the broad scope of this review precluded an in-depth analysis of every study for each individual formula. Among the issues identified in specific studies were insufficient sample sizes, lack of randomization and blinding procedures, incomplete botanical and extraction characterization, variability in the preparation and chemical definition of TCM agents (a challenge systematically documented in Supplementary Tables S2, S3), which complicates reproducibility and cross-study comparison, and limited dose-response evaluations. These shortcomings compromise the reliability and generalizability of the findings.

A particularly notable gap is the widespread neglect of systematic safety profiling. Only a minority of studies evaluated potential cytotoxic effects or long-term toxicity, which is concerning given that some TCM metabolites (e.g., alkaloids like berberine) have known potential for toxicity. Finally, and most critically, clinical evidence remains scarce. None of the reviewed formulations have undergone robust Phase I/II clinical trials to establish foundational human safety, tolerability, and preliminary efficacy, representing the single greatest barrier to clinical translation.

To overcome these limitations, future research should be strategically directed along several interconnected paths. The initial phase should leverage network pharmacology and systems biology to pinpoint active metabolites and promising formula combinations, whose pharmacological effects and multi-target mechanisms must then be rigorously validated in standardized preclinical models. Concurrently, enhancing TCM standardization through modern chemical profiling is a prerequisite for reproducibility, while the development of novel formulations (e.g., sustained-release eye drops, nanocarriers) is crucial to improve ocular bioavailability. Mechanistic exploration should be deepened using multi-omics and genetic tools, and should be expanded to investigate emerging areas such as the interaction between TCM and the ocular surface microbiome, implemented alongside comprehensive safety assessments. The ultimate and most important step is the conduct of high-quality, multicenter randomized controlled trials (RCTs) to establish definitive evidence of clinical efficacy and safety. A forward-looking priority for these clinical trials is the investigation of personalized approaches stratified by DED subtypes, aligning TCM’s holistic philosophy with the rigorous standards of evidence-based medicine to develop the next-generation of therapies.

While TCM offers a holistic and multi-target advantage for managing DED’s complex pathology, addressing these limitations through more rigorous, standardized, and translational research is paramount for its successful integration into mainstream evidence-based ophthalmological practice.

## Glossary

8-OHdG: 8-hydroxy-2′-deoxyguanosine
ACSL4: Acyl-CoA synthetase long-chain family 4
ACTR3: Actin-related protein 3
AKT (PKB): Protein kinase B
AMPK: AMP-activated protein kinase/Adenosine monophosphate-activated protein kinase
ANOVA: Analysis of variance
AO: Aurantio-obtusin
APAF-1: Apoptotic protease activating factor 1
APCs: Antigen-presenting cells
AQP5: Aquaporin 5
AQPs: Aquaporins
AREs: Antioxidant response elements
ARPC2: Actin-related protein 2/3 complex subunit 2
ASC: Apoptosis-associated speck-like protein
AST: Astaxanthin
ATF4: Activating transcription factor 4
ATG: Autophagy-related
ATM: Ataxia-telangiectasia mutated
BAC: Benzalkonium chloride
BAK1: Bcl-2 antagonist/killer 1
Bax: Bcl-2-associated X protein
BBR: Berberine
Bcl-2: B-cell lymphoma/leukemia-2
Bcl-xl: B-cell lymphoma-extra-large
BID: BH3 interaction domain death agonist
CBA: Cytometric Bead Array
CAT: Catalase
CCL2: C-C motif ligand 2
CDC42: Cell division control protein 42
CG: Carboxyl-chitosan modified graphene
CGA: Chlorogenic acid
CHOP: C/EBP homologous protein
COX-2: Cyclooxygenase-2
CQ: Chloroquine
CREB: cAMP-response element binding protein
CSL: CBF-1/suppressor of hairless/Lag1
CT: Cytoplasmic tail
Cul3: Cullin-3
CXCL10: C-X-C motif ligand 10
Cyt C: Cytochrome C
DAMP: Damage-associated molecular pattern
DCs: Dendritic cells
DED: Dry eye disease
DISC: Death-inducing signaling complex
DRAM-1: Damage-regulated autophagy modulator
EDE: Experimental dry eye
EGCG: Epigallocatechin gallate
EGFR: Epidermal growth factor receptor
eIF2α: Eukaryotic initiation factor 2α
ELISA: Enzyme-linked immunosorbent assay
EPM: Elevated plus maze
ER: Endoplasmic reticulum
ERK: Extracellular signal-regulated kinase
EVO: Evodiamine
FADD: Fas-Associated Death Domain protein
FC: Flow cytometry
FcγR: Fc gamma receptor
FL: Fluorescein staining
Fox: Forkhead box proteins
GA: Gallic acid
GAS: γ-activating sequences
GCD: Goblet cell density
GCLM: Glutamate-cysteine ligase modifier subunit
GPCR: G protein-coupled receptor
GPX4: Glutathione peroxidase 4
GR: Glutathione reductase
GSDMD: Gasdermin D
GSH: Glutathione
GSTP1: Glutathione S-transferase P1
HCE-2: Human corneal epithelial-2
HCECs: Human corneal epithelial cells
HCE-T: Human corneal epithelial cell-transformed
HE: Hematoxylin and eosin (staining)
HES: Hairy/enhancer of split
HEY: Hairy/enhancer of split related to YRPW motif
HIF-1α: Hypoxia-inducible factor-1α
HKs: Human cornea keratocytes
HMOX1: Heme oxygenase-1
HO-1: Heme oxygenase-1
ICAM-1: Intercellular adhesion molecule-1
IFN: Interferon
IF: Immunofluorescence
iHCECs: Immortalized human corneal epithelial cells
IHC: Immunohistochemistry
i.m.: Intramuscular injection
IκB: Inhibitor of κB
IL: Interleukin
iNOS: Inducible nitric oxide synthase
IRAK1: Interleukin-1 receptor-associated kinase 1
IRFs: Interferon regulatory factors
ISGs: Interferon-stimulated genes
ISRE: Interferon-stimulated response elements
JAK: Janus kinase
JNK: c-Jun N-terminal kinase
Keap1: Kelch-like ECH-associated protein 1
LC3: Microtubule-associated protein 1A/1B-light chain 3
LDH: Lactate dehydrogenase
LFU: Lacrimal functional unit
LG: Lacrimal gland
LGECs: Lacrimal gland epithelial cells
LKB1: Liver kinase B1
LPS: Lipopolysaccharide
MAPK: Mitogen-activated protein kinase
MAPKK: MAPK kinase
MAPKKK: MAPK kinase kinase
MCP-1: Monocyte chemoattractant protein-1
MDA: Malondialdehyde
MDXP: Modified Danzhi Xiaoyao Powder
METC: Mitochondrial electron transport chain
MG: Meibomian gland
MGD: Meibomian gland dysfunction
MMP: Matrix metalloproteinase
MOMP: Mitochondrial outer membrane permeability
MPTPs: Mitochondrial permeability transition pores
MSK1: Mitogen- and stress-activated protein kinase 1
mTOR: Mechanistic (mammalian) target of rapamycin
MUC: Mucin
MyD88: Myeloid differentiation primary response protein 88
**NAD** ^**+**^: Nicotinamide adenine dinucleotide
NF-κB: Nuclear factor kappa B
NICD: Notch intracellular domain
NIK: NF-κB-inducing kinase
NLRP3: Nod-like receptor protein 3
NO: Nitric oxide
Notch: Notch receptor
NQO1: NAD(P)H:quinone oxidoreductase 1
Nrf2: Nuclear factor erythroid 2-related factor 2
NTMD: Notch transmembrane domain
OA: Oroxylin A
OFT: Open field test
OGD: Oregon Green Dextran
p38 MAPK: p38 mitogen-activated protein kinase
p53: Tumor protein p53
p62 (SQSTM1): Sequestosome-1
PAINS: Pan-Assay Interference Compounds
PARP: Poly (ADP-ribose) polymerase
PAS: Periodic acid-Schiff (staining)
PBS: Phosphate-buffered saline
PCE: Polygonum cuspidatum extract
PD: Polydatin
PDK1: Phosphoinositide-dependent protein kinase 1
PERK: Protein kinase R-like endoplasmic reticulum kinase
PI3K: Phosphatidylinositol 3-kinase
PKA: Protein kinase A
PK-PD: pharmacokinetic-pharmacodynamic
priHCECs: Primary human corneal epithelial cells
PS: Pterostilbene
PS-GA-RGD: Pterostilbene-glutaricanhydride-arginine-glycine-aspartic acid
PTEN: Phosphatase and tensin homolog
qPCR: Quantitative Polymerase Chain Reaction
QXRMY: Qingxuan Runmu Decoction
RAW 264.7: Mouse leukemic monocyte macrophage cell line
Rbx1: RING box protein-1
RES: Resveratrol
RMLG: Runmu Ling granules
RORγt: Retinoid-related orphan receptor γt
ROS: Reactive oxygen species
RTK: Receptor tyrosine kinase
RT-qPCR (qRT-PCR): Reverse Transcription Quantitative Polymerase Chain Reaction
SAR: structure-activity relationship
SCOP: Scopolamine (hydrobromide)
SIRT: Sirtuin
SIT: Schirmer’s test
sMaf: Small musculoaponeurotic fibrosarcoma Maf
SM934: β-aminoarteether maleate
SOD: Superoxide dismutase
STAT: Signal transducer and activator of transcription
TAK1: TGF-β activated kinase 1
TBUT: Tear film break-up time
TCM: Traditional Chinese medicine
TEM: Transmission electron microscopy
TGF-β: Transforming growth factor-beta
TH: T helper cell
THO: Tear hyperosmolarity
THP-1: Human acute monocytic leukemia cell line
TLR4: Toll-like receptor 4
TNF-α: Tumor necrosis factor-alpha
TRAF6: TNF receptor associated factor 6
Treg: Regulatory T cell
TUNEL: Terminal deoxynucleotidyl transferase dUTP nick end labeling
ULK1: UNC-51-like kinase 1
VDR: Vitamin D receptor
VEGF: Vascular endothelial growth factor
VEGFR: Vascular endothelial growth factor receptor
WB: Western blotting
ZMED: Zhenzhu Mingmu Eye Drops
ZO-1: Zonula occludens-1
