# Supplementary material for: Managing dry eye disease – a review of selected traditional Chinese medicine and some of their metabolites focusing on molecular mechanisms and signaling pathways
Source: Front Pharmacol. 2026 Mar 31;17:1693198. doi: 10.3389/fphar.2026.1693198 (PMC13076346; doi:10.3389/fphar.2026.1693198)
Supplement: Supplementary file 1 [file Supplementaryfile1.docx]

Date of Search: [April 23, 2025]

1. PubMed

Search Query:

(signaling pathway) AND (dry eye)AND( (chinese medicine)OR(Chinese herbal monomer)OR(Chinese herbal extract)OR(herbal formula))

Records Retrieved: [67]

2. Web of Science

Search Query:

TS=(signaling pathway) AND TS=(dry eye)AND( TS= ( chinese medicine)OR TS=(Chinese herbal monomer) OR TS=(Chinese herbal extract)OR TS=(herbal formula))

Records Retrieved: [7]

3. Embase

Search Query:

('signaling'/exp OR signaling) AND ('pathway'/exp OR pathway) AND dry AND ('eye'/exp OR eye) AND (('chinese'/exp OR chinese) AND ('medicine'/exp OR medicine) OR (('chinese'/exp OR chinese) AND herbal AND ('monomer'/exp OR monomer)) OR (('chinese'/exp OR chinese) AND herbal AND ('extract'/exp OR extract)) OR (herbal AND ('formula'/exp OR formula)))

Records Retrieved: [51]

4. SinoMed

Search Query:

"干眼"[常用字段:智能] AND "信号通路"[常用字段:智能] AND( "中药"[常用字段:智能] OR "中药单体"[常用字段:智能] OR "中药提取物"[常用字段:智能] OR "中药复方"[常用字段:智能])

Records Retrieved: [14]

5. CNKI

Search Query:

SU='干眼' AND FT='信号通路' AND (FT='中药' OR FT='中药单体' OR FT='中药提取物' OR FT='中药复方')

Records Retrieved: [374]

6. Wanfang

Search Query:

主题:(干眼) and 主题:(信号通路) and( 主题:(中药) or 主题:(中药单体) or 主题:(中药提取物) or(主题:(中药复方))

Records Retrieved: [152]

7. VIP

Search Query:

U=干眼 AND U=信号通路 AND (U=中药 OR U=中药单体 OR U=中药提取物 OR U=中药复方)

Records Retrieved: [26]
