# Supplementary material for: Managing dry eye disease – a review of selected traditional Chinese medicine and some of their metabolites focusing on molecular mechanisms and signaling pathways
Source: Front Pharmacol. 2026 Mar 31;17:1693198. doi: 10.3389/fphar.2026.1693198 (PMC13076346; doi:10.3389/fphar.2026.1693198)
Supplement: Supplementary file 2 [file Table1.docx]

**Supplementary Table S1.** Detailed Evidence by Signaling Pathway for TCM Interventions in DED.

| **Pathway^a^** | **Ref.** | **Study Type** | **TCM Intervention Tested** | **Key Pathway-Related Finding^b^** | **Evidence Quality^c^** | **Experimental Model** | **Key Concentration / Dose^d^** |
| --- | --- | --- | --- | --- | --- | --- | --- |
| NF-κB Pathway Inhibition | (Li, 2020) | *In vivo* | Berberine | - ↓ p-IKK, p-NF-κB, p-IκBα (phosphorylation) | Direct | Mouse (SCOP+DS) | 2 mg/mL (topical) |
| NF-κB Pathway Inhibition | (Wu, 2019) | *In vivo* | Paeonol | - ↓ p-NF-κB (phosphorylation) | Direct | Mouse (SCOP+DS) | 1%, 5%, 10% (topical) |
| NF-κB Pathway Inhibition | (Yim et al., 2022) | *In vivo* | KIOM-2015E | - ↓ p-p65 (phosphorylation) | Direct | Mouse (BAC) | 0.5, 1 mg/mL (topical); 100 mg/kg (oral) |
| NF-κB Pathway Inhibition | (Ling et al., 2022) | *In vivo* | Dendrobium water extracts | - ↓ p-p65 (phosphorylation) | Direct | Rat (SCOP) | 200 mg/kg (oral) |
| NF-κB Pathway Inhibition | (P. Liu et al., 2024a) | *In vivo* | Linarin | - ↓ p65 (protein) | Direct | Mouse (BAC+chronic pain/tail clamping) | 12.5, 25, 50 mg/kg (oral) |
| NF-κB Pathway Inhibition | (Zhu et al., 2024) | *In vivo* | Aurantio-obtusin | - ↓ p-p65, p-IKKβ, p-IκBα (phosphorylation) | Direct | Rat (BAC) | 0.5% (topical) |
| NF-κB Pathway Inhibition | (Yang et al., 2021) | *In vivo* | SM934 | - ↓ TLR4, MyD88 (protein) | Direct | Rat/Mouse (SCOP/BAC) | 0.1%, 0.5% (topical) |
| NF-κB Pathway Inhibition | (Yi Han et al., 2023) | *In vivo* | Berberine | - ↓ p-IKKα/β, p-NF-κB (phosphorylation) | Direct | Mouse (SCOP+DS) | 0.5, 2 mg/mL (topical) |
| NF-κB Pathway Inhibition | (Zhao et al., 2024b) | *In vivo* | Qingxuan Runmu Decoction | - ↓ p-p65 (phosphorylation) | Direct | SD rat (BAC) | 0.6 g/mL (gavage) |
| NF-κB Pathway Inhibition | (Song et al., 2023) | *In vivo* | Runmu Ling Eye Drops | - ↓ p-p65/p65 (phosphorylation ratio) | Direct | New Zealand rabbit (BAC) | 0.51 g/mL (topical) |
| NF-κB Pathway Inhibition | (Liu T. et al., 2024) | *In vivo* | Runmu Xiaoyao Powder | - ↓ p-p65 (phosphorylation) - ↓ p65 (protein, mRNA) | Direct | C57BL/6J mouse (BAC+pain) | 7.25, 14.5, 29 g/kg (gavage) |
| NF-κB Pathway Inhibition | (Yang J. et al., 2023) | *In vivo* | Huashi Runjing Decoction | - ↓ IKKα, IKKβ, IκB, p65 (protein) | Direct | SD rat (bilateral orchiectomy) | 0.93 g/mL (gavage) |
| NF-κB Pathway Inhibition | (Shi X. et al., 2023) | *In vivo* | Erzhi Pills | - ↓ p-p65 (phosphorylation) | Direct | C57BL/6 mouse (SCOP) | 12 g/kg (gavage) |

**Supplementary Table S1 (Continued).** Detailed Evidence by Signaling Pathway for TCM Interventions in DED.

| **Pathway^a^** | **Ref.** | **Study Type** | **TCM Intervention Tested** | **Key Pathway-Related Finding^b^** | **Evidence Quality^c^** | **Experimental Model** | **Key Concentration / Dose^d^** |
| --- | --- | --- | --- | --- | --- | --- | --- |
| NF-κB Pathway Inhibition | (Dong, 2024) | *In vivo* | Zhenshi Guben Liquid | - ↓ p-IKK, p-IκBα, p-p65 (phosphorylation) - ↓ IKK, p65 (mRNA) - ↑ IκBα (mRNA) | Direct | BALB/c mouse (SCOP+dry environment) | 25.96, 51.92, 103.84 g/kg (gavage) |
| NF-κB Pathway Inhibition | (Park et al., 2018) | *In vivo* | PCE Aqueous Extract | - ↓ TNF-α, IL-6, MMP9 (mRNA) - ↑ MUC4 (protein, mRNA) | Indirect | Rat (LG excision) | 10, 100, 250 mg/kg (oral) |
| NF-κB Pathway Inhibition | (Zhao et al., 2019) | *In vivo* | Paeoniflorin | - ↓ IL-1 (protein) | Indirect | Mouse (hyperosmotic saline) | 0.01%, 0.1%, 1% (topical) |
| NF-κB Pathway Inhibition | (Park et al., 2019) | *In vivo* | Polydatin | - ↓ TNF-α, IL-6, IL-1β, IFN-γ (mRNA) | Indirect | Rat (LG excision) | 0.05%, 0.5% (topical) |
| NF-κB Pathway Inhibition | (Li, 2023) | *In vivo* | PS-GA-RGD (Nano) | - ↓ IL-6, IL-1β (protein) | Indirect | Mouse (SCOP+dry environment) | 5 mg/mL (topical) |
| NF-κB Pathway Inhibition | (Li et al., 2023) | *In vivo* | Gallic Acid | - ↓ IL-6, IL-1β (protein) | Indirect | Mouse (SCOP+dry environment) | 5 mg/mL (topical) |
| NF-κB Pathway Inhibition | (Du, 2018) | *In vivo* | Xiaosheng Granules | - ↓ IL-1β, IL-6, TNF-α (protein) | Indirect | C57BL/6 mouse (SCOP) | 3.6 mg/g (gavage) |
| NF-κB Pathway Inhibition | (Chen et al., 2010) | *In vitro* | Curcumin | - ↓ nuclear p65 (protein) | Direct | HCECs (hyperosmolarity) | 5 µM |
| NF-κB Pathway Inhibition | (Park et al., 2018) | *In vitro* | PCE Aqueous Extract | - ↓ p-p65 (phosphorylation) | Direct | HCECs (hyperosmolarity) | 1, 10, 100 µg/mL |
| NF-κB Pathway Inhibition | (Chao et al., 2016) | *In vitro* | Lutein | - ↓ NF-κB (protein) | Direct | HCECs (hyperosmolarity) | 1, 3, 10 µM |
| NF-κB Pathway Inhibition | (Kim et al., 2018) | *In vitro* | KIOM-2015EW | - ↓ p65 (nuclear translocation) - ↓ p-IκB-α (phosphorylation) | Direct | HCECs (hyperosmolarity) | 0.05, 0.1, 0.2 mg/mL |
| NF-κB Pathway Inhibition | (Park et al., 2019) | *In vitro* | Polydatin | - ↓ p-p65 (phosphorylation) - ↓ nuclear p65 (protein) | Direct | Human conjunctival cells (hyperosmolarity) | 0.1, 1, 10 µM |

**Supplementary Table S1 (Continued).** Detailed Evidence by Signaling Pathway for TCM Interventions in DED.

| **Pathway^a^** | **Ref.** | **Study Type** | **TCM Intervention Tested** | **Key Pathway-Related Finding^b^** | **Evidence Quality^c^** | **Experimental Model** | **Key Concentration / Dose^d^** |
| --- | --- | --- | --- | --- | --- | --- | --- |
| NF-κB Pathway Inhibition | (Yang et al., 2021) | *In vitro* | SM934 | - ↓ p‑NF‑κB (phosphorylation) | Direct | RAW 264.7 (LPS) | 10 µM |
| NF-κB Pathway Inhibition | (Li, 2023) | *In vitro* | PS-GA-RGD (Nano) | - ↓ p-P65, p-IκBα (phosphorylation) | Direct | RAW 264.7 (LPS) | 10 µM |
| NF-κB Pathway Inhibition | (Li et al., 2023) | *In vitro* | Gallic Acid | - ↓ p-p65, p-IκBα (phosphorylation) | Direct | HCECs (hyperosmolarity); RAW 264.7 (LPS) | 100 µM |
| NF-κB Pathway Inhibition | (Yi Han et al., 2023) | *In vitro* | Berberine | - ↓ NF-κB nuclear translocation | Direct | HCECs (hyperosmolarity) | 2.5, 5 µM |
| NF-κB Pathway Inhibition | (Chen et al., 2024) | *In vitro* | Sihuang Qingling Liquid | - ↓ NF-κB nuclear translocation - ↓ p-p65, p-IκBα (phosphorylation) | Direct | RAW 264.7 (LPS) | 10, 20 µg/mL |
| NF-κB Pathway Inhibition | (Du, 2018) | *In vitro* | Xiaosheng Granules | - ↓ p-IKKβ, p-p65 (phosphorylation) - ↑ IκBα (protein) | Direct | RAW264.7 (hyperosmolarity/cytokines) | 100, 400 µg/mL |
| NF-κB Pathway Inhibition | (P. Liu et al., 2024a) | *In vitro* | Linarin | - ↓ TNF-α, IL-1β (protein) | Indirect | HCECs (hyperosmolarity) | 15 µM |
| NF-κB Pathway Inhibition | (Zhao et al., 2019) | *In vitro* | Paeoniflorin | - ↓ NF-κB (protein, mRNA) | Indirect | HCECs (hyperosmolarity) | 0.01%, 0.1%, 1% |
| NLRP3 Inflammasome Inhibition | (X. Li et al., 2024) | *In vivo* | Oridonin | - ↓ NLRP3, Caspase-1, N-GSDMD (protein) | Direct | Mouse (BAC) | 0.01%, 0.1%, 1% (topical) |
| NLRP3 Inflammasome Inhibition | (Wu et al., 2024) | *In vivo* | Huji Sheng Eye Drop | - ↓ NLRP3, Caspase-1 (mRNA, protein) | Direct | Mouse (BAC) | 0.005% (topical) |
| NLRP3 Inflammasome Inhibition | (Zhu et al., 2024) | *In vivo* | Aurantio-obtusin | - ↓ NLRP3, ASC, cleaved Caspase-1 (protein) | Direct | Rat (BAC) | 0.5% (topical) |
| NLRP3 Inflammasome Inhibition | (Park et al., 2019) | *In vivo* | Polydatin | - ↓ NLRP3 (protein, mRNA) | Direct | Rat (LG excision) | 0.05%, 0.5% (topical) |

**Supplementary Table S1 (Continued).** Detailed Evidence by Signaling Pathway for TCM Interventions in DED.

| **Pathway^a^** | **Ref.** | **Study Type** | **TCM Intervention Tested** | **Key Pathway-Related Finding^b^** | **Evidence Quality^c^** | **Experimental Model** | **Key Concentration / Dose^d^** |
| --- | --- | --- | --- | --- | --- | --- | --- |
| NLRP3 Inflammasome Inhibition | (Yang et al., 2021) | *In vivo* | SM934 | - ↓ NLRP3 (protein, mRNA) - ↓ ASC, cleaved caspase‑1 (protein) - ↓ Caspase‑1 (mRNA) | Direct | Rat/Mouse (SCOP/BAC) | 0.1%, 0.5% (topical) |
| NLRP3 Inflammasome Inhibition | (Luo et al., 2024) | *In vivo* | Runmu Ling granules | - ↓ NLRP3, GSDMD, ASC, pro-Caspase-1, cleaved Caspase-1 (protein) | Direct | SD rat (SCOP) | 0.75, 1.5 g/kg (gavage) |
| NLRP3 Inflammasome Inhibition | (X. Li et al., 2024) | *In vitro* | Oridonin | - ↓ NLRP3, Caspase-1, N-GSDMD (protein) - ↓ NLRP3, Caspase-1 (mRNA) | Direct | HCE-T cells (hyperosmolarity) | 2, 4 µM |
| NLRP3 Inflammasome Inhibition | (X. Liu et al., 2025) | *In vitro* | Oroxylin A | - ↓ NLRP3, Caspase-1 (protein, mRNA) - ↓ N-GSDMD, cleaved Caspase-1 (protein) - ↓ GSDMD (mRNA) | Direct | HCECs (hyperosmolarity) | 8, 10 µg/mL |
| NLRP3 Inflammasome Inhibition | (Park et al., 2019) | *In vitro* | Polydatin | - ↓ NLRP3, cleaved Caspase-1 (protein) | Direct | Human conjunctival cells (hyperosmolarity) | 0.1, 1, 10 µM |
| NLRP3 Inflammasome Inhibition | (Yang et al., 2021) | *In vitro* | SM934 | - ↓ NLRP3, ASC, cleaved Caspase‑1 (protein) | Direct | RAW 264.7 (LPS) | 10 µM |
| NLRP3 Inflammasome Inhibition | (Zhou et al., 2020) | *In vitro* | Genistein | - ↓ NLRP3, Caspase-1 (mRNA) | Direct | iHCECs/priHCECs (hyperosmolarity) | 50 µmol/L |
| NLRP3 Inflammasome Inhibition | (Luo et al., 2024) | *In vitro* | Runmu Ling granules | - ↓ NLRP3, GSDMD, ASC, pro-Caspase-1, cleaved Caspase-1 (protein) | Direct | HCECs (BAC) | 2.5%, 5%, 10% medicated serum |
| NLRP3 Inflammasome Inhibition | (Dong et al., 2024) | *In vitro* | Puerarin | - ↓ NLRP3 (protein) | Indirect | HCE-2 cells (hyperosmolarity) | 10, 30, 50 µM |
| MAPK Pathway Activation | (Cao et al., 2024) | *In vivo* | Polydatin | - ↑ p-MEK1/2/MEK1/2, p-ERK1/2/ERK1/2 (phosphorylation ratio) | Direct | Rat (SCOP) | 0.05%, 0.5% (topical) |

**Supplementary Table S1 (Continued).** Detailed Evidence by Signaling Pathway for TCM Interventions in DED.

| **Pathway^a^** | **Ref.** | **Study Type** | **TCM Intervention Tested** | **Key Pathway-Related Finding^b^** | **Evidence Quality^c^** | **Experimental Model** | **Key Concentration / Dose^d^** |
| --- | --- | --- | --- | --- | --- | --- | --- |
| MAPK Pathway Inhibition | (Ling et al., 2022) | *In vivo* | Dendrobium water extracts | - ↓ p-ERK, p-p38 (phosphorylation) | Direct | Rat (SCOP) | 200 mg/kg (oral) |
| MAPK Pathway Inhibition | (P. Liu et al., 2024a) | *In vivo* | Linarin | - ↓ p38, JNK (protein) | Direct | Mouse (BAC+chronic pain/tail clamping) | 12.5, 25, 50 mg/kg (oral) |
| MAPK Pathway Inhibition | (Zhao et al., 2019) | *In vivo* | Paeoniflorin | - ↓ p-JNK, p-p38 (phosphorylation) | Direct | Mouse (hyperosmotic saline) | 0.01%, 0.1%, 1% (topical) |
| MAPK Pathway Inhibition | (Yi Han et al., 2023) | *In vivo* | Berberine | - ↓ p-p38, p-ERK1/2 (phosphorylation) | Direct | Mouse (SCOP+DS)+B22 | 0.5, 2 mg/mL (topical) |
| MAPK Pathway Inhibition | (Li D. et al., 2015) | *In vivo* | Yangyin Runmu Pills | - ↓ p-p38 (phosphorylation) - ↓ p38 (protein) | Direct | SD rat (bilateral orchiectomy) | 9 g/100 mL (gavage) |
| MAPK Pathway Inhibition | (Li et al., 2019) | *In vivo* | Yangyin Runmu Pills | - ↓ p38 (protein) | Direct | New Zealand rabbit (SCOP) | 0.782, 0.869, 1.738 g/kg (gavage) |
| MAPK Pathway Inhibition | (Song et al., 2023) | *In vivo* | Runmu Ling Eye Drops | - ↓ p-JNK/JNK, p-p38/p38 (phosphorylation ratio) | Direct | New Zealand rabbit (BAC) | 0.51 g/mL (topical) |
| MAPK Pathway Inhibition | (Li, 2021) | *In vitro* | Kaempferol | - ↓ p38 (protein, mRNA) | Direct | HCECs (hyperosmolarity) | 80 µM |
| MAPK Pathway Inhibition | (Chen et al., 2010) | *In vitro* | Curcumin | - ↓ p-p38, p-JNK (phosphorylation) | Direct | HCECs (hyperosmolarity) | 5 µM |
| MAPK Pathway Inhibition | (Ling et al., 2022) | *In vitro* | Dendrobium water extracts | - ↓ p-ERK (phosphorylation) | Direct | HKs (hyperosmolarity) | 250, 500 µg/mL |
| MAPK Pathway Inhibition | (Zhao et al., 2019) | *In vitro* | Paeoniflorin | - ↓ p-p38, p-JNK, p-ERK (phosphorylation) | Direct | HCECs (hyperosmolarity) | 0.01%, 0.1%, 1% |
| MAPK Pathway Inhibition | (Chao et al., 2016) | *In vitro* | Lutein | - ↓ p-p38, p-JNK1/2 (phosphorylation) | Direct | HCECs (hyperosmolarity) | 1, 3, 10 µM |
| MAPK Pathway Inhibition | (Kim et al., 2018) | *In vitro* | KIOM-2015EW | - ↓ p-p38, p-ERK, p-JNK (phosphorylation) | Direct | HCECs (hyperosmolarity) | 0.05, 0.1, 0.2 mg/mL |

**Supplementary Table S1 (Continued).** Detailed Evidence by Signaling Pathway for TCM Interventions in DED.

| **Pathway^a^** | **Ref.** | **Study Type** | **TCM Intervention Tested** | **Key Pathway-Related Finding^b^** | **Evidence Quality^c^** | **Experimental Model** | **Key Concentration / Dose^d^** |
| --- | --- | --- | --- | --- | --- | --- | --- |
| MAPK Pathway Inhibition | (Li D. et al., 2022) | *In vitro* | Yangyin Runmu Pills | - ↓ p38 (protein) | Direct | Primary rabbit LG cells (TNF-α) | Medicated serum |
| MAPK Pathway Inhibition | (Zhao et al., 2022b) | *In vitro* | Qishen prescription | - ↓ p-ERK1, p-p38 (phosphorylation) | Direct | HCECs (hyperosmolarity) | 15% medicated serum |
| MAPK Pathway Inhibition | (Chen et al., 2024) | *In vitro* | Sihuang Qingling Liquid | - ↓ p-p38, p-JNK, p-ERK (phosphorylation) | Direct | RAW 264.7 (LPS) | 10, 20 µg/mL |
| MAPK Pathway Inhibition | (Du, 2018) | *In vitro* | Xiaosheng Granules | - ↓ P-JNK, P-ERK (phosphorylation) | Direct | RAW264.7 (hyperosmolarity/cytokines) | 100, 400 µg/mL |
| MAPK Pathway Inhibition | (P. Liu et al., 2024a) | *In vitro* | Linarin | - ↓ TNF-α, IL-1β (protein) | Indirect | HCECs (hyperosmolarity) | 15 µM |
| SIRT Activation | (Liang et al., 2023) | *In vivo* | Salidroside | - ↑ SIRT1 (protein) | Direct | Mouse (BAC) | 0.5, 2 mM (topical) |
| SIRT Activation | (Dong et al., 2024) | *In vitro* | Puerarin | - ↑ SIRT1 (protein) | Direct | HCE-2 cells (hyperosmolarity) | 10, 30, 50 µM |
| SIRT Activation | (X. Liu et al., 2025) | *In vitro* | Oroxylin A | - ↑ SIRT3 (protein, mRNA) | Direct | HCECs (hyperosmolarity) | 8, 10 µg/mL |
| SIRT Activation | (Liang et al., 2023) | *In vitro* | Salidroside | - ↑ SIRT1 (protein) | Direct | HCECs (hyperosmolarity) | 25, 50, 100 µM |
| Nrf2 Antioxidant Pathway Activation | (Z. Liu et al., 2025) | *In vivo* | Astaxanthin | - ↑ Nrf2 (protein, mRNA) - ↓ Keap1 (protein, mRNA) | Direct | Mouse (BAC) | 100 mg/kg (oral) |
| Nrf2 Antioxidant Pathway Activation | (Lin et al., 2022) | *In vitro* | PS-CG nanocomposite | - ↑ Nrf2 (protein, mRNA) - ↑ Keap1 (protein) | Direct | HCECs (hyperosmolarity) | 2.5, 5, 10, 20 µg/mL |
| Nrf2 Antioxidant Pathway Activation | (Yingjun Zhang et al., 2021) | *In vitro* | Esculetin | - ↑ nuclear Nrf2 (protein) - ↑ Nrf2 (mRNA) | Direct | HCECs (H₂O₂) | 20, 40, 80 µM |

**Supplementary Table S1 (Continued).** Detailed Evidence by Signaling Pathway for TCM Interventions in DED.

| **Pathway^a^** | **Ref.** | **Study Type** | **TCM Intervention Tested** | **Key Pathway-Related Finding^b^** | **Evidence Quality^c^** | **Experimental Model** | **Key Concentration / Dose^d^** |
| --- | --- | --- | --- | --- | --- | --- | --- |
| Nrf2 Antioxidant Pathway Activation | (Z. Liu et al., 2025) | *In vitro* | Astaxanthin | - ↑ Nrf2 (protein, mRNA) - ↓ Keap1 (protein, mRNA) | Direct | HCECs (hyperosmolarity) | 5, 10 µM |
| Nrf2 Antioxidant Pathway Activation | (Li, 2023) | *In vitro* | PS-GA-RGD | - ↑ p-Nrf2 (phosphorylation) | Direct | RAW 264.7 (LPS) | 10 µM |
| Nrf2 Antioxidant Pathway Activation | (Li et al., 2023) | *In vitro* | Gallic Acid | - ↑ Nrf2 nuclear translocation - ↑ p-Nrf2 (phosphorylation) | Direct | HCECs (hyperosmolarity); RAW 264.7 (LPS) | 100 µM |
| Nrf2 Antioxidant Pathway Activation | (Zhou et al., 2020) | *In vitro* | Genistein | - ↑ nuclear NRF2 (protein) | Direct | iHCECs/priHCECs (hyperosmolarity) | 50 µmol/L |
| Nrf2 Antioxidant Pathway Inhibition | (Huang and Peng, 2024) | *In vitro* | Acteoside | - ↓ Nrf2 (protein, mRNA) | Direct | HCECs (H₂O₂) | 160 µM |
| PI3K/AKT Pathway Activation | (Qin et al., 2019) | *In vivo* | Mimeng Hua Eye Drop | - ↑ PI3K, AKT (protein) | Direct | Rabbit (castration) | 1.0, 1.5, 3.0 mg/mL (topical) |
| PI3K/AKT Pathway Inhibition | (Jiang et al., 2023) | *In vivo* | Qinpi Eye Drop | - ↓ p-AKT (phosphorylation) | Direct | Rabbit (autoimmune DED) | Eye drops (topical) |
| PI3K/AKT Pathway Inhibition | (Yi Han et al., 2023) | *In vivo* | Berberine | - ↓ p-AKT (phosphorylation) | Direct | Mouse (SCOP+DS) | 0.5, 2 mg/mL (topical) |
| PI3K/AKT Pathway Inhibition | (Huang et al., 2024) | *In vitro* | Quercetin | - ↑ PTEN (protein) - ↓ p-PI3K, p-AKT (phosphorylation) | Direct | HCECs (hyperosmolarity) | 100 µM |
| AMPK Pathway Activation | (Liang et al., 2023) | *In vivo* | Salidroside | - ↑ p-AMPK (phosphorylation) | Direct | Mouse (BAC) | 0.5, 2 mM (topical) |
| AMPK Pathway Inhibition | (H. Chen et al., 2025) | *In vivo* | Chlorogenic Acid | - ↓ p-AMPK/AMPK (phosphorylation ratio) | Direct | Mouse (SOD1⁻/⁻) | 50 mg/kg (i.m.) |
| AMPK Pathway Activation | (Liang et al., 2023) | *In vitro* | Salidroside | - ↑ p-AMPK (phosphorylation) | Direct | HCECs (hyperosmolarity) | 25, 50, 100 µM |

**Supplementary Table S1 (Continued).** Detailed Evidence by Signaling Pathway for TCM Interventions in DED.

| **Pathway^a^** | **Ref.** | **Study Type** | **TCM Intervention Tested** | **Key Pathway-Related Finding^b^** | **Evidence Quality^c^** | **Experimental Model** | **Key Concentration / Dose^d^** |
| --- | --- | --- | --- | --- | --- | --- | --- |
| MUC1/EGFR Activation | (Chu et al., 2021) | *In vivo* | Astragaloside IV | - ↑ MUC1, EGFR (protein/mRNA) | Direct | Rabbit (BAC) | 5, 10 µM (topical) |
| MUC1/EGFR Activation | (Chu et al., 2021) | *In vitro* | Astragaloside IV | - ↑ MUC1, EGFR (protein/mRNA) | Direct | HCECs (BAC) | 5, 10 µM |
| p53/mTOR Modulation | (Li et al., 2025) | *In vivo* | Evodiamine | - ↑ nuclear p-p53 (phosphorylation) - ↓ p-mTOR (phosphorylation) | Direct | Mouse (BAC/atropine) | 500 µM, 1 mM (topical) |
| p53/mTOR Modulation | (Li et al., 2025) | *In vitro* | Evodiamine | - ↑ p-p53 (nuclear translocation/phosphorylation) - ↓ p-mTOR (phosphorylation) | Direct | HCECs (hyperosmolarity) | 0.1 µM |
| HMOX1/HIF-1 Inhibition | (J. Wang et al., 2024) | *In vivo* | Qingxuan Runmu Decoction | - ↓ HMOX1, HIF-1α (protein) | Direct | Wistar rat (LG excision) | equivalent dose (gavage) |
| HMOX1/HIF-1 Inhibition | (J. Wang et al., 2024) | *In vitro* | Qingxuan Runmu Decoction | - ↓ HMOX1, HIF-1α (protein) | Direct | HCE-2 cells (hyperosmolarity) | 2.5%, 5%, 10% medicated serum |
| VEGF Inhibition | (Lee et al., 2011) | *In vivo* | EGCG | - ↓ VEGF-A, VEGF-D (mRNA) | Direct | Mouse (controlled environment chamber) | 0.01%, 0.1% (topical) |
| VEGF Inhibition | (Hu, 2017) | *In vivo* | Qinpi Eye Drop | - ↓ VEGF-C, VEGFR-3 (protein, mRNA) | Direct | Mouse (controlled environment chamber) | Eye drops (topical) |
| PERK-eIF2α-ATF4-CHOP Pathway Inhibition | (Du, 2018) | *In vivo* | Xiaosheng Granules | Only clinical and histological outcomes measured; no in vivo pathway marker validation on ocular tissues | Phenotypic Only | C57BL/6 mouse (SCOP) | 3.6 mg/g (gavage) |
| PERK-eIF2α-ATF4-CHOP Pathway Inhibition | (Du, 2018) | *In vitro* | Xiaosheng Granules | - ↓ p-PERK, p-eIF2α (phosphorylation) - ↓ ATF4, CHOP (protein) | Direct | HCECs (hyperosmolarity/cytokines) | 100, 400 µg/mL |

**Supplementary Table S1 (Continued).** Detailed Evidence by Signaling Pathway for TCM Interventions in DED.

| **Pathway^a^** | **Ref.** | **Study Type** | **TCM Intervention Tested** | **Key Pathway-Related Finding^b^** | **Evidence Quality^c^** | **Experimental Model** | **Key Concentration / Dose^d^** |
| --- | --- | --- | --- | --- | --- | --- | --- |
| Bax/Caspase-9/Caspase-3 Inhibition | (Chen L. et al., 2023) | *In vivo* | Modified Siwei Dafa Powder | - ↓ Bax, Caspase-9 (protein) - ↑ Bcl-2 (protein) | Direct | SD rat (low-T and low-H chamber) | 5.89 g/kg (gavage) |
| PKA/CREB Activation | (Zhao and Wei, 2024) | *In vivo* | Polydatin | - ↑ p-PKA/PKA, p-CREB/CREB (phosphorylation ratio) | Direct | Rat (SCOP) | 0.05%, 0.5% (topical) |
| TRAF6/TAK1 Inhibition | (Zhao et al., 2024a) | *In vivo* | Qingxuan Runmu Decoction | - ↓ TRAF6 (protein) - ↓ p-TAK1/TAK1 (phosphorylation ratio) | Direct | SD rat (BAC) | 0.6 g/mL (gavage) |
| TLR4/MyD88 Inhibition | (Zhao et al., 2024b) | *In vivo* | Qingxuan Runmu Decoction | - ↓ TLR4, MyD88 (mRNA) | Direct | SD rat (BAC) | 0.6 g/mL (gavage) |
| IRAK1/TRAF6 Inhibition | (Liu T. et al., 2024) | *In vivo* | Runmu Xiaoyao Powder | - ↓ IRAK1, TRAF6 (protein, mRNA) | Direct | C57BL/6J mouse (BAC+pain) | 7.25, 14.5, 29 g/kg (gavage) |
| Caspase-8/Caspase-3 Inhibition | (Wu et al., 2022) | *In vitro* | Zhenzhu Mingmu Eye Drops | - ↓ cleaved Caspase-8, cleaved Caspase-3 (protein) | Direct | HCECs (TNF-α+IFN-γ) | 50×, 100×, 1000× dilution |
| JNK1/AQP5 Pathway Inhibition | (Zhao et al., 2022a) | *In vitro* | Qishen Prescription | - ↓ p-JNK1 (phosphorylation) - ↓ AQP5 (protein) | Direct | HCECs (hyperosmolarity) | 15% medicated serum |
| STAT1 Activation | (Wang et al., 2010) | *In vitro* | Buddleia Flavonoids | - ↑ p-STAT1 (phosphorylation) | Direct | LGECs (H₂O₂) | 8.95×10⁻² mol/L medicated plasma |
| Notch Activation | (Shetty et al., 2020) | *In vitro* | Resveratrol | - ↑ Notch2, Notch4, Hes1 (protein, mRNA) | Direct | HCE-T cells (hyperosmolarity) | 25 µM |
| FcγR-mediated phagocytosis Inhibition | (P. Liu et al., 2024b) | *In vivo* | Modified Danzhi Xiaoyao Powder | - ↓ phagocytic function of macrophages (Flow cytometry) - ↓ CDC42, ARPC2, ACTR3 (protein, mRNA) | Functional | C57 mouse (BAC+pain) | 6.24, 12.48, 24.96 g/kg (gavage) |

Footnotes for Supplementary Table S1:

a. Sorting: Pathways sorted by Overall Evidence Strength (Table 1). Within each pathway: Study Type (*In vivo* > *In vitro*) then Evidence Quality (Direct > Indirect > Functional > Phenotypic).

b. Key Pathway-Related Finding: ↑ indicates upregulation/activation; ↓ indicates downregulation/inhibition.

c. Evidence Quality: Categories defined in Table 1 footnote c.

d. Key Concentration / Dose: For *in vivo*, effective dose(s) and route shown. For *in vitro*, effective concentration(s) shown.

**Supplementary Table S2.** Botanical and Chemical Definition of Pure Metabolites and Key Botanical Drug Extracts.

| **Ref.** | **Active Metabolite/Extract** | **Type** | **Source Plant (Validated Scientific Name)** | **Pharmaceutical Name (Chinese)** | **Pharmaceutical Name (English)** | **Key Chemical Definition/ Standardization** |
| --- | --- | --- | --- | --- | --- | --- |
| (Huang et al., 2024) | Quercetin | M | *Apocynum venetum* L. [Apocynaceae; apocyni veneti folium] | Luo Bu Ma Ye | Dogbane Leaf | Pure metabolite (flavonoid, purity not specified) |
| (Jiang et al., 2023) | Qinpi eye drop | E | *Fraxinus chinensis subsp. rhynchophylla* (Hance) A.E.Murray [Oleaceae; fraxini cortex] | Qin Pi | Chinese Ash | Standardized ophthalmic formulation (hospital preparation, approval no. Z05170733) |
| (Qin et al., 2019) | Mimeng Hua eye drop | E | *Buddleja officinalis* Maxim. [Scrophulariaceae; buddlejae flos] | Mi Meng Hua | Pale Butterflybush Flower | Standardized ophthalmic formulation (60% ethanol extract, macroporous resin purified, HPLC-quantified for total flavonoids and phenylpropanoids) |
| (Li, 2021) | Kaempferol | M | *Buddleja officinalis* Maxim. [Scrophulariaceae; buddlejae flos] | Mi Meng Hua | Pale Butterflybush Flower | Pure metabolite (flavonol, purity not specified) |
| (Cao et al., 2024) | Polydatin | M | *Reynoutria japonica* Houtt. [Polygonaceae; polygoni cuspidati rhizoma et radix] | Hu Zhang | Giant Knotweed Rhizome | Pure metabolite (stilbenoid glucoside, >98% purity) |
| (Chen et al., 2010) | Curcumin | M | *Curcuma longa* L. [Zingiberaceae; curcumae longae rhizoma] | Jiang Huang | Turmeric | Pure metabolite (polyphenol, purity not specified) |
| (Li, 2020) | Berberine | M | *Coptis chinensis* Franch. [Ranunculaceae; coptidis rhizoma] | Huang Lian | Chinese Goldthread | Pure metabolite (Berberine hydrochloride, purity not specified) |
| (Wu, 2019) | Paeonol | M | *Paeonia × suffruticosa* Andrews [Paeoniaceae; Moutan cortex] | Mu Dan Pi | Tree Peony Bark | Pure metabolite (phenolic compound, purity not specified) |
| (Park et al., 2018) | PCE Aqueous Extract | E | *Reynoutria japonica* Houtt. [Polygonaceae; polygoni cuspidati rhizoma et radix] | Hu Zhang | Giant Knotweed Rhizome | Standardized aqueous extract (100°C water extraction, 10.5% yield; HPLC-quantified for caftaric acid, polydatin, rutin, quercitrin, resveratrol) |

**Supplementary Table S2 (Continued).** Botanical and Chemical Definition of Pure Metabolites and Key Botanical Drug Extracts.

| **Ref.** | **Active Metabolite/Extract** | **Type** | **Source Plant (Validated Scientific Name)** | **Pharmaceutical Name (Chinese)** | **Pharmaceutical Name (English)** | **Key Chemical Definition/ Standardization** |
| --- | --- | --- | --- | --- | --- | --- |
| (Yim et al., 2022) | KIOM-2015E | E | *Acer palmatum* Thunb. [Sapindaceae; Not recorded in pharmacopeia] | Ji Zhua Qi | Japanese Maple | Standardized botanical leaf extract (hot water extract: KIOM-2015EW; 25% ethanol extract: KIOM-2015EE; HPLC-quantified for 7 marker constituents, e.g., protocatechuic acid, orientin, isoorientin, and vitexin) |
| (X. Li et al., 2024) | Oridonin | M | *Isodon rubescens* (Hemsl.) H.Hara [Lamiaceae; rabdosiae rubescentis herba] | Dong Ling Cao | Blushred Rabdosia Leaf | Pure metabolite (ent-kaurane diterpenoid, purity not specified) |
| (Wu et al., 2024) | Huji Sheng eye drop | E | *Viscum coloratum* (Kom.) Nakai [Santalaceae; visci herba] | Hu Ji Sheng | Coloured Mistletoe Herb | Standardized ophthalmic formulation (solvent-extracted with ethanol/chloroform, final concentration determined) |
| (Lin et al., 2022) | Pterostilbene nanocomposite (PS-CG) | M | *Pterocarpus santalinus* L.f. [Fabaceae; Pterocarpi Lignum] | Zi Tan | Red Sandalwood | Synthetic nanocomposite (Pterostilbene conjugated to carboxyl-chitosan modified graphene via π-π stacking; purity not specified) |
| (Yingjun Zhang et al., 2021) | Esculetin | M | *Fraxinus chinensis subsp. rhynchophylla* (Hance) A.E.Murray [Oleaceae; fraxini cortex] | Qin Pi | Chinese Ash | Pure metabolite (6,7-dihydroxycoumarin; purity not specified) |
| (Huang and Peng, 2024) | Acteoside | M | *Cistanche deserticola* Ma [Orobanchaceae; cistanches herba] | Rou Cong Rong | Desertliving Cistanche | Pure metabolite (phenylethanoid glycoside, molecular formula C25H36O15, 624.59 Da; purity not specified) |
| (Z. Liu et al., 2025) | Astaxanthin | M | *Haematococcus pluvialis* Flot. [Haematococcaceae; Haematococci pulvialis pulvis] | Yu Sheng Hong Qiu Zao | Haematococcus Pluvialis | Pure metabolite (xanthophyll carotenoid, purity not specified) |
| (Dong et al., 2024) | Puerarin | M | *Pueraria montana var.* *thomsonii* (Benth.) M.R.Almeida [Fabaceae; puerariae thomsonii radix] | Ge Gen | Thomson Kudzuvine Root | Pure metabolite (isoflavone, purity not specified) |

**Supplementary Table S2 (Continued).** Botanical and Chemical Definition of Pure Metabolites and Key Botanical Drug Extracts.

| **Ref.** | **Active Metabolite/Extract** | **Type** | **Source Plant (Validated Scientific Name)** | **Pharmaceutical Name (Chinese)** | **Pharmaceutical Name (English)** | **Key Chemical Definition/ Standardization** |
| --- | --- | --- | --- | --- | --- | --- |
| (X. Liu et al., 2025) | Oroxylin A | M | *Scutellaria baicalensis* Georgi [Lamiaceae; scutellariae radix] | Huang Qin | Baical Skullcap Root | Pure metabolite (flavonoid, purity not specified) |
| (H. Chen et al., 2025) | Chlorogenic Acid | M | *Xanthium strumarium* L. [Asteraceae; xanthii fructus] | Cang Er Zi | Siberian Cocklebur Fruit | Pure metabolite (phenolic acid, 99.57% purity) |
| (Liang et al., 2023) | Salidroside | M | *Rhodiola rosea* L. [Crassulaceae; rhodiolae crenulatae radix et rhizoma] | Hong Jing Tian | Arctic Root | Pure metabolite (phenylethanoid glycoside, ≥98% purity) |
| (Lee et al., 2011) | EGCG | M | *Camellia sinensis* (L.) Kuntze [Theaceae; Theae Folium] | Lv Cha | Green Tea | Pure metabolite (catechin derivative, purity not specified) |
| (Hu, 2017) | Qinpi eye drop | E | *Fraxinus chinensis subsp. rhynchophylla* (Hance) A.E.Murray [Oleaceae; fraxini cortex] | Qin Pi | Chinese Ash | Standardized ophthalmic formulation (hospital preparation; self-made reagent by Longhua Hospital) |
| (Wang et al., 2010) | Buddleia flavonoids | E | *Buddleja officinalis* Maxim. [Scrophulariaceae; buddlejae flos] | Mi Meng Hua | Pale Butterflybush Flower | Extract from drug-containing plasma (total flavonoids >80%, primarily liarain/luteolin, ethanol extraction with water precipitation) |
| (Shetty et al., 2020) | Resveratrol | M | *Reynoutria japonica* Houtt. [Polygonaceae; polygoni cuspidati rhizoma et radix] | Hu Zhang | Giant Knotweed Rhizome | Pure metabolite (polyphenol, purity not specified) |
| (Zhao and Wei, 2024) | Polydatin | M | *Reynoutria japonica* Houtt. [Polygonaceae; polygoni cuspidati rhizoma et radix] | Hu Zhang | Giant Knotweed Rhizome | Pure metabolite (stilbenoid glucoside, ≥98% purity) |
| (Li et al., 2025) | Evodiamine | M | *Tetradium ruticarpum* (A.Juss.) T.G.Hartley [Rutaceae; euodiae fructus] | Wu Zhu Yu | Medicinal Evodia Fruit | Pure metabolite (quinoline alkaloid, >99% purity) |

**Supplementary Table S2 (Continued).** Botanical and Chemical Definition of Pure Metabolites and Key Botanical Drug Extracts.

| **Ref.** | **Active Metabolite/Extract** | **Type** | **Source Plant (Validated Scientific Name)** | **Pharmaceutical Name (Chinese)** | **Pharmaceutical Name (English)** | **Key Chemical Definition/ Standardization** |
| --- | --- | --- | --- | --- | --- | --- |
| (Chu et al., 2021) | Astragaloside IV | M | *Astragalus mongholicus* Bunge [Fabaceae; Astragali radix] | Huang Qi | Milkvetch Root | Pure metabolite (saponin, CAS 83207-58-3, referred to as a “high-purity drug”; specific purity percentage not specified) |
| (Ling et al., 2022) | Dendrobium Water Extracts (DOW/DLW) | E | *Dendrobium officinale* Kimura & Migo [Orchidaceae; dendrobii officinalis caulis]; *Dendrobium loddigesii* Rolfe [Orchidaceae; dendrobii caulis] | Tie Pi Shi Hu; Mei Hua Shi Hu | Noble Dendrobium; Loddiges’ Dendrobium | Standardized water extract (preparation method described, specific chemical standardization not detailed) |
| (P. Liu et al., 2024a) | Linarine | M | *Buddleja officinalis* Maxim. [Scrophulariaceae; buddlejae flos] | Mi Meng Hua | Pale Butterflybush Flower | Pure metabolite (flavonoid, purity not specified) |
| (Zhao et al., 2019) | Paeoniflorin | M | *Paeonia lactiflora* Pall. [Paeoniaceae; Paeoniae radix alba] | Bai Shao | White Peony Root | Pure metabolite (monoterpene glycoside, CAS 23180-57-6, 98% purity) |
| (Chao et al., 2016) | Lutein | M | *Calendula officinalis* L. [Asteraceae; calendulae flos] | Jin Zhan Hua | Calendula | Pure metabolite (xanthophyll carotenoid, purity not specified) |
| (Kim et al., 2018) | KIOM-2015EW | E | *Acer palmatum* Thunb. [Sapindaceae; Not recorded in pharmacopeia] | Ji Zhua Qi | Japanese Maple | Standardized hot-water extract (HPLC-DAD analysis identified orientin, isoorientin, and vitexin as marker compounds) |
| (Zhu et al., 2024) | Aurantio-obtusin | M | *Senna tora* (L.) Roxb. [Fabaceae; cassiae semen] | Jue Ming Zi | Cassia Seed | Pure metabolite (anthraquinone, >99% purity) |
| (Park et al., 2019) | Polydatin | M | *Reynoutria japonica* Houtt. [Polygonaceae; polygoni cuspidati rhizoma et radix] | Hu Zhang | Giant Knotweed Rhizome | Pure metabolite (stilbenoid glucoside, >98% purity) |
| (Yang et al., 2021) | SM934 | M | *Artemisia annua L.* [Asteraceae; artemisiae annuae herba] | Qing Hao | Sweet Wormwood Herb | Pure metabolite (β‑aminoarteether maleate, water‑soluble artemisinin derivative; purity not specified) |

**Supplementary Table S2 (Continued).** Botanical and Chemical Definition of Pure Metabolites and Key Botanical Drug Extracts.

| **Ref.** | **Active Metabolite/Extract** | **Type** | **Source Plant (Validated Scientific Name)** | **Pharmaceutical Name (Chinese)** | **Pharmaceutical Name (English)** | **Key Chemical Definition/ Standardization** |
| --- | --- | --- | --- | --- | --- | --- |
| (Li, 2023) | PS-GA-RGD Nano | M | *Pterocarpus santalinus* L.f. [Fabaceae; Pterocarpi Lignum] | Zi Tan | Red Sandalwood | Pure metabolite (stilbenoid-peptide conjugate, purity not specified) |
| (Li et al., 2023) | Gallic Acid | M | *Phyllanthus emblica* L. [Phyllanthaceae; phyllanthi fructus] | Yu Gan Zi | Emblic Leafflower Fruit | Pure metabolite (phenolic acid, ≥99% purity) |
| (Yi Han et al., 2023) | Berberine | M | *Coptis chinensis* Franch. [Ranunculaceae; coptidis rhizoma] | Huang Lian | Chinese Goldthread | Pure metabolite (isoquinoline alkaloid, berberine powder HPLC ≥98%) |
| (Zhou et al., 2020) | Genistein | M | *Spatholobus suberectus* Dunn [Fabaceae; spatholobi caulis] | Ji Xue Teng | Suberect Spatholobus Stem | Pure metabolite (isoflavone, purity not specified) |

**Supplementary Table S3.** Composition and Standardization Status of Included TCM Botanical Drug Formulas.

| **Ref.** | **Formula Name** | **Full Species Name with Authority and Family** | **Common English Name (Chinese Name)** | **Part(s) Used** | **Reported Quantity in Source (g or ratio)** | **Standardization Status^a^** | **Critical Note^b^** |
| --- | --- | --- | --- | --- | --- | --- | --- |
| (Li D. et al., 2015); (Li et al., 2019); (Li D. et al., 2022) | Yangyin Runmu Pills | *Rehmannia glutinosa* (Gaertn.) Libosch. ex DC. [Orobanchaceae; Rehmanniae radix] | Rehmannia Root (Di Huang) | Root tuber | 15 g | Laboratory-Prepared Product (Fixed Across Studies) | Fixed composition across studies; prepared as drug-containing serum *in vitro* and as water decoction/pills *in vivo*; no metabolite standardization, detailed extraction protocols, or batch-to-batch quality control reported. |
|  |  | *Angelica sinensis* (Oliv.) Diels [Apiaceae; Angelicae sinensis radix] | Chinese Angelica (Dang Gui) | Root | 10 g |  |  |
|  |  | *Lycium barbarum* L. [Solanaceae; lycii fructus] | Barbary Wolfberry Fruit (Gou Qi Zi) | Fruit | 15 g |  |  |
|  |  | *Adenophora stricta* Miq. [Campanulaceae; Adenophorae radix] | Fourleaf Ladybell Root (Sha Shen) | Root | 20 g |  |  |
|  |  | *Paeonia lactiflora* Pall. [Paeoniaceae; Paeoniae radix alba] | White Peony Root (Bai Shao) | Root | 10 g |  |  |
|  |  | *Dendrobium nobile* Lindl. [Orchidaceae; Dendrobii caulis] | Dendrobium (Shi Hu) | Stem | 15 g |  |  |
|  |  | *Polygonatum sibiricum* Redouté [Asparagaceae; Polygonati rhizome] | Solomonseal Rhizome (Huang Jing) | Rhizome | 20 g |  |  |

**Supplementary Table S3 (Continued).** Composition and Standardization Status of Included TCM Botanical Drug Formulas.

| **Ref.** | **Formula Name** | **Full Species Name with Authority and Family** | **Common English Name (Chinese Name)** | **Part(s) Used** | **Reported Quantity in Source (g or ratio)** | **Standardization Status^a^** | **Critical Note^b^** |
| --- | --- | --- | --- | --- | --- | --- | --- |
|  |  | *Paeonia × suffruticosa* Andrews [Paeoniaceae; Moutan cortex] | Tree Peony Bark (Mu Dan Pi) | Root bark | 10 g |  |  |
|  |  | *Chrysanthemum × morifolium* (Ramat.) Hemsl. [Asteraceae; Chrysanthemi flos] | Chrysanthemum (Ju Hua) | capitulum | 10 g |  |  |
|  |  | *Astragalus mongholicus* Bunge [Fabaceae; Astragali radix] | Milkvetch Root (Huang Qi) | Root | 20 g |  |  |
|  |  | *Glycyrrhiza uralensis* Fisch. ex DC. [Fabaceae; glycyrrhizae radix et rhizoma] | Liquorice Root (Gan Cao) | rhizome, root | 6 g |  |  |
| (Zhao et al., 2024a); (Zhao et al., 2024b); (J. Wang et al., 2024) | Qingxuan Runmu Decoction (QXRMY) | *Scrophularia ningpoensis* Hemsl. [Scrophulariaceae; scrophulariae radix] | Figwort Root (Xuan Shen) | Root | 10 g | Laboratory-Prepared Product (Fixed Across Studies) | Fixed composition across studies; prepared as drug-containing serum *in vitro* and as water decoction i*n vivo*; extraction process briefly described without detailed parameters; no metabolite standardization, batch-to-batch consistency, or quality control data reported. |
|  |  | *Ophiopogon japonicus* (Thunb.) Ker Gawl. [Asparagaceae; ophiopogonis radix] | Dwarf Lilyturf Tuber (Mai Dong) | root tuber | 10 g |  |  |

**Supplementary Table S3 (Continued).** Composition and Standardization Status of Included TCM Botanical Drug Formulas.

| **Ref.** | **Formula Name** | **Full Species Name with Authority and Family** | **Common English Name (Chinese Name)** | **Part(s) Used** | **Reported Quantity in Source (g or ratio)** | **Standardization Status^a^** | **Critical Note^b^** |
| --- | --- | --- | --- | --- | --- | --- | --- |
|  |  | *Rehmannia glutinosa* (Gaertn.) Libosch. ex DC. [Orobanchaceae; Rehmanniae radix] | Rehmannia Root (Di Huang) | Root tuber | 10 g |  |  |
|  |  | *Atractylodes macrocephala* Koidz. [Asteraceae; atractylodis macrocephalae rhizoma] | Largehead Atractylodes Rhizome (Bai Zhu) | Rhizome | 10 g |  |  |
|  |  | *Dendrobium nobile* Lindl. [Orchidaceae; Dendrobii caulis] | Dendrobium (Shi Hu) | Stem | 10 g |  |  |
|  |  | *Lonicera japonica* Thunb. [Caprifoliaceae; lonicerae japonicae flos] | Honeysuckle Flower (Jin Yin Hua) | branch, stem, flower | 10 g |  |  |
|  |  | *Forsythia suspensa* (Thunb.) Vahl [Oleaceae; forsythiae fructus] | Weeping Forsythia Capsule (Lian Qiao) | fruit | 10 g |  |  |
|  |  | *Saposhnikovia divaricata* (Turcz. ex Ledeb.) Schischk. [Apiaceae; saposhnikoviae radix] | Divaricate Saposhnikovia Root (Fang Feng) | Root | 10 g |  |  |

**Supplementary Table S3 (Continued).** Composition and Standardization Status of Included TCM Botanical Drug Formulas.

| **Ref.** | **Formula Name** | **Full Species Name with Authority and Family** | **Common English Name (Chinese Name)** | **Part(s) Used** | **Reported Quantity in Source (g or ratio)** | **Standardization Status^a^** | **Critical Note^b^** |
| --- | --- | --- | --- | --- | --- | --- | --- |
|  |  | *Platycodon grandiflorus* (Jacq.) A.DC. [Campanulaceae; platycodonis radix] | Platycodon Root (Jie Geng) | Root | 10 g |  |  |
|  |  | *Glycyrrhiza uralensis* Fisch. ex DC. [Fabaceae; glycyrrhizae radix et rhizoma] | Liquorice Root (Gan Cao) | rhizome, root | 10 g |  |  |
| (Zhao et al., 2022b); (Zhao et al., 2022a) | Qishen prescription | *Rehmannia glutinosa* (Gaertn.) Libosch. ex DC. [Orobanchaceae; rehmanniae radix] | Rehmannia Root (Shu Di Huang) | root tuber | 20 g | Laboratory-Prepared Product (Fixed Across Studies) | Fixed composition across two studies; prepared as drug-containing serum *in vitro*; no metabolite standardization, detailed extraction protocols, or batch-to-batch quality control reported. |
|  |  | *Dioscorea oppositifolia* L. [Dioscoreaceae; dioscoreae rhizoma] | Common Yam Rhizome (Shan Yao) | Rhizome | 9 g |  |  |
|  |  | *Cornus officinalis* Siebold & Zucc. [Cornaceae; corni fructus] | Asiatic Cornelian Cherry Fruit (Shan Zhu Yu) | Sarcocarp | 9 g |  |  |
|  |  | *Paeonia × suffruticosa* Andrews [Paeoniaceae; Moutan cortex] | Tree Peony Bark (Mu Dan Pi) | Root bark | 9 g |  |  |

**Supplementary Table S3 (Continued).** Composition and Standardization Status of Included TCM Botanical Drug Formulas.

| **Ref.** | **Formula Name** | **Full Species Name with Authority and Family** | **Common English Name (Chinese Name)** | **Part(s) Used** | **Reported Quantity in Source (g or ratio)** | **Standardization Status^a^** | **Critical Note^b^** |
| --- | --- | --- | --- | --- | --- | --- | --- |
|  |  | *Alisma plantago-aquatica subsp. orientale* (Sam.) Sam. [Alismataceae; alismatis rhizoma] | Oriental Waterplantain Rhizome (Ze Xie) | Tuber | 6 g |  |  |
|  |  | *Wolfiporia cocos* (F.A.Wolf) Ryvarden & Gilb. [Fomitopsidaceae; Poria] | Poria (Fu Ling) | Sclerotium | 6 g |  |  |
|  |  | *Lycium barbarum* L. [Solanaceae; lycii fructus] | Barbary Wolfberry Fruit (Gou Qi Zi) | Fruit | 6 g |  |  |
|  |  | *Chrysanthemum × morifolium* (Ramat.) Hemsl. [Asteraceae; Chrysanthemi flos] | Chrysanthemum (Ju Hua) | capitulum | 6 g |  |  |
|  |  | *Panax ginseng* C.A.Mey. [Araliaceae; ginseng radix et rhizoma] | Ginseng (Ren Shen) | Root | 3 g |  |  |
|  |  | *Ophiopogon japonicus* (Thunb.) Ker Gawl. [Asparagaceae; ophiopogonis radix] | Dwarf Lilyturf Tuber (Mai Dong) | root tuber | 9 g |  |  |

**Supplementary Table S3 (Continued).** Composition and Standardization Status of Included TCM Botanical Drug Formulas.

| **Ref.** | **Formula Name** | **Full Species Name with Authority and Family** | **Common English Name (Chinese Name)** | **Part(s) Used** | **Reported Quantity in Source (g or ratio)** | **Standardization Status^a^** | **Critical Note^b^** |
| --- | --- | --- | --- | --- | --- | --- | --- |
|  |  | *Schisandra chinensis* (Turcz.) Baill. [Schisandraceae; schisandrae chinensis fructus] | Chinese Magnoliavine Fruit (Wu Wei Zi) | Fruit | 6 g |  |  |
|  |  | *Tetrapanax papyrifer* (Hook.) K.Koch [Araliaceae; tetrapanacis medulla] | Ricepaperplant Pith (Tong Cao) | stem pith | 6 g |  |  |
|  |  | *Polygonatum sibiricum* Redouté [Asparagaceae; Polygonati rhizome] | Solomonseal Rhizome (Huang Jing) | Rhizome | 9 g |  |  |
|  |  | *Pueraria montana var.* *thomsonii* (Benth.) M.R.Almeida [Fabaceae; puerariae thomsonii radix] | Thomson Kudzuvine Root (Ge Gen) | root | 9 g |  |  |

**Supplementary Table S3 (Continued).** Composition and Standardization Status of Included TCM Botanical Drug Formulas.

| **Ref.** | **Formula Name** | **Full Species Name with Authority and Family** | **Common English Name (Chinese Name)** | **Part(s) Used** | **Reported Quantity in Source (g or ratio)** | **Standardization Status^a^** | **Critical Note^b^** |
| --- | --- | --- | --- | --- | --- | --- | --- |
| (Luo et al., 2024) | Runmu Ling granules (RMLG) | *Bidens pilosa* L. [Asteraceae; Bidentis herba] | Spanish Needles (Gui Zhen Cao) | Whole plant | weighed in a 3:1:1 ratio | Partially Characterized Product (Ratio Only) | Only botanical drug ratio (3:1:1) specified; prepared as granules *in vitro* and *in vivo* through a multi-step extraction process (decoction, ethanol precipitation, concentration, spray-drying, dry granulation); metabolite profiling by LC‑MS/MS identified 360 metabolites including flavonoids; however, no quantification of key marker compounds (e.g., chlorogenic acid, hyperoside) or batch-to-batch quality control was performed. |
|  |  | *Lycium barbarum* L. [Solanaceae; lycii fructus] | Barbary Wolfberry Fruit (Gou Qi Zi) | Fruit |  |  |  |
|  |  | *Chrysanthemum × morifolium* (Ramat.) Hemsl. [Asteraceae; Chrysanthemi flos] | Chrysanthemum (Ju Hua) | capitulum |  |  |  |
| (Song et al., 2023) | Runmu Ling Eye Drops | *Bidens pilosa* L. [Asteraceae; Bidentis herba] | Spanish Needles (Gui Zhen Cao) | Whole plant | 30 g | Partially Characterized Product (Single Study Defined) | Botanical drug quantities specified; prepared as water extract *in vivo*; extraction protocols (water volume, soaking, boiling time, filtration) provided, with a comparison between original and optimized processes; key flavonoid metabolites (chlorogenic acid, hyperoside, cynaroside) quantified by HPLC in both original and optimized formulas; however, the product is not fully standardized, and comprehensive quality control data are limited. |
|  |  | *Lycium barbarum* L. [Solanaceae; lycii fructus] | Barbary Wolfberry Fruit (Gou Qi Zi) | fruit | 15 g |  |  |
|  |  | *Chrysanthemum × morifolium* (Ramat.) Hemsl. [Asteraceae; Chrysanthemi flos] | Chrysanthemum (Ju Hua) | capitulum | 6 g |  |  |

**Supplementary Table S3 (Continued).** Composition and Standardization Status of Included TCM Botanical Drug Formulas.

| **Ref.** | **Formula Name** | **Full Species Name with Authority and Family** | **Common English Name (Chinese Name)** | **Part(s) Used** | **Reported Quantity in Source (g or ratio)** | **Standardization Status^a^** | **Critical Note^b^** |
| --- | --- | --- | --- | --- | --- | --- | --- |
| (Liu T. et al., 2024) | Runmu Xiaoyao Powder | *Bupleurum chinense* DC. [Apiaceae; bupleuri radix] | Chinese Thorowax Root (Chai Hu) | Root | 12 g | Laboratory-Prepared Product (Single Study Defined) | Botanical drug quantities specified; prepared as water decoction *in vivo*; extraction protocols (soaking, boiling, filtration, concentration) provided, but no metabolite standardization, detailed batch-to-batch quality control, or comprehensive chemical profiling reported. |
|  |  | *Angelica sinensis* (Oliv.) Diels [Apiaceae; Angelicae sinensis radix] | Chinese Angelica (Dang Gui) | Root | 10 g |  |  |
|  |  | *Paeonia lactiflora* Pall. [Paeoniaceae; Paeoniae radix alba] | White Peony Root (Bai Shao) | Root | 10 g |  |  |
|  |  | *Wolfiporia cocos* (F.A.Wolf) Ryvarden & Gilb. [Fomitopsidaceae; Poria] | Poria (Fu Ling) | Sclerotium | 10 g |  |  |
|  |  | *Atractylodes macrocephala* Koidz. [Asteraceae; atractylodis macrocephalae rhizoma] | Largehead Atractylodes Rhizome (Bai Zhu) | Rhizome | 10 g |  |  |
|  |  | *Lycium barbarum* L. [Solanaceae; lycii fructus] | Barbary Wolfberry Fruit (Gou Qi Zi) | fruit | 10 g |  |  |
|  |  | *Chrysanthemum × morifolium* (Ramat.) Hemsl. [Asteraceae; Chrysanthemi flos] | Chrysanthemum (Ju Hua) | capitulum | 10 g |  |  |

**Supplementary Table S3 (Continued).** Composition and Standardization Status of Included TCM Botanical Drug Formulas.

| **Ref.** | **Formula Name** | **Full Species Name with Authority and Family** | **Common English Name (Chinese Name)** | **Part(s) Used** | **Reported Quantity in Source (g or ratio)** | **Standardization Status^a^** | **Critical Note^b^** |
| --- | --- | --- | --- | --- | --- | --- | --- |
|  |  | *Buddleja officinalis* Maxim. [Scrophulariaceae; buddlejae flos] | Pale Butterflybush Flower (Mi Meng Hua) | Flower | 10 g |  |  |
|  |  | *Zingiber officinale* Roscoe [Zingiberaceae; zingiberis rhizoma recens] | Ginger (Sheng Jiang) | Rhizome | 6 g |  |  |
|  |  | *Paeonia × suffruticosa* Andrews [Paeoniaceae; Moutan cortex] | Tree Peony Bark (Mu Dan Pi) | Root bark | 10 g |  |  |
|  |  | *Gardenia jasminoides* J.Ellis [Rubiaceae; gardeniae fructus] | Cape Jasmine Fruit (Zhi Zi) | Fruit | 10 g |  |  |
|  |  | *Glycyrrhiza uralensis* Fisch. ex DC. [Fabaceae; glycyrrhizae radix et rhizoma] | Liquorice Root (Gan Cao) | rhizome, root | 6 g |  |  |

**Supplementary Table S3 (Continued).** Composition and Standardization Status of Included TCM Botanical Drug Formulas.

| **Ref.** | **Formula Name** | **Full Species Name with Authority and Family** | **Common English Name (Chinese Name)** | **Part(s) Used** | **Reported Quantity in Source (g or ratio)** | **Standardization Status^a^** | **Critical Note^b^** |
| --- | --- | --- | --- | --- | --- | --- | --- |
| (P. Liu et al., 2024b) | Modified Danzhi Xiaoyao Powder (MDXP) | *Paeonia × suffruticosa* Andrews [Paeoniaceae; Moutan cortex] | Tree Peony Bark (Mu Dan Pi) | Root bark | 10 g | Partially Characterized Product (Single Study Defined) | Botanical drug quantities specified; prepared as water extract *in vitro* and *in vivo*; analyzed by LC‑MS/MS for multiple metabolite classes (e.g., flavonoids, terpenoids), identifying key constituents, but not a fully standardized product; no detailed extraction protocols or batch-to-batch quality control data reported. |
|  |  | *Gardenia jasminoides* J.Ellis [Rubiaceae; gardeniae fructus] | Cape Jasmine Fruit (Zhi Zi) | Fruit | 10 g |  |  |
|  |  | *Angelica sinensis* (Oliv.) Diels [Apiaceae; Angelicae sinensis radix] | Chinese Angelica (Dang Gui) | Root | 10 g |  |  |
|  |  | *Paeonia lactiflora* Pall. [Paeoniaceae; Paeoniae radix alba] | White Peony Root (Bai Shao) | Root | 10 g |  |  |
|  |  | *Wolfiporia cocos* (F.A.Wolf) Ryvarden & Gilb. [Fomitopsidaceae; Poria] | Poria (Fu Ling) | Sclerotium | 10 g |  |  |
|  |  | *Atractylodes macrocephala* Koidz. [Asteraceae; atractylodis macrocephalae rhizoma] | Largehead Atractylodes Rhizome (Bai Zhu) | Rhizome | 10 g |  |  |
|  |  | *Bupleurum chinense* DC. [Apiaceae; bupleuri radix] | Chinese Thorowax Root (Chai Hu) | Root | 10 g |  |  |

**Supplementary Table S3 (Continued).** Composition and Standardization Status of Included TCM Botanical Drug Formulas.

| **Ref.** | **Formula Name** | **Full Species Name with Authority and Family** | **Common English Name (Chinese Name)** | **Part(s) Used** | **Reported Quantity in Source (g or ratio)** | **Standardization Status^a^** | **Critical Note^b^** |
| --- | --- | --- | --- | --- | --- | --- | --- |
|  |  | *Buddleja officinalis* Maxim. [Scrophulariaceae; buddlejae flos] | Pale Butterflybush Flower (Mi Meng Hua) | Flower | 10 g |  |  |
|  |  | *Chrysanthemum × morifolium* (Ramat.) Hemsl. [Asteraceae; Chrysanthemi flos] | Chrysanthemum (Ju Hua) | capitulum | 10 g |  |  |
|  |  | *Glycyrrhiza uralensis* Fisch. ex DC. [Fabaceae; glycyrrhizae radix et rhizoma] | Liquorice Root (Gan Cao) | rhizome, root | 10 g |  |  |
| (Chen et al., 2024) | Sihuang Qingling Liquid (SHQLY) | *Polygonatum sibiricum* Redouté [Asparagaceae; Polygonati rhizome] | Solomonseal Rhizome (Huang Jing) | Rhizome | 10 g | Laboratory-Prepared Product (Single Study Defined) | Botanical drug quantities specified; prepared as water extract *in vitro*; extraction protocols (soaking, double decoction with boiling and simmering, combination, concentration, freeze-drying) provided, but no metabolite standardization, batch-to-batch consistency, or comprehensive quality control data reported. |
|  |  | *Scutellaria baicalensis* Georgi [Lamiaceae; scutellariae radix] | Baical Skullcap Root (Huang Qin) | root | 10 g |  |  |
|  |  | *Coptis chinensis* Franch. [Ranunculaceae; coptidis rhizoma] | Chinese Goldthread (Huang Lian) | rhizome | 10 g |  |  |

**Supplementary Table S3 (Continued).** Composition and Standardization Status of Included TCM Botanical Drug Formulas.

| **Ref.** | **Formula Name** | **Full Species Name with Authority and Family** | **Common English Name (Chinese Name)** | **Part(s) Used** | **Reported Quantity in Source (g or ratio)** | **Standardization Status^a^** | **Critical Note^b^** |
| --- | --- | --- | --- | --- | --- | --- | --- |
|  |  | *Chrysanthemum × morifolium* (Ramat.) Hemsl. [Asteraceae; Chrysanthemi flos] | Chrysanthemum (Ju Hua) | Capitulum | 10 g |  |  |
|  |  | *Mentha canadensis* L. [Lamiaceae; menthae haplocalycis herba] | Peppermint (Bo He) | aerial parts | 10 g |  |  |
| (Chen L. et al., 2023) | Modified Siwei Dafa Powder | *Ephedra sinica* Stapf [Ephedraceae; ephedrae herba] | Ephedra (Ma Huang) | Stem | 5 g | Laboratory-Prepared Product (Single Study Defined) | Botanical drug quantities specified; prepared as water decoction (concentrated) *in vivo*; extraction process briefly described without detailed parameters; no metabolite standardization, batch-to-batch consistency, or quality control data reported. |
|  |  | *Conioselinum anthriscoides* (H.Boissieu) Pimenov & Kljuykov [Apiaceae; ligustici rhizoma et radix] | Chinese Lovage Root (Gao Ben) | rhizome, root | 5 g |  |  |
|  |  | *Vitex trifolia* L. [Lamiaceae; viticis fructus] | Shrub Chastetree Fruit (Man Jing Zi) | Fruit | 5 g |  |  |
|  |  | Asarum heterotropoides F.Schmidt [Aristolochiaceae; asari radix et rhizoma] | Manchurian Wildginger Root (Xi Xin) | rhizome, root | 3 g |  |  |

**Supplementary Table S3 (Continued).** Composition and Standardization Status of Included TCM Botanical Drug Formulas.

| **Ref.** | **Formula Name** | **Full Species Name with Authority and Family** | **Common English Name (Chinese Name)** | **Part(s) Used** | **Reported Quantity in Source (g or ratio)** | **Standardization Status^a^** | **Critical Note^b^** |
| --- | --- | --- | --- | --- | --- | --- | --- |
|  |  | *Zingiber officinale* Roscoe [Zingiberaceae; zingiberis rhizoma recens] | Ginger (Sheng Jiang) | Rhizome | 5 g |  |  |
|  |  | Scrophularia ningpoensis Hemsl. [Scrophulariaceae; scrophulariae radix] | Figwort Root (Xuan Shen) | Root | 15 g |  |  |
|  |  | Ophiopogon japonicus (Thunb.) Ker Gawl. [Asparagaceae; ophiopogonis radix] | Dwarf Lilyturf Tuber (Mai Dong) | root tuber | 10 g |  |  |
|  |  | *Rehmannia glutinosa* (Gaertn.) Libosch. ex DC. [Orobanchaceae; Rehmanniae radix] | Rehmannia Root (Di Huang) | Root tuber | 10 g |  |  |
| (Yang J. et al., 2023) | Huashi Runjing Decoction | *Prunus armeniaca* L. [Rosaceae; armeniacae semen amarum] | Bitter Apricot Seed (Ku Xing Ren) | Seed | 10 g | Laboratory-Prepared Product (Single Study Defined) | Botanical drug quantities specified; prepared as formula granules (reconstituted in hot water) *in vivo*; no metabolite standardization, detailed extraction protocols, or batch-to-batch quality control reported. |
|  |  | *Coix lacryma-jobi var. ma-yuen* (Rom.Caill.) Stapf [Poaceae; coicis semen] | Coix Seed (Yi Yi Ren) | Fruit | 30 g |  |  |

**Supplementary Table S3 (Continued).** Composition and Standardization Status of Included TCM Botanical Drug Formulas.

| **Ref.** | **Formula Name** | **Full Species Name with Authority and Family** | **Common English Name (Chinese Name)** | **Part(s) Used** | **Reported Quantity in Source (g or ratio)** | **Standardization Status^a^** | **Critical Note^b^** |
| --- | --- | --- | --- | --- | --- | --- | --- |
|  |  | *Amomum kravanh* Pierre ex Gagnep. [Zingiberaceae; Amomi fructus rotundus] | Round Cardamon Fruit (Dou Kou) | Fruit | 10 g |  |  |
|  |  | *Atractylodes macrocephala* Koidz. [Asteraceae; atractylodis macrocephalae rhizoma] | Largehead Atractylodes Rhizome (Bai Zhu) | Rhizome | 15 g |  |  |
|  |  | *Wolfiporia cocos* (F.A.Wolf) Ryvarden & Gilb. [Fomitopsidaceae; Poria] | Poria (Fu Ling) | Sclerotium | 15 g |  |  |
|  |  | *Bupleurum chinense* DC. [Apiaceae; bupleuri radix] | Chinese Thorowax Root (Chai Hu) | Root | 10 g |  |  |
|  |  | *Ligustrum lucidum* W.T.Aiton [Oleaceae; ligustri lucidi fructus] | Glossy Privet Fruit (Nv Zhen Zi) | fruit | 15 g |  |  |
|  |  | *Paeonia lactiflora* Pall. [Paeoniaceae; Paeoniae radix alba] | White Peony Root (Bai Shao) | Root | 15 g |  |  |
|  |  | *Citrus × aurantium* L. [Rutaceae; aurantii fructus immaturus] | Immature Orange Fruit (Zhi Shi) | Fruit | 10 g |  |  |

**Supplementary Table S3 (Continued).** Composition and Standardization Status of Included TCM Botanical Drug Formulas.

| **Ref.** | **Formula Name** | **Full Species Name with Authority and Family** | **Common English Name (Chinese Name)** | **Part(s) Used** | **Reported Quantity in Source (g or ratio)** | **Standardization Status^a^** | **Critical Note^b^** |
| --- | --- | --- | --- | --- | --- | --- | --- |
|  |  | *Carthamus tinctorius* L. [Asteraceae; carthami flos] | Safflower (Hong Hua) | Flower | 5 g |  |  |
|  |  | *Glycyrrhiza uralensis* Fisch. ex DC. [Fabaceae; glycyrrhizae radix et rhizoma] | Liquorice Root (Gan Cao) | rhizome, root | 5 g |  |  |
| (Wu et al., 2022) | Zhenzhu Mingmu Eye Drops (ZMED) | *Pinctada martensii* (Dunker) [Pteriidae; Margarita] | Pearl (Zhen Zhu) | Pearl | N/S^17^ | Commercial/Hospital Standardized Product (Contains Non‑botanical Material) | Contains non‑botanical components (pearl, borneol), quantities not specified; commercially available colloidal solution (Suzhou Industrial Park Tianlong Pharmaceutical Co., batch no. 20161006); used at specified dilutions *in vitro*; but no detailed manufacturing process, metabolite standardization, or batch-to-batch quality control reported. |
|  |  | *Camphora officinarum* Boerh. ex Fabr. [Lauraceae; borneolum] | Natural Borneol (Bing Pian) | branch, leaf | N/S |  |  |
| (Du, 2024) | Xiaosheng Granules | *Codonopsis pilosula* (Franch.) Nannf. [Campanulaceae; codonopsis radix] | Tangshen (Dang Shen) | Root | N/S | Laboratory-Prepared Product (Ingredients Listed Only) | Botanical drug ingredients listed without quantities; prepared in the laboratory according to a detailed multi-step extraction and purification protocol (volatile oil extraction, water decoction, ethanol precipitation, drying); no metabolite standardization or quality control reported. |
|  |  | *Angelica sinensis* (Oliv.) Diels [Apiaceae; Angelicae sinensis radix] | Chinese Angelica (Dang Gui) | Root | N/S |  |  |

**Supplementary Table S3 (Continued).** Composition and Standardization Status of Included TCM Botanical Drug Formulas.

| **Ref.** | **Formula Name** | **Full Species Name with Authority and Family** | **Common English Name (Chinese Name)** | **Part(s) Used** | **Reported Quantity in Source (g or ratio)** | **Standardization Status^a^** | **Critical Note^b^** |
| --- | --- | --- | --- | --- | --- | --- | --- |
|  |  | *Rehmannia glutinosa* (Gaertn.) Libosch. ex DC. [Orobanchaceae; Rehmanniae radix] | Rehmannia Root (Di Huang) | Root tuber | N/S |  |  |
|  |  | *Paeonia lactiflora* Pall. [Paeoniaceae; Paeoniae radix alba] | White Peony Root (Bai Shao) | Root | N/S |  |  |
|  |  | *Ophiopogon japonicus* (Thunb.) Ker Gawl. [Asparagaceae; ophiopogonis radix] | Dwarf Lilyturf Tuber (Mai Dong) | root tuber | N/S |  |  |
|  |  | *Schisandra chinensis* (Turcz.) Baill. [Schisandraceae; schisandrae chinensis fructus] | Chinese Magnoliavine Fruit (Wu Wei Zi) | Fruit | N/S |  |  |
|  |  | *Bupleurum chinense* DC. [Apiaceae; bupleuri radix] | Chinese Thorowax Root (Chai Hu) | Root | N/S |  |  |
|  |  | *Mentha canadensis* L. [Lamiaceae; menthae haplocalycis herba] | Peppermint (Bo He) | aerial parts | N/S |  |  |

**Supplementary Table S3 (Continued).** Composition and Standardization Status of Included TCM Botanical Drug Formulas.

| **Ref.** | **Formula Name** | **Full Species Name with Authority and Family** | **Common English Name (Chinese Name)** | **Part(s) Used** | **Reported Quantity in Source (g or ratio)** | **Standardization Status^a^** | **Critical Note^b^** |
| --- | --- | --- | --- | --- | --- | --- | --- |
| (Shi X. et al., 2023) | Erzhi Pills | *Ligustrum lucidum* W.T.Aiton [Oleaceae; ligustri lucidi fructus] | Glossy Privet Fruit (Nv Zhen Zi) | Fruit | N/S | Commercial/Hospital Standardized Product (Ingredients Listed Only) | Botanical drug ingredients listed without quantities; commercially available pills with a National Drug Approval Number (Z36020854, Jiangxi Renfeng Pharmaceutical Co., Ltd.), produced under GMP standards with batch-to-batch consistency confirmed by identical fingerprint chromatograms; however, no detailed extraction protocols, metabolite standardization, or comprehensive quality control data are reported. |
|  |  | *Eclipta prostrata* (L.) L. [Asteraceae; ecliptae herba] | Yerbadetajo Herb (Mo Han Lian) | aerial parts | N/S |  |  |
| (Dong, 2024) | Zhenshi Guben Liquid | *Rehmannia glutinosa* (Gaertn.) Libosch. ex DC. [Orobanchaceae; Rehmanniae radix] | Rehmannia Root (Di Huang) | Root tuber | N/S | Laboratory-Prepared Product (Ingredients Listed Only) | Botanical drug ingredients listed without quantities; prepared as a hospital-prepared concentrate (lot 230517) *in vivo*; no metabolite standardization, detailed extraction protocols, or quality control, or batch-to-batch consistency data reported. |
|  |  | *Rehmannia glutinosa* (Gaertn.) Libosch. ex DC. [Orobanchaceae; rehmanniae radix] | Rehmannia Root (Shu Di Huang) | root tuber | N/S |  |  |
|  |  | *Polygonatum odoratum* (Mill.) Druce [Asparagaceae; polygonati odorati rhizoma] | Fragrant Solomonseal Rhizome (Yu Zhu) | Rhizome | N/S |  |  |
|  |  | *Paeonia × suffruticosa* Andrews [Paeoniaceae; Moutan cortex] | Tree Peony Bark (Mu Dan Pi) | Root bark | N/S |  |  |

**Supplementary Table S3 (Continued).** Composition and Standardization Status of Included TCM Botanical Drug Formulas.

| **Ref.** | **Formula Name** | **Full Species Name with Authority and Family** | **Common English Name (Chinese Name)** | **Part(s) Used** | **Reported Quantity in Source (g or ratio)** | **Standardization Status^a^** | **Critical Note^b^** |
| --- | --- | --- | --- | --- | --- | --- | --- |
|  |  | *Angelica sinensis* (Oliv.) Diels [Apiaceae; Angelicae sinensis radix] | Chinese Angelica (Dang Gui) | Root | N/S |  |  |
|  |  | *Paeonia lactiflora* Pall. [Paeoniaceae; Paeoniae radix alba] | White Peony Root (Bai Shao) | Root | N/S |  |  |
|  |  | *Pueraria montana var.* *thomsonii* (Benth.) M.R.Almeida [Fabaceae; puerariae thomsonii radix] | Thomson Kudzuvine Root (Ge Gen) | Root | N/S |  |  |
|  |  | *Astragalus mongholicus* Bunge [Fabaceae; Astragali radix] | Milkvetch Root (Huang Qi) | Root | N/S |  |  |
|  |  | *Atractylodes macrocephala* Koidz. [Asteraceae; atractylodis macrocephalae rhizoma] | Largehead Atractylodes Rhizome (Bai Zhu) | Rhizome | N/S |  |  |
|  |  | *Wolfiporia cocos* (F.A.Wolf) Ryvarden & Gilb. [Fomitopsidaceae; Poria] | Poria (Fu Ling) | Sclerotium | N/S |  |  |

**Supplementary Table S3 (Continued).** Composition and Standardization Status of Included TCM Botanical Drug Formulas.

| **Ref.** | **Formula Name** | **Full Species Name with Authority and Family** | **Common English Name (Chinese Name)** | **Part(s) Used** | **Reported Quantity in Source (g or ratio)** | **Standardization Status^a^** | **Critical Note^b^** |
| --- | --- | --- | --- | --- | --- | --- | --- |
|  |  | *Rubus chingii* Hu [Rosaceae; rubi fructus] | Palmleaf Raspberry Fruit (Fu Pen Zi) | Fruit | N/S |  |  |
|  |  | *Senna tora* (L.) Roxb. [Fabaceae; cassiae semen] | Cassia Seed (Jue Ming Zi) | Seed | N/S |  |  |
|  |  | *Conioselinum anthriscoides 'Chuanxiong'* [Apiaceae; chuanxiong rhizoma] | Szechwan Lovage Rhizome (Chuan Xiong) | Rhizome | N/S |  |  |
|  |  | *Typha angustifolia* L. [Typhaceae; typhae pollen] | Cattail Pollen (Pu Huang) | Pollen | N/S |  |  |
|  |  | *Glycyrrhiza uralensis* Fisch. ex DC. [Fabaceae; glycyrrhizae radix et rhizoma] | Liquorice Root (Gan Cao) | rhizome, root | N/S |  |  |
|  |  | *Paeonia veitchii* Lynch [Paeoniaceae; paeoniae radix rubra] | Red Peony Root (Chi Shao) | Root | N/S |  |  |

**Supplementary Table S3 (Continued).** Composition and Standardization Status of Included TCM Botanical Drug Formulas.

| **Ref.** | **Formula Name** | **Full Species Name with Authority and Family** | **Common English Name (Chinese Name)** | **Part(s) Used** | **Reported Quantity in Source (g or ratio)** | **Standardization Status^a^** | **Critical Note^b^** |
| --- | --- | --- | --- | --- | --- | --- | --- |
|  |  | *Scleromitrion diffusum* (Willd.) R.J.Wang [Rubiaceae; Not recorded in pharmacopeia] | Spreading Hedyotis Herb (Bai Hua She She Cao) | Whole plant | N/S |  |  |

Footnotes for Supplementary Table S3:

a. Standardization Status: **Commercial/Hospital Standardized Product** = Marketed or hospital-prepared product with approval number (or GMP batch number) and quality control data (e.g., HPLC fingerprinting, assay of marker compounds). **Partially Characterized Product** = Targeted chemical analysis (e.g., HPLC quantification of key markers, LC‑MS/MS metabolite profiling) performed but not fully standardized preparation. **Laboratory-Prepared Product** = In-house preparation without quality control and chemical characterization. Subclass indicates composition clarity: (Fixed Across Studies), (Single Study Defined), (Ratio Only), (Ingredients Listed Only), or (Contains Non‑botanical Material).

b. Critical Note: Summarizes reproducibility, chemical definition, and quality control limitations for interpreting results.

**Supplementary Table S4.** Detailed Pharmacological and Experimental Parameters.

| **Ref.** | **Metabolite/Formula** | **Full Dose Range Tested** | **Minimal Effective Concentration/Dose** | **Controls** | **Toxic Side Effects** |
| --- | --- | --- | --- | --- | --- |
| (Huang et al., 2024) | Quercetin | ***In vitro* (mechanism):** 0, 5, 10, 20, 40, 80, 100, 150 µM (12 h)  ***In vitro* (cytotoxicity):** 0, 100, 200, 400, 800 µM (12 h) | ***In vitro*:** 100 µM | ***In vitro*:**  hyperosmotic medium (negative), isomolar medium (negative), hyperosmotic medium +LY294002 (PI3K inhibitor, positive) | ***In vitro*:** no significant cytotoxicity was observed at concentrations up to 100 µM |
| (Jiang et al., 2023) | Qinpi eye drop | ***In vivo*:** Eye drops (topical, 4×/d, 6 w) | ***In vivo*:** N/A | ***In vivo*:** healthy rabbits + PBS (negative), ADE rabbits + PBS (negative) | ***In vivo*:** not mentioned |
| (Qin et al., 2019) | Mimeng Hua eye drop | ***In vivo*:** 1.0, 1.5, 3.0 mg/mL (topical, 3×/d, 4 w) | ***In vivo*:** 1.5 mg/ml (topical) | ***In vivo*:** healthy rabbits (negative), castrated rabbits, no treatment (negative), castrated + testosterone injection (positive) | ***In vivo*:** not mentioned |
| (Li, 2021) | kaempferol | ***In vitro* (mechanism):** 80 µM (48 h)  ***In vitro* (cytotoxicity/viability):** 0, 20, 40, 80, 160, 320, 640, 1280 µM (24 h, 48 h) | ***In vitro*:** 80 µM | ***In vitro*:** isomolar medium (negative), 450 mOsm medium (negative), hyperosmotic medium + DMSO (negative) | ***In vitro*:** no significant cytotoxicity at concentrations up to 80 µM; toxicity observed at higher concentrations (≥160 µM) |
| (Cao et al., 2024) | polydatin (PD) | ***In vivo*:** 0.05%, 0.5% (topical, 3×/d, 28 d) | ***In vivo*:** 0.05% (topical) | ***In vivo*:** normal rats with saline (negative), DED rats with saline (negative), 0.5% polydatin + U0126 (MEK inhibitor, inhibitor control group) | ***In vivo*:** not mentioned |
| (Chen et al., 2010) | Curcumin | ***In vitro* (mechanism):** 5 µM (30 min pretreatment then hyperosmotic exposure)  ***In vitro* (cytotoxicity):** 1, 3, 5, 10, 30, 50 µM (24 h) | ***In vitro*:** 5 µM | ***In vitro*:** isomolar medium (negative), 450 mOsm medium (negative), hyperosmotic medium + SB203580 (p38 inhibitor, positive) | ***In vitro*:** no significant cytotoxicity at concentrations up to 30 µM; toxicity observed at 50 µM |
| (Li, 2020) | berberine (BBR) | ***In vivo*:** 0.5, 1, 2 mg/ml (topical, 4×/d, 7 d) | ***In vivo*:** 2 mg/mL (topical) | ***In vivo*:** normal mice + saline (negative), DS model mice + saline (negative), DS model mice + vehicle (saline, negative) | ***In vivo*:** not mentioned |
| (Wu, 2019) | paeonol | ***In vivo*:** 1%, 5%, 10% (topical, 4×/d, 5 d) | ***In vivo*:** 5% (topical) | ***In vivo*:** normal mice (negative), DED model mice (negative), DED model mice + PBS vehicle (negative). | ***In vivo*:** not mentioned |

**Supplementary Table S4 (Continued).** Detailed Pharmacological and Experimental Parameters.

| **Ref.** | **Metabolite/Formula** | **Full Dose Range Tested** | **Minimal Effective Concentration/Dose** | **Controls** | **Toxic Side Effects** |
| --- | --- | --- | --- | --- | --- |
| (Park et al., 2018) | Polygonum cuspidatum (PCE) Aqueous Extract | ***In vivo*:** 10, 100, 250 mg/kg (oral, 5 d)  ***In vitro* (cytotoxicity/mechanism):** 1, 10, 100 µg/mL (1 h or 24 h) | ***In vivo*:** 100 mg/kg (oral)  ***In vitro*:** 10 µg/mL | ***In vivo*:** normal rats (negative), vehicle-treated dry eye rats (negative)  ***In vitro*:** untreated cells (negative), hyperosmolar-treated cells (negative) | ***In vivo*:** not mentioned  ***In vitro*:** no significant cytotoxicity at concentrations up to 100 μg/mL; toxicity observed at 250 μg/mL |
| (Yim et al., 2022) | KIOM-2015E (KIOM-2015EW and KIOM-2015EE) | ***In vivo*:** 0.5, 1 mg/mL (topical, 3×/d, 14 d)  ***In vivo*:** 100 mg/kg (oral, 1×/d, 14 d) | ***In vivo*:** 0.5 mg/mL (topical)  ***In vivo*:** 100 mg/kg (oral) | ***In vivo*:** normal mice (negative), BAC-induced DED model (negative), DED model + PBS vehicle (negative), DED model + 0.5 mg/mL Cyclosporine A (standard immunosuppressant, positive), DED model + 1 mg/mL Fluorometholone (standard corticosteroid, positive). | ***In vivo*:** not explicitly reported; no adverse effects observed in the study at tested doses |
| (X. Li et al., 2024) | oridonin | ***In vivo*:** 0.01%, 0.1%, 1% (topical, 2×/d, 7 d)  ***In vitro* (mechanism):** 2, 4, 7 µM (pretreatment 4 h, then culture 24 h)  ***In vitro* (cytotoxicity):** 0, 2, 4, 7, 10, 20 µM (24 h) | ***In vivo*:** 0.01% (topical)  ***In vitro*:** 2 μM | ***In vivo*:** untreated normal mice (negative), BAC-induced DED model mice (negative), DED model mice + DMSO (negative)  ***In vitro*:** isomolar medium (negative), 500 mOsm medium (negative), hyperosmotic medium + DMSO (negative) | ***In vivo*:** higher concentrations (0.1%, 1%) showed reduced pharmacological effects, potentially due to drug toxicity or DMSO side effects  ***In vitro*:** Concentrations ≥ 10 μM significantly reduced HCE-T cell viability (IC₅₀ ≈ 12.44 μM) |
| (Wu et al., 2024) | Huji Sheng eye drop | ***In vivo*:** 0.005% (topical, 2×/d, 14 d) | ***In vivo*:** 0.005% (topical) | ***In vivo*:** healthy mice + normal saline (negative), DED model mice + normal saline (negative), DED model mice + VX-765 (NLRP3/Caspase-1 pathway inhibitor, inhibitor control group) | ***In vivo*:** not mentioned |
| (Lin et al., 2022) | PS-CG nanocomposite | ***In vivo*:** 4 μg/mL (topical, 2×/d, 14 d)  ***In vitro* (cytotoxicity/mechanism):** 2.5, 5, 10, 20 μg/mL (co-cultured over 48 h) | ***In vivo*:** 4 μg/mL  ***In vitro*:** 2.5 μg/mL | ***In vivo*:** healthy mice + normal saline (negative), DED model mice (negative), DED model mice + PS treatment (negative)  ***In vitro*:** isomolar medium (negative), 500 mOsm medium (negative), hyperosmotic medium + PS treatment (negative) | ***In vivo*:** no organ damage (HE staining showed no visible changes)  ***In vitro*:** no significant cytotoxicity at concentrations up to 20 μg/ml |

**Supplementary Table S4 (Continued).** Detailed Pharmacological and Experimental Parameters.

| **Ref.** | **Metabolite/Formula** | **Full Dose Range Tested** | **Minimal Effective Concentration/Dose** | **Controls** | **Toxic Side Effects** |
| --- | --- | --- | --- | --- | --- |
| (Yingjun Zhang et al., 2021) | Esculetin | ***In vivo*:** 0.002% (topical, 2×/d, 5 d)  ***In vitro* (mechanism):** 20, 40, 80 µM (2 h pretreatment + 24 h H₂O₂)  ***In vitro* (cytotoxicity/viability):** 0.01, 0.1, 1, 10, 20, 40, 80, 100 µM (24 h) | ***In vivo*:** 0.002% (topical)  ***In vitro* (mechanism):** 20 µM  ***In vitro* (cell viability):** 10 µM | ***In vivo*:** healthy mice (negative), DED model mice + vehicle (5% Tween-80 + 0.1% benzalkonium bromide in saline, negative), DED model mice + Cyclosporine A (positive)  ***In vitro*:** untreated normal HCE cells (negative), H₂O₂-only treated cells (negative), H₂O₂ + Trolox treatment (positive) | ***In vivo*:** not mentioned  ***In vitro*:** no significant cytotoxicity at concentrations up to 100 μM |
| (Huang and Peng, 2024) | Acteoside | ***In vitro* (mechanism):** 160 µM (24 h)  ***In vitro* (cytotoxicity/viability):** 5, 10, 20, 40, 80, 160, 320, 640 µM (24 h) | ***In vitro*:** 160 μM | ***In vitro*:** intact cells (negative), H₂O₂-only treated cells (negative), DMSO solvent control (negative) | ***In vitro*:** no significant cytotoxicity at 160 μM, higher concentrations not tested in detail |
| (Z. Liu et al., 2025) | Astaxanthin | ***In vivo*:** 100 mg/kg (oral, 1×/d, 7 d)  ***In vitro* (mechanism/cell viability):** 1.25, 2.5, 5, 10, 20 μM (24 h) | ***In vivo*:** 100 mg/kg (oral)  ***In vitro*:** 5 μM | ***In vivo*:** PBS-treated healthy mice (negative), BAC-induced DED model mice (negative)  ***In vitro*:** isotonic medium (negative), hyperosmotic medium (negative) | ***In vivo*:** no mentioned organ toxicity at 100 mg/kg  ***In vitro*:** no significant cytotoxicity at concentrations up to 10 μM |
| (Dong et al., 2024) | Puerarin | ***In vitro* (mechanism):** 10, 30, 50 µM (1 h pretreatment, then 24 h hyperosmotic exposure) | ***In vitro*:** 10 μM | ***In vitro*:** isotonic medium (negative), hyperosmotic stress (70 mM NaCl, negative), hyperosmotic stress + Puerarin + siRNA-NC (negative for silencing), hyperosmotic stress + Puerarin + siRNA-SIRT1 (mechanistic control) | ***In vitro*:** no significant cytotoxicity at 10–50 μM in HCE-2 cells |
| (X. Liu et al., 2025) | Oroxylin A (OA) | ***In vitro* (mechanism):** 4, 6, 8, 10 μg/mL (24 h co-treatment with hyperosmotic medium)  ***In vitro* (cytotoxicity/viability):** 0, 4, 6, 8, 10, 12, 20, 40 μg/mL (24 h, 48 h) | ***In vitro*:** 10 μg/mL | ***In vitro*:** isotonic medium (negative), hyperosmotic medium (550 mOsM, negative), hyperosmotic medium + Oroxylin A + 3-TYP (SIRT3 inhibitor, mechanistic control) | ***In vitro*:** No significant cytotoxicity at concentrations ≤10 μg /mL for 24 h; 48 h treatment at 8 and 10 μg /mL showed toxicity |

**Supplementary Table S4 (Continued).** Detailed Pharmacological and Experimental Parameters.

| **Ref.** | **Metabolite/Formula** | **Full Dose Range Tested** | **Minimal Effective Concentration/Dose** | **Controls** | **Toxic Side Effects** |
| --- | --- | --- | --- | --- | --- |
| (H. Chen et al., 2025) | Chlorogenic Acid | ***In vivo*:** 50 mg/kg (i.m., 1×/d, 28 d) | ***In vivo*:** 50 mg/kg (i.m.) | ***In vivo*:** Wild-type (WT) mice (negative), SOD1⁻/⁻ DED model + saline (negative), SOD1⁻/⁻ DED model + DHLA (positive) | ***In vivo*:** no significant adverse effects or toxicity reported at the administered dose |
| (Liang et al., 2023) | Salidroside | ***In vivo*:** 0.5, 2 mM (topical, 2×/d, 10 d)  ***In vitro* (mechanism):** 25, 50, 100 µM (24 h pretreatment) | ***In vivo*:** 0.5 mM (topical)  ***In vitro*:** 25 μM | ***In vivo*:** healthy mice + PBS (negative), DED model mice + PBS (negative)  ***In vitro*:** isotonic medium (negative), hyperosmotic medium (negative) | ***In vivo*:** no mentioned organ toxicity  ***In vitro*:** no damage to HCECs at concentrations up to 200 μM |
| (Lee et al., 2011) | Epigallocatechin gallate (EGCG) | ***In vivo*:** 0.01%, 0.1% (topical, 2×/d, 7 d) | ***In vivo*:** 0.1% (topical) | ***In vivo*:** healthy mice (negative), untreated DED model mice (negative), vehicle control (1% DMSO in PBS, negative) | ***In vivo*:** no cellular toxicity to the corneal epithelium was observed with 0.01% or 0.1% EGCG |
| (Hu, 2017) | Qinpi eye drop | ***In vivo*:** Eye drops (topical, 5×/d, 28 d) | ***In vivo*:** Eye drops (topical) | ***In vivo*:** normal control group (negative), untreated DED model mice (negative) | ***In vivo*:** no mentioned side effects |
| (Wang et al., 2010) | Buddleia flavonoids | ***In vitro* (mechanism):** 8.95×10⁻² mol/L medicated plasma (48 h) | ***In vitro*:** 8.95×10⁻² mol/L medicated plasma | ***In vitro*:** untreated normal cells (negative), H₂O₂-only treated cells (negative), H₂O₂ + testosterone propionate treatment (positive), H₂O₂ + flutamide (AR blocker control). | ***In vitro*:** not mentioned |
| (Shetty et al., 2020) | Resveratrol | ***In vitro* (cytotoxicity/viability):** 0.5, 1, 5, 10, 25, 50, 100, 200, 400 µM (24 h) | ***In vitro*:** 25 μM | ***In vitro*:** untreated normal HCE cells (negative), hyperosmolarity-only treated cells (negative), hyperosmolarity + recombinant Jagged1 (Notch activator, positive), hyperosmolarity + LY-411575 (Notch blocker, pathway control). | ***In vitro*:** no significant cytotoxicity at ≤25 μM; toxic at ≥50 μM (reduced cell viability) |
| (Zhao and Wei, 2024) | Polydatin | ***In vivo*:** 0.05%, 0.5% (topical, 3×/d, 7 d) | ***In vivo*:** 0.5% (topical) | ***In vivo*:** healthy rats (negative), DED model rats + saline eye drops (negative), DED model rats + H-89 (PKA inhibitor, pathway control). | ***In vivo*:** not mentioned |

**Supplementary Table S4 (Continued).** Detailed Pharmacological and Experimental Parameters.

| **Ref.** | **Metabolite/Formula** | **Full Dose Range Tested** | **Minimal Effective Concentration/Dose** | **Controls** | **Toxic Side Effects** |
| --- | --- | --- | --- | --- | --- |
| (Li et al., 2025) | Evodiamine | ***In vivo*:** 500 µM, 1 mM (topical, 4×/d, 14 d)  ***In vitro* (cytotoxicity/viability):** 0.001, 0.010, 0.100, 1.000, 10.000 µM (24 h) | ***In vivo*:** 500 μM (topical)  ***In vitro*:** 0.1 μM | ***In vivo*:** healthy mice (negative), DED model mice + vehicle (DMSO in PBS, negative), DED model mice + artificial tears (symptom relief control)  ***In vitro*:** untreated normal HCECs (negative), hyperosmolarity-only treated cells (negative), hyperosmolarity + N-acetylcysteine (antioxidant, positive), hyperosmolarity + chloroquine (autophagy inhibitor, pathway control). | ***In vivo*:** not mentioned  ***In vitro*:** no significant cytotoxicity at 0.1 μM; toxic at higher concentrations (e.g., 10 μM reduced cell viability) |
| (Chu et al., 2021) | Astragalus Ⅳ | ***In vivo*:** 5, 10 µM (topical, 4×/d, 28 d)  ***In vitro* (mechanism):** 5, 10 µM (24 h) | ***In vivo*:** 5 µM (topical)  ***In vitro*:** 5 µM | ***In vivo*:** healthy rabbits (negative), DED + solvent (negative)  ***In vitro*:** untreated HCECs (negative), BAC-only (negative) | ***In vivo*:** not mentioned  ***In vitro*:** no cytotoxicity observed |
| (Ling et al., 2022) | *Dendrobium officinale* Kimura et Migo water extract (DOW); *Dendrobium loddigesii* Rolfe water extract (DLW) | ***In vivo*:** 66.7, 200 mg/kg (oral, 1×/d, 14 d)  ***In vitro* (mechanism):** 250, 500 µg/mL (24 h) | ***In vivo*:** 66.7 mg/kg (oral)  ***In vitro*:** 250 µg/mL | ***In vivo*:** healthy rats (negative), DED + vehicle (negative), DED + CsA (positive)  ***In vitro*:** normal HKs (negative), NaCl-only (negative) | ***In vivo*:** no systemic toxicity observed  ***In vitro*:** DOW toxic at 1000 µg/mL, DLW safe at tested doses |
| (P. Liu et al., 2024a) | Linarine | ***In vivo*:** 12.5, 25, 50 mg/kg/day (gavage, 1×/d, 14 d)  ***In vitro* (mechanism):** 15 µM (24 h)  ***In vitro* (cytotoxicity):** 1, 5, 10, 15, 20 µM (24 h) | ***In vivo*:** 25 mg/kg (gavage)  ***In vitro*:** 15 µM | ***In vivo*:** healthy mice (negative), DED + vehicle (negative), DED + Sodium Hyaluronate (positive)  ***In vitro*:** normal HCECs (negative), NaCl-only (negative) | ***In vivo*:** no significant toxicity in heart, liver, spleen, lung, or kidney  ***In vitro*:** safe at tested concentrations |
| (Zhao et al., 2019) | Paeoniflorin | ***In vivo*:** 0.01%, 0.1%, 1% (topical, 5×/d, 28 d)  ***In vitro* (mechanism):** 0.01%, 0.1%, 1% (24 h) | ***In vivo*:** 1% (topical)  ***In vitro*:** 0.1% | ***In vivo*:** healthy mice (negative), DED + HM (negative)  ***In vitro*:** normal HCE cells (negative), HM-only (negative) | ***In vivo*:** not mentioned  ***In vitro*:** no cytotoxicity at tested doses |

**Supplementary Table S4 (Continued).** Detailed Pharmacological and Experimental Parameters.

| **Ref.** | **Metabolite/Formula** | **Full Dose Range Tested** | **Minimal Effective Concentration/Dose** | **Controls** | **Toxic Side Effects** |
| --- | --- | --- | --- | --- | --- |
| (Chao et al., 2016) | Lutein | ***In vitro* (mechanism):** 1, 3, 10 µM (24 h) | ***In vitro*:** 3 µM | ***In vitro*:** normal CE cells (negative), hyperosmotic medium-only (negative), hyperosmotic + 10 µM U0126 (ERK inhibitor), 10 µM SP600125 (JNK inhibitor), 10 µM SB203580 (p38 MAPK inhibitor), or 5 µM BAY 11-7082 (NF-κB inhibitor) (positive) | ***In vitro*:** no effect on cell viability at 1–10 µM |
| (Kim et al., 2018) | KIOM-2015EW | ***In vitro* (mechanism):** 0.05, 0.1, 0.2 mg/mL (24 h) | ***In vitro*:** 0.05 mg/mL | ***In vitro*:** isomolar medium (312 mOsM, negative), hyperosmolar medium (450 mOsM, negative), hyperosmolar medium + Cyclosporine A (CsA) or fluoremetholone (FML) (positive) | ***In vitro*:** no significant cytotoxicity at concentrations up to 0.3 mg/mL; higher concentrations (0.4-0.5 mg/mL) decreased cell viability |
| (Zhu et al., 2024) | Aurantio-obtusin (AO) | ***In vivo*:** 0.5% (topical, 4×/d, 10 d) | ***In vivo*:** 0.5% (topical) | ***In vivo*:** Sham + DMSO (negative), model + DMSO (negative), model + AO (treatment) | ***In vivo*:** not mentioned for topical application; oral administration can induce hepatotoxicity |
| (Park et al., 2019) | Polydatin (PD) | ***In vivo*:** 0.05%, 0.5% (topical, 3×/d, 7 d)  ***In vitro* (mechanism):** 0.1, 1, 10 µM (24 h) | ***In vivo*:** 0.5% (topical)  ***In vitro*:** 10 µM | ***In vivo*:** normal rats (negative), DED-induced rats + vehicle (negative)  ***In vitro*:** untreated cells (negative), hyperosmotic-treated cells (negative), hyperosmotic-treated cells + N-acetyl-L-cysteine (NAC) (positive for ROS) | ***In vivo*:** not mentioned, but study suggests safety for daily use  ***In vitro*:** no significant toxicity at tested concentrations |
| (Yang et al., 2021) | β-aminoarteether maleate (SM934) | ***In vivo* (SCOP model):** 0.1%, 0.5% (topical, 4×/d, 7 d (rats) or 10 d (mice))  ***In vivo* (BAC model):** 0.1%, 0.5% (topical, 4×/d, 10 d)  ***In vitro* (mechanism):** 10 µM (24 h pretreatment) | ***In vivo*:** 0.5% (topical)  ***In vitro*:** 10 µM | ***In vivo*:** normal group (negative), model group (negative), solvent group (negative), 0.1% sodium hyaluronate (SH) group (positive)  ***In vitro*:** untreated cells (negative), LPS-treated cells (negative) | ***In vivo*:** not mentioned for topical application  ***In vitro*:** not mentioned |

**Supplementary Table S4 (Continued).** Detailed Pharmacological and Experimental Parameters.

| **Ref.** | **Metabolite/Formula** | **Full Dose Range Tested** | **Minimal Effective Concentration/Dose** | **Controls** | **Toxic Side Effects** |
| --- | --- | --- | --- | --- | --- |
| (Li, 2023) | Pterostilbene-glutaricanhydride-arginine-glycine-asparticacid (PS-GA-RGD) | ***In vivo*:** 1, 2.5, 5 mg/mL (topical, 3×/d, 5 d)  ***In vitro* (mechanism):** 10 µM (16 h pretreatment, then co-culture with LPS for 8 h) | ***In vivo*:** 5 mg/mL (topical)  ***In vitro*:** 10 μM | ***In vivo*:** healthy mice (negative), DED model mice + PBS (vehicle, negative), DED model mice + PS (positive)  ***In vitro*:** untreated cells (negative), LPS-only treated cells (negative), LPS + PS treatment (positive) | ***In vivo*:** no ocular irritation, no changes in intraocular pressure in rabbits  ***In vitro*:** no cytotoxicity at 10 μM |
| (Li et al., 2023) | Gallic acid (GA) | ***In vivo*:** 1, 5, 10 mg/mL (topical, 3×/d, 5 d)  ***In vitro* (cytotoxicity):** 0–200 µM (24 h)  ***In vitro* (mechanism):** 100 µM (pretreatment 16 h for RAW264.7, 23 h for HCECs) | ***In vivo*:** 5 mg/mL (topical)  ***In vitro*:** 100 µM | ***In vivo*:** healthy mice (negative), DED model mice + PBS (vehicle, negative), DED model mice + Cyclosporine A (positive)  ***In vitro*:** untreated normal HCECs (negative), hyperosmotic-only treated HCECs (negative), LPS-only treated RAW264.7 cells (negative), hyperosmotic + Trolox or LPS + Trolox (positive) | ***In vivo*:** no ocular irritation, no effect on intraocular pressure in rabbits  ***In vitro*:** no cytotoxicity up to 100 μM |
| (Yi Han et al., 2023) | Berberine (BBR) | ***In vivo*:** 0.5, 2 mg/mL (topical, 4×/d, 7 d)  ***In vitro* (cell viability):** 1.25, 2.5, 5, 10 µM (24 h) | ***In vivo*:** 2 mg/mL (topical)  ***In vitro*:** 2.5 µM | ***In vivo*:** healthy mice (negative), DED model + vehicle (PBS, negative), DED model + Cyclosporin A (positive)  ***In vitro*:** untreated normal HCE cells (negative), hyperosmotic medium only (negative) | ***In vivo*:** not mentioned, but poor solubility noted  ***In vitro*:** no significant cytotoxicity at 1.25–10 µM; cell viability suppressed at >20 µM |
| (Zhou et al., 2020) | Genistein | ***In vitro* (mechanism/cell viability):** 50 µmol/L (30 min pretreatment, then culture under hyperosmotic conditions for 24 h) | ***In vitro*:** 50 µmol/L | ***In vitro*:** normal osmotic medium (312 mOsm/L, negative), hyperosmotic medium only (450 mOsm/L, negative) | ***In vitro*:** no cytotoxicity observed at 50 µmol/L |
| (Li D. et al., 2015) | Yangyin Runmu Pills | ***In vivo*:** 9 g/100 mL, 1×/d, 3 months (gavage) | ***In vivo*:** 9 g/100 mL (gavage) | ***In vivo*:** normal rats (negative), sham-operated rats (negative), model rats + vehicle (physiological saline, negative), model rats + Xincell eye drops (positive) | ***In vivo*:** not mentioned |

**Supplementary Table S4 (Continued).** Detailed Pharmacological and Experimental Parameters.

| **Ref.** | **Metabolite/Formula** | **Full Dose Range Tested** | **Minimal Effective Concentration/Dose** | **Controls** | **Toxic Side Effects** |
| --- | --- | --- | --- | --- | --- |
| (Li et al., 2019) | Yangyin Runmu Pills | ***In vivo*:** 0.782, 0.869, 1.738 g/(kg·d), 1×/d, 2 w (gavage) | ***In vivo*:** 0.782 g/kg (gavage) | ***In vivo*:** normal rabbits (negative), model rabbits + vehicle (physiological saline, negative), model rabbits + p38 inhibitor SB203580 (positive), model rabbits + Qiju Dihuang Pills (positive) | ***In vivo*:** not mentioned |
| (Li D. et al., 2022) | Yangyin Runmu Pills | ***In vitro* (mechanism):** Medicated serum (equiv. to 6.952 g·kg⁻¹·d⁻¹, 7 d) | ***In vitro*:** Medicated serum (equiv. to 6.952 g·kg⁻¹·d⁻¹, 7 d) | ***In vitro*:** normal cells (negative), TNF-α-induced cells (TNF-α group, negative), TNF-α-induced cells + positive drug serum (Qiju Dihuang Pills, positive) | ***In vitro*:** not mentioned |
| (Zhao et al., 2024a) | Qingxuan Runmu Decoction (QXRMY) | ***In vivo*:** 0.6 g/mL, 1×/d, 14 d (gavage) | ***In vivo*:** 0.6 g/mL (gavage) | ***In vivo*:** normal rats (blank group, negative), model rats + vehicle (PBS buffer eye drops, negative), model rats + sodium hyaluronate eye drops (positive) | ***In vivo*:** not mentioned |
| (Zhao et al., 2024b) | Qingxuan Runmu Decoction (QXRMY) | ***In vivo*:** 0.6 g/mL, 2×/d, 14 d (gavage) | ***In vivo*:** 0.6 g/mL (gavage) | ***In vivo*:** healthy rats (negative), DED model rats + vehicle (saline gavage, negative), DED model rats + Sodium hyaluronate eye drops (positive) | ***In vivo*:** not reported |
| (J. Wang et al., 2024) | Qingxuan Runmu Decoction (QXRMY) | ***In vivo*:** equivalent dose (gavage)  ***In vitro* (mechanism):** 2.5%, 5%, 10% medicated serum (equiv. to human dose, 7 d) | ***In vivo*:** equivalent dose (gavage)  ***In vitro*:** 5% medicated serum | ***In vivo*:** healthy rats (negative), DED model rats (negative)  ***In vitro*:** untreated normal HCE-2 cells (negative), hyperosmotic medium-only treated cells (negative), hyperosmotic medium + Ferrostatin-1 (Fer-1) or Deferoxamine (DFO) treatment (positive) | ***In vivo*:** not reported  ***In vitro*:** not reported |
| (Zhao et al., 2022b) | Qishen prescription† | ***In vitro* (mechanism):** 15% medicated serum (equiv. to 5.99, 11.98, 23.96 g·kg⁻¹·d⁻¹, 3 d) | ***In vitro*:** 15% medicated serum (equiv. to 11.98 g·kg⁻¹·d⁻¹) | ***In vitro*:** untreated normal HCECs (negative), hypertonic medium-only treated cells (negative), hypertonic medium + Sodium hyaluronate eye drops (positive) | ***In vitro*:** not reported |

**Supplementary Table S4 (Continued).** Detailed Pharmacological and Experimental Parameters.

| **Ref.** | **Metabolite/Formula** | **Full Dose Range Tested** | **Minimal Effective Concentration/Dose** | **Controls** | **Toxic Side Effects** |
| --- | --- | --- | --- | --- | --- |
| (Zhao et al., 2022a) | Qishen prescription† | ***In vitro* (mechanism):** 15% medicated serum (equiv. to 5.99, 11.98, 23.96 g·kg⁻¹·d⁻¹, 3 d) | ***In vitro*:** 15% medicated serum (equiv. to 11.98 g·kg⁻¹·d⁻¹) | ***In vitro*:** untreated normal HCECs (negative), hypertonic medium-only treated cells (negative), hypertonic medium + Sodium hyaluronate eye drops (positive) | ***In vitro*:** not reported |
| (Luo et al., 2024) | Runmu Ling granules (RMLG) | ***In vivo*:** 0.75, 1.5 g/kg (gavage, 1×/d, 2 w)  ***In vitro* (mechanism):** 2.5%, 5%, 10% medicated serum (3 h) | ***In vivo*:** 0.75 g/kg (gavage)  ***In vitro*:** 5% medicated serum | ***In vivo*:** healthy rats (negative), DED model rats + vehicle (pure water gavage, negative), DED model rats + MCC950 (positive)  ***In vitro*:** untreated normal HCECs (negative), BAC-only treated cells (negative), BAC + MCC950 (NLRP3 inhibitor) treatment (positive) | ***In vivo*:** not reported  ***In vitro*:** not reported |
| (Song et al., 2023) | Runmu Ling Eye Drops | ***In vivo*:** 0.51 g/mL (topical, 20 min/d, 14 d) | ***In vivo*:** 0.51 g/mL (topical) | ***In vivo*:** normal rabbits (negative), DED model rabbits + no treatment (negative) | ***In vivo*:** not mentioned |
| (Liu T. et al., 2024) | Runmu Xiaoyao Powder | ***In vivo*:** 7.25, 14.5, 29 g/kg (gavage, 2×/d, 14 d) | ***In vivo*:** 14.5 g/kg (gavage) | ***In vivo*:** normal mice (negative), DED model mice + deionized water gavage (negative), DED model mice + Sodium hyaluronate eye drops (positive) | ***In vivo*:** not mentioned |
| (P. Liu et al., 2024b) | Modified Danzhi Xiaoyao Powder (MDXP) | ***In vivo*:** 6.24, 12.48, 24.96 g/kg (gavage, 2×/d, 14 d)  ***In vitro*:** N/S | ***In vivo*:** 12.48 g/kg (gavage)  ***In vitro*:** N/S | ***In vivo*:** normal mice (negative), DED model mice + vehicle (deionized water, negative), DED model mice + Sodium hyaluronate eye drops (positive)  ***In vitro*:** untreated normal cells (negative), hypertonic medium-only HCECs (negative), LPS-only RAW264.7 and THP-1 cells (negative) | ***In vivo*:** not mentioned  ***In vitro*:** not mentioned |

**Supplementary Table S4 (Continued).** Detailed Pharmacological and Experimental Parameters.

| **Ref.** | **Metabolite/Formula** | **Full Dose Range Tested** | **Minimal Effective Concentration/Dose** | **Controls** | **Toxic Side Effects** |
| --- | --- | --- | --- | --- | --- |
| (Chen et al., 2024) | Sihuang Qingling Liquid (SHQLY) | ***In vitro* (mechanism/cytotoxicity):** 5, 10, 20, 40, 80 µg/mL (18 h) | ***In vitro*:** 10 μg/mL | ***In vitro*:** untreated normal RAW264.7 cells (negative), LPS-only treated cells (negative) | ***In vitro*:** no significant cytotoxicity at 5–20 μg/mL; slight inhibition at 40–80 μg/mL (not significant) |
| (Chen L. et al., 2023) | Modified Siwei Dafa Powder | ***In vivo*:** 5.89 g/kg (gavage, 1×/d, 7 d) | ***In vivo*:** 5.89 g/kg (gavage) | ***In vivo*:** normal rats (negative), DED model rats + no treatment (negative), DED model rats + Moxibustion treatment (treatment control) | ***In vivo*:** not mentioned |
| (Yang J. et al., 2023) | Huashi Runjing Decoction | ***In vivo*:** 0.93 g/mL (gavage, 1×/d, 28 d) | ***In vivo*:** 0.93 g/mL (gavage) | ***In vivo*:** healthy rats (negative), sham-operated rats (negative), model rats + vehicle (saline by gavage and saline eye drops, negative), model rats + sodium hyaluronate eye drops (positive) | ***In vivo*:** not mentioned |
| (Wu et al., 2022) | Zhenzhu Mingmu Eye Drops (ZMED) | ***In vitro* (mechanism/cell viability):** 50×, 100×, 1000× dilution (24 h) | ***In vitro*:** 50× dilution | ***In vitro*:** untreated normal HCEC (negative), TNF-α+IFN-γ-treated cells (negative) | ***In vitro*:** not mentioned |
| (Du, 2018) | Xiaosheng Granules | ***In vivo*:** 3.6 mg/g (gavage, 1×/d, 4 w)  ***In vitro* (mechanism):** 1, 10, 100, 400 µg/mL (24 h) | ***In vivo*:** 3.6 mg/g  ***In vitro*:** 100 µg/mL | ***In vivo*:** healthy mice (negative), DED model mice + vehicle (negative)  ***In vitro*:** normal HCEC cells (negative), Hyperosmotic or inflammatory stress only (negative) | ***In vivo*:** not mentioned  ***In vitro*:** not mentioned |
| (Shi X. et al., 2023) | Erzhi Pills | ***In vivo*:** 3, 6, 12 g/kg (gavage, 2×/d, 14 d) | ***In vivo*:** 12 g/kg (gavage) | ***In vivo*:** healthy mice (negative), DED model mice + vehicle (0.9% NaCl, negative) | ***In vivo*:** not mentioned |
| (Dong, 2024) | Zhenshi Guben Liquid | ***In vivo*:** 25.96, 51.92, 103.84 g/kg (gavage, 1×/d, 4 w) | ***In vivo*:** 51.92 g/kg | ***In vivo*:** healthy mice (negative), DED model mice + vehicle (saline, negative), DED model mice + Pranoprofen eye drops (positive) | ***In vivo*:** not mentioned |
